# One-Pot Synthesis of Isoxazole-Fused Tricyclic Quinazoline Alkaloid Derivatives via Intramolecular Cycloaddition of Propargyl-Substituted Methyl Azaarenes under Metal-Free Conditions

**DOI:** 10.3390/molecules28062787

**Published:** 2023-03-20

**Authors:** Zhuo Wang, Yuhan Zhao, Jiaxin Chen, Mengyao Chen, Xuehan Li, Ting Jiang, Fang Liu, Xi Yang, Yuanyuan Sun, Yanping Zhu

**Affiliations:** 1 Key Laboratory of Molecular Pharmacology and Drug Evaluation, Ministry of Education, Collaborative Innovation Center of Advanced Drug Delivery System and Biotech Drugs in Universities of Shandong, School of Pharmacy, Yantai University, Yantai 264005, China; 2 Anhui Laboratory of Molecule-Based Materials, College of Chemistry and Materials Science, Anhui Normal University, Wuhu 241000, China

**Keywords:** *tert*-butyl nitrite (TBN), tricyclic quinazoline alkaloid, isoxazole, methyl azaarenes, intramolecular cycloaddition

## Abstract

A practical method was developed for the convenient synthesis of isoxazole-fused tricyclic quinazoline alkaloids. This procedure accesses diverse isoxazole-fused tricyclic quinazoline alkaloids and their derivatives via intramolecular cycloaddition of methyl azaarenes with *tert*-butyl nitrite (TBN). In this method, TBN acts as the radical initiator and the source of N–O. Moreover, this protocol forms new C–N, C–C, and C–O bonds via sequence nitration and annulation in a one-pot process with broad substrate scope and functionalization of natural products.

## 1. Introduction

**Figure 1:**
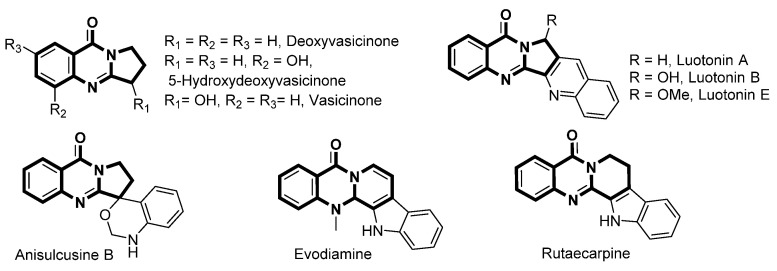
Representative molecules containing tricyclic quinazoline.

Quinazolinones are a well-known family of *N*-heterocyclic compounds with bioactivities [1,2,3,4,5,6] on multiple fronts and occur often in natural products [7,8,9,10]. Recently, a group of special tricyclic quinazoline alkaloids, such as 2,3-dihydropyrrolo[2,1-*b*]quinazolin-9(1*H*)-ones, have attracted increased attention from medicinal chemists and biologists for their bioactivity levels in areas such as antitumor, antibacterial, anti-inflammatory and anti-acetylcholinesterase activities [11,12,13,14,15,16]. Key examples include deoxyvasicinone and vasicinone isolated from the *adhatoda vasica* medicinal plant, with pronounced anti-inflammatory, antimicrobial, and antidepressant activities (Figure 1) [17,18,19,20]. Luotonin A and evodiamine are promising anticancer natural products that act as topoisomerase I inhibitors [21,22]. Anisulcusine B, isolated from *anisotes trisulcus,* showed moderate cytotoxic effect against human hepatoma (HuH7) cells [23]. Rutaecarpine is an inhibitor of COX-2 with an IC_50_ value of 0.28 μM [24].

**Scheme 1:**
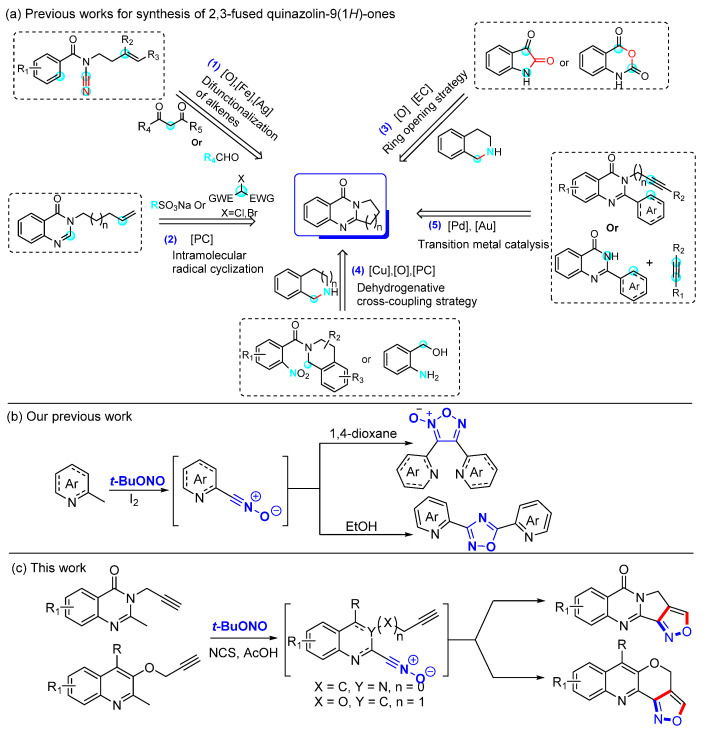
Strategies for the synthesis of 2,3-fused quinazolin-4(3*H*)-ones. (**a**) Current methods to synthesize tricyclic quinazoline alkaloid. (**b**) Our previous work forming 1,3-dipolar nitrile oxide from methyl azaarenes via TBN. (**c**) Our current work to form 1,3-dipolar nitrile oxide from propargyl-substituted methyl azaarenes via TBN.

The ubiquitous nature and importance in pharmaceutical research have led to several reported synthetic methodologies for 2,3-fused quinazoline-4(3*H*)-ones and their derivatives (Scheme 1a) [25,26,27,28]: (1) *N*-Cyanamide alkenes react with aldehydes or diketones via intramolecular cyclization difunctionalization [18], [29,30,31]; (2) intramolecular radical cyclization of N3-Alkenyl-tethered quinazolinone [32,33,34,35]; (3) oxidative cyclization of isatins or isatoic anhydrides with cyclic amines by ring opening strategy [36,37]; (4) cyclic amines undergo a dehydrogenation process including intermolecular and intramolecular cyclization [38,39,40,41]; (5) 2-Arylquinazolinones undertake a cyclization reaction via transition metal catalysis [42,43].

Isoxazole is a five-membered heterocycle with two adjacent heteroatoms and a cyclic conjugated system, which could react with target proteins via multiple noncovalent bonds [44,45,46]. This feature of isoxazole makes it a key pharmaceutical fragment in many drugs [47,48,49]. In recent years, *tert*-butyl nitrite (TBN) has acted as a metal-free radical initiator and source of N-O [50,51,52,53,54,55,56,57,58,59], which has attracted extensive attention in the construction of isoxazoles [60,61,62,63,64,65,66,67,68,69]. Despite these excellent methods, there are few reports on the construction of skeletal cores containing isoxazole and tricyclic quinazoline alkaloids or their derivatives. Recent literature reports have focused primarily on the formation of isoxazole-substituted methyl azaarenes from intermolecular reactions via *tert*-butyl nitrite. For example, Zhang and co-workers reported a graceful method for the synthesis of 3-quinolinyl-isoxazoles from 2-methyl quinolines, ethyl propiolate, and TBN under metal-free conditions [70]. Yang et al. developed copper-catalyzed 1,3-dipolar cycloaddition of alkylazaarenes with alkynes to synthesize isoxazoles, in which the nitro sources were generated in situ from KNO_3_ and K_2_S_2_O_8_ [71,72]. Song et al. developed an elegant protocol to synthesize isoxazoles via divergent annulation of sulfoxonium ylides with *t*-BuONO [73]. To the best of our knowledge, the formation of isoxazole fusing 2,3-dihydropyrrolo[2,1-*b*]quinazoliones or other tricyclic quinazoline alkaloids remains challenging and difficult, with no synthetic procedures previously reported.

Recently, our group had successfully demonstrated an efficient synthetic method to synthesize furoxans from methyl azaarene and *t*-BuONO [74]. Inspired by these brilliant works, herein we disclose an efficient and practical method for the synthesis of isoxazole-fused quinazolinones or quinolines from 2-methyl-3-(prop-2-yn-1-yl)quinazolin-4(3*H*)-ones and 2-methyl-3-(prop-2-yn-1-yloxy)quinolines with TBN via metal-free conditions in a single reaction (Scheme 1c).

## 2. Results and Discussion

### 2.1. Reaction Optimization

We selected 2-methyl-3-(prop-2-yn-1-yl)quinazolin-4(3*H*)-one (**1a**) as the model substrate to optimize the reaction conditions and to verify the feasibility of our assumption (Table 1). We initially treated 2-methyl-3-(prop-2-yn-1-yl)quinazolin-4(3*H*)-one and TBN in DMSO at 80 °C for 6 h, which yielded the desired 4*H*,6*H*-isoxazolo[3′,4′:3,4]pyrrolo[2,1-*b*]quinazolin-6-one (**2a**) in a 45% yield (Table 1, entry 1). We varied the solvent (DMF, 1,4-dioxane, and acetonitrile; Table 1, entries 2–4). Acetonitrile afforded the desired product in a 64% yield. Temperature modification (Table 1, entries 4–6) confirmed 100 °C as the optimal reaction temperature. Adding TBN (4.5, 5, 5.5, and 6 eq.) was examined, which resulted in yields of 61%, 65%, 72%, and 70%, respectively, indicating that 5.5 eq. of TBN optimized the yield. Conducting the reaction under argon increased the yield of **2a** to 75% (Table 1, entry 8). Adding 0.5 eq. of NCS (N-chlorosuccinimide) increased the yield to 76% (entry 9). We also screened several acids to determine their influence on the annulation reaction. Using trifluoroacetic acid (TFA) decreased the yield of the desired product 2a to 46% (entry 10); however, adding acetic acid increased the yield increased to 80% (entry 11). Aliphatic and aromatic carbonic acids did not promote this reaction effectively (entries 12–17).

### 2.2. Substrate Scope

**Figure 2:**
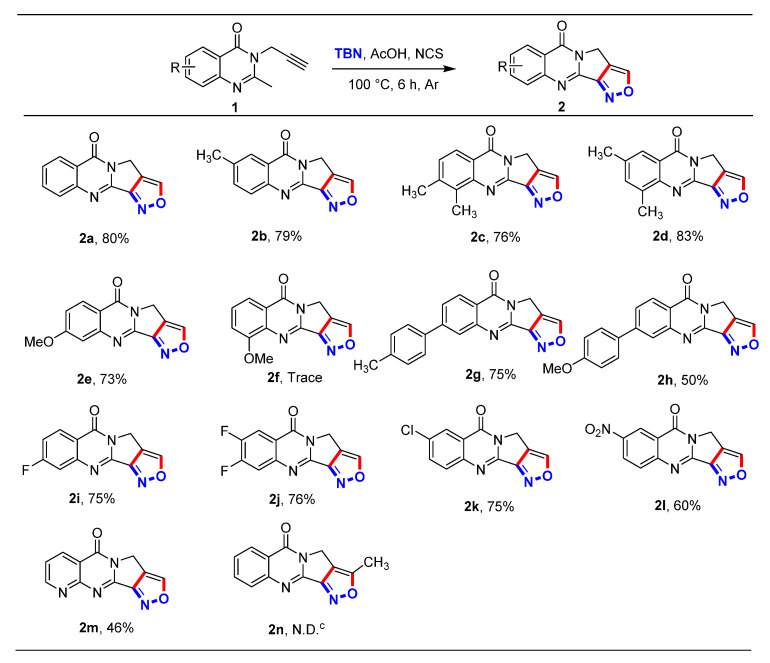
Scope of 2-methyl-3-(prop-2-yn-1-yl)quinazolin-4(3*H*)-one ^a,b^. ^a^ Reaction conditions: **1** (0.2 mmol), TBN (1.1 mmol), NCS (0.1 mmol), AcOH (0.1 mmol) were stirred in acetonitrile (2 mL) at 100 °C for 6 h under Ar. ^b^ Isolated yields. ^c^ Not detected.

After the optimum conditions were established, the substrate scope of 2-methyl-3-(prop-2-yn-1-yl)quinazolin-4(3*H*)-one for the formation of 4*H*,6*H*-isoxazolo[3′,4′:3,4]pyrrolo[2,1-*b*]quinazolin-6-one **2** was evaluated. As shown in Figure 2, a series of electron-donating groups on the phenyl ring of **1**, such as the methyl groups at the C-4, C-4,6, C-5,6 position (**1b**–**1d**) and the methoxyl group at the C-5 position (**1e**), could participate in this reaction to afford the corresponding products in 73–83% yields (**2b**–**2e**). To our delight, **1** with a phenyl group in the position of C-5 (**1g**–**1h**) gave the corresponding products smoothly in 75% and 50% yields, respectively. However, **1f** was not tolerated in the reaction under the optimized conditions, giving the desired product in a very low yield. Meanwhile, electron-drawing groups on the phenyl ring of **1** proceeded well via our protocol, such as the fluoro group at the position C-5, C-4,5 (**1i**–**1j**), the chloro and nitro group at the C-4 position (**1k**–**1l**), leading to the desired products in good yields (60–76%). To our delight, heteroatom-contained substrate **1m** was also suitable for this reaction to afford the desired product **2m** in 46% yield. In addition, the substrates of the substituted alkynyl group were also tested; those results showed that alkynyl substituted substrate (**1n**) was unsuitable for this reaction.

**Figure 3:**
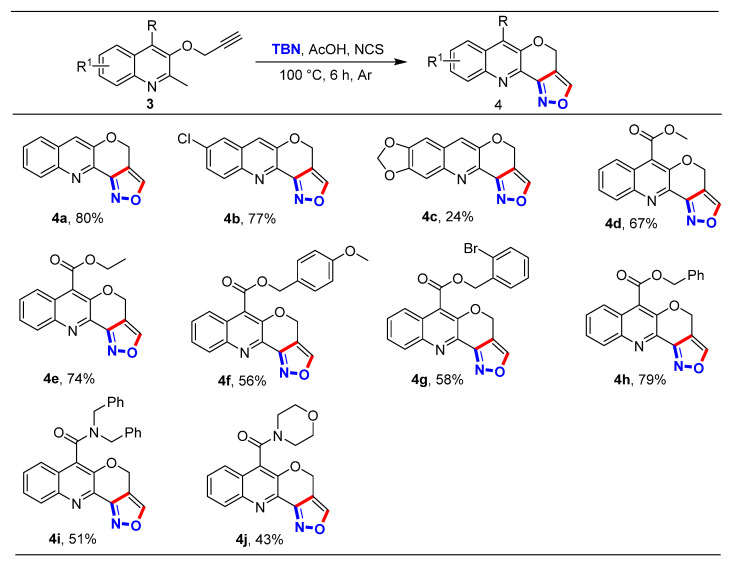
Scope of 2-methyl-3-(prop-2-yn-1-yloxy)quinoline ^a,b^. ^a^ Reaction conditions: **3** (0.2 mmol), TBN (1.1 mmol), NCS (0.1 mmol), AcOH (0.1 mmol) were stirred in acetonitrile (2 mL) at 100 °C for 6 h under Ar. ^b^ Isolated yields.

To further investigate the universality of our method, a variety of substituted 2-methyl-3-(prop-2-yn-1-yloxy)quinolines were surveyed. As shown in Figure 3, 2-methyl-3-(prop-2-yn-1-yloxy)quinoline (**3a**) was smoothly transformed into the corresponding product with a yield of 80%. Substrates of substituted groups on the phenyl ring **3b** and **3c** were applied to this reaction, which yielded products **4b** and **4c** in 77% and 24% yields, respectively. Then, ester-substituted substrates in the C-4 position were also screened, and all substrates were transformed smoothly to obtain the desired products in moderate to good yields (**4d**–**4h**, 56–79%). Moreover, different amide-substituted substrates on the C-4 position (**3i**–**3j**) were tested, processing the corresponding products **4i** and **4j** in 43% and 51% yields, respectively.

**Figure 4:**
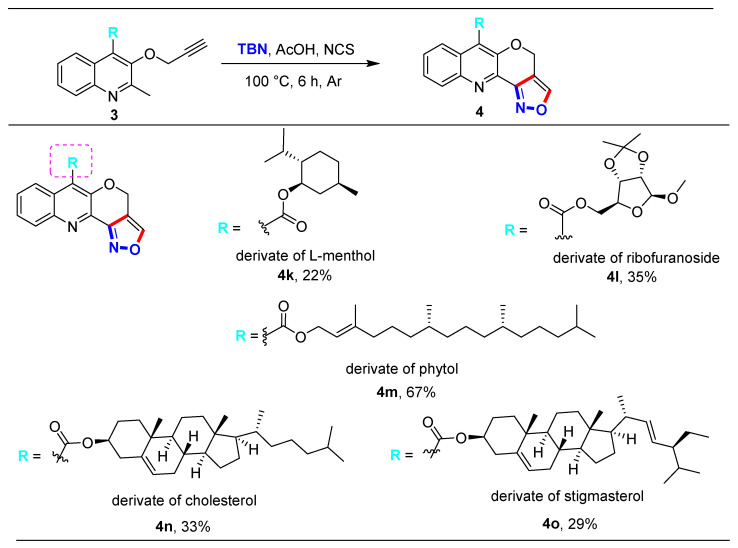
Modification of natural products ^a,b^. ^a^ Reaction conditions: **3** (0.2 mmol), TBN (1.1 mmol), NCS (0.1 mmol), AcOH (0.1 mmol) were stirred in acetonitrile (2 mL) at 100 °C for 6 h under Ar. ^b^ Isolated yields.

Furthermore, to evaluate the application of the present reaction. Several natural products were modified via our protocol. As shown in Figure 4, 2-methyl-3-(prop-2-yn-1-yloxy)quinoline-4-carboxylic acid reacted with natural alcohols to generate ester derivates **3**. The L-menthol and sugar methyl 2,3-*O*-isopropylideneisopropylidene-β-*D*-ribofuranoside could be involved in this protocol, giving the desired products (**4k**–**4l**) in 22–35% yields. The **3m** phytol derivate was smoothly transformed into the corresponding product with a yield of 67%. Two natural steroids, cholesterol and stigmasterol, were also screened, affording the corresponding products (**4n**–**4o**) in 29–33% yields.

### 2.3. Mechanism Experiments

**Scheme 2:**
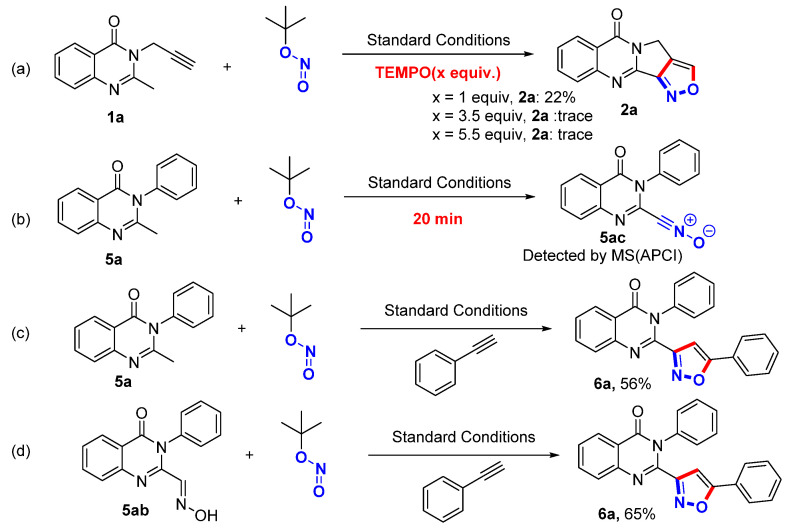
Control experiments. (**a**) Radical capture. (**b**) Formation of intermediate **5ac.** (**c**) Formation of isoxazole **6a** via intermolecular cycloaddition under standard conditions. (**d**) Formation of **6a** from intermediate **5ab** under standard conditions.

**Scheme 3:**
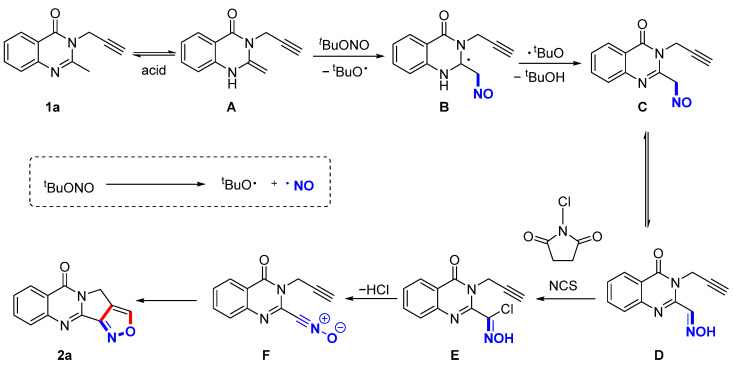
Proposed mechanism.

Several control experiments were carried out to explore the reaction mechanism (Scheme 2). The reaction was halted completely with only trace amounts of **2a**, when 3.5 equivalent of radical scavenger 2,2,6,6-tetramethyl-1-piperidinyl (TEMPO) was added to the standard reaction. These results indicated that the reaction was conducted possibly through a radical pathway. Then, 2-methyl-3-(prop-2-yn-1-yl)quinazolinone (**1a**) and TBN were reacted under standard conditions for 20 min to address the possible intermediates. Only **2a** 4*H*,6*H*-isoxazolo-pyrrolo[2,1-*b*]quinazolin-6-one was detected by MS (APCI), because the intermediate nitrile oxide **F** (Scheme 3) shares the same relative molecular mass as **2a**. Then, we tried other ways to prove them by conducting substrates **5a** 2-methyl-3-phenyl quinazoline-4(3*H*)-one under standard conditions for 20 min to detect **5ac** nitrile oxides via MS (APCI) (see Supplementary Materials). A group of intermolecular reactions was used to explore the reaction mechanism further by using **5a**, **5ab**, and phenylacetylene. Under optimal conditions, the desired product **6a** was afforded in yields of 56% and 65%, respectively. These results disclosed that nitrile oxide was the potential intermediate for this protocol.

Based on the evidence presented above and the related literature [75,76,77], a plausible reaction pathway was proposed (Scheme 3). Firstly, TBN was transformed to NO and t-BuO radicals via thermal homolysis. 2-Methyl-3-(prop-2-yn-1-yl)quinazolin-4(3*H*)-one (**1a**) could be transformed into intermediate isomer **A** via acid promotion, which reacts with TBN to produce intermediate **B** and releases a *tert*-butoxy radical. Then, intermediate **C** from oxidation of intermediate **B** undergoes tautomerization to generate oxime **D**, which can be further converted into intermediate **E** via NCS. Subsequently, the intermediate **E** can be oxidized to afford the nitrile oxides **F**, which could be transformed into the desired product **2a** through 1,3-dipolar cycloaddition.

## 3. Materials and Methods

### 3.1. General Information

Analytical thin layer chromatography (TLC) was performed using pre-coated silica gel HF254 glass plates. Column chromatography was performed using silica gel (200–300 mesh). Nuclear magnetic resonance (NMR) spectra were recorded on a Bruker Advance 500 MHz spectrometer at ambient temperature using DMSO-*d*_6_ or CDCl_3_ as the solvent with tetramethylsilane (TMS) as the internal standard at room temperature (^1^H *δ* 7.26 ppm and ^13^C{^1^H} *δ* 77.0 ppm for CDCl_3_; ^1^H *δ* 2.50 ppm and ^13^C{^1^H} *δ* 39.5 ppm for DMSO-*d_6_*). Chemical shifts (δ) are reported in ppm, relative to the internal standard of tetramethylsilane (TMS). The coupling constants (J) are quoted in hertz (Hz). Resonances are described as s (singlet), d (doublet), t (triplet), q (quartet), m (multiplet), br (broad) or combinations thereof. High-resolution mass spectra were obtained on Thermo Scientific Q-Exactive (ESI mode). Melting points were determined using SGW X-4 apparatus and were not corrected.

### 3.2. Synthetic Procedures

**Scheme 4:**
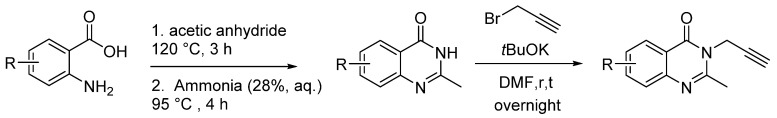
General procedure for synthesis of 2-methyl-3-(prop-2-yn-1-yl)quinazolin-4(3*H*)-one **1a**–**1l**.

Compounds **1a**–**1l** were prepared according to the referenced literature (Scheme 4) [78]. To corresponding 2-aminobenzoic acid (1 mmol) was added acetic anhydride (5 mmol), and the mixture was warmed to 120 °C for 3 h with stirring. The mixture was then concentrated in vacuo (50 °C) to remove excess acetic anhydride (bp 138 °C) to give a dry solid. Ammonium hydroxide (28% NH_3_, 100 mL) was added, and the mixture was heated to 95 °C for 4 h. The mixture was cooled, vacuum filtered and the resultant solid washed with water, saturated with NaHCO_3_ solution and more water. The 3-bromopropyne (1.5 equiv.) was dropped into the solution of the obtained solid, *t-*BuOK (1.3 equiv.) and DMF under Argon atmosphere at 0 °C to r.t overnight. After the reaction was completed, 50 mL water was added to the mixture and then extracted with EtOAc 3 times (3 × 50 mL). The extract was dried over anhydrous Na_2_SO_4_ and concentrated under reduced pressure. The crude residues were purified by column chromatography using ethyl acetate/petroleum ether mixture to obtain the corresponding products [79].

**Scheme 5:**
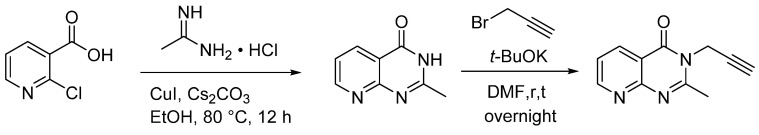
General procedure for synthesis of 2-methyl-3-(prop-2-yn-1-yl)pyrido[2,3-*d*]pyrimidin-4(3*H*)-one **1m**.

Compound **1m** was prepared according to the referenced literature with some modification (Scheme 5) [80]. A Schlenk flask was charged with a magnetic stirrer, evacuated and backfilled with argon. 2-Chloronicotinic acid (0.5 mmol) and acetamidine hydrochloride (0.75 mmol) in EtOH (3 mL) were added under Argon atmosphere. After 10 min of stirring, Cs_2_CO_3_ (1 mmol) was added to the flask. Then, 15 min later, CuI (0.1 mmol) was added to the flask. The mixture was stirred at 80 °C for 12 h. After completion of the reaction, the mixture was filtered, and the solvent of the filtrate was removed with the aid of a rotary evaporator. The residue was purified by column chromatography on silica gel to provide the desired quinazolinone. The 3-Bromopropyne (1.5 equiv.) was dropped into the solution of the obtained solid, *t*-BuOK (1.3 equiv.), and DMF under argon atmosphere at 0 °C to overnight. After the reaction was completed, 50 mL water was added to the mixture and then extracted with EtOAc 3 times (3 × 50 mL). The extract was dried over anhydrous Na_2_SO_4_ and concentrated under reduced pressure. The crude residues were purified by column chromatography using ethyl acetate/petroleum ether mixture to obtain the desired products.

**Scheme 6:**
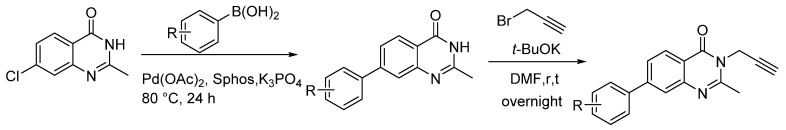
General procedure for synthesis of 2-methyl-7-phenyl-3-(prop-2-yn-1-yl)quinazolin-4(3*H*)-one **1g**–**1h**.

7-Chloro-2-methylquinazolin-4(3*H*)-one was prepared from the above procedure. A Schlenk flask was charged with a 7-chloro-2-methylquinazolin-4(3*H*)-one (0.3 mmol), corresponding to phenylboronic acid (0.45 mmol), Pd(OAc)_2_ (0.05 mmol), Sphos (0.03 mmol) and K_3_PO_4_ (2.4 mmol), and was heated in toluene (2 mL) at 80 °C for 24 h. After the reaction was completed, 50 mL water was added to the mixture and then extracted with EtOAc 3 times (3 × 50 mL). The extract was dried over anhydrous Na_2_SO_4_ and concentrated under reduced pressure. The crude residues were purified by column chromatography using ethyl acetate/petroleum ether mixture to obtain the corresponding products [81]. The 3-bromopropyne (1.5 equiv.) was dropped into the solution of the obtained solid, *t*-BuOK (1.3 equiv.), and DMF under argon atmosphere at 0 °C to r.t overnight. After the reaction was completed, 50 mL water was added to the mixture and then extracted with EtOAc 3 times (3 × 50 mL). The extract was dried over anhydrous Na_2_SO_4_ and concentrated under reduced pressure. The crude residues were purified by column chromatography using ethyl acetate/petroleum ether mixture to obtain the desired products (Scheme 6).

#### 3.2.1. Typical Procedure (TP 1) for the Synthesis of **2** and **4** Taking **2a** as an Example

A sealed tube charged with 2-methyl-3-(prop-2-yn-1-yl)quinazolin-4(3*H*)-one (**1a**) (0.2 mmol), TBN (1.1 mmol), NCS (0.1 mmol) and AcOH (0.1mmol) were heated in acetonitrile (2 mL) at 100 °C for 10 h under argon atmosphere. After the reaction was completed, 50 mL water was added to the mixture and then extracted with EtOAc 3 times (3 × 50 mL). The extract was dried over anhydrous Na_2_SO_4_ and concentrated under reduced pressure. The crude residues were purified by column chromatography using ethyl acetate/petroleum ether mixture to obtain the corresponding products **2a**.

**Scheme 7:**
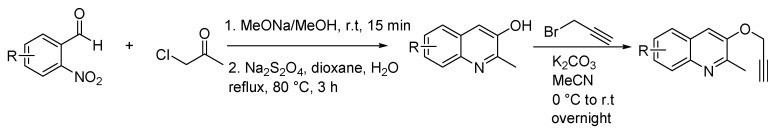
General procedure for synthesis of 2-methyl-3-(prop-2-yn-1-yloxy)quinoline **4a**–**4c**.

Compound **4a**–**4c** were prepared according to the referenced literature (Scheme 7) [82]. A solution of MeONa (1.5 mmol) in MeOH was added to a stirred solution of the corresponding 2-nitrobenzaldehyde (1.5 mmol) and a chloracetone (1.5 mmol) in MeOH (3.5 mL) at room temperature overnight. After the reaction was completed, the resulting precipitate was filtered off, the mixture was quenched carefully with water (3 × 50 mL) and with saturated NH_4_Cl (1 × 10 mL), and the product was isolated as a corresponding (2-(2-nitrophenyl)oxiran-1-yl)(aryl)methanone. A solution of Na_2_S_2_ O_4_ (5 mmol) in H_2_O (65 mL) was added to a solution of (2-(2-nitrophenyl)oxiran-1-yl)(aryl)methanone (1 mmol) in dioxane (65 mL). The reaction mixture was allowed to cool down to room temperature after reflux for 3 h and was poured into water (500 mL). The resulting precipitate was filtered off, washed with water (2 × 50 mL) and dried in air to give the corresponding 2-arylquinolin-3-ols. The 3-bromopropyne (3 equiv.) was dropped into the solution of the obtained solid, K_2_CO_3_ (3 equiv.), and acetonitrile under argon atmosphere at 0 °C to r.t overnight. After the reaction was completed, 50 mL water was added to the mixture and then extracted with EtOAc 3 times (3 × 50 mL). The extract was dried over anhydrous Na_2_SO_4_ and concentrated under reduced pressure. The crude residues were purified by column chromatography using ethyl acetate/petroleum ether mixture to obtain the desired products [83,84].

**Scheme 8:**
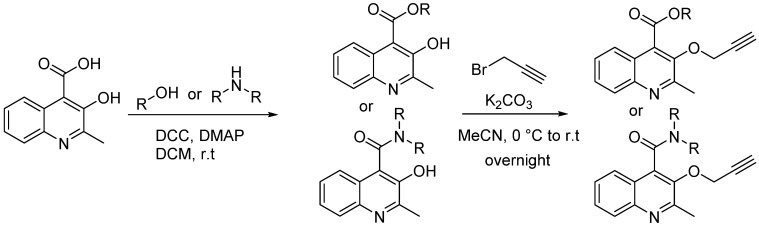
General procedure for synthesis of 2-methyl-3-(prop-2-yn-1-yloxy)quinoline-4-carboxylate **3d**–**3o**.

To a solution of 3-hydroxy-2-methyl-4-quinolinecarboxylic acid (1.1 equiv.), ROH or secondary amine (1.0 equiv.), DCC (1.1 equiv.) and DMAP (0.1 equiv.) were added in CH_2_Cl_2_ overnight to obtain 3-hydroxy-2-methylquinoline-4-carboxylate [85]. The 3-bromopropyne (3 equiv.) was dropped into the solution of the obtained solid, K_2_CO_3_ (3 equiv.), and acetonitrile under argon atmosphere at 0 °C to r.t overnight. After the reaction was completed, 50 mL water was added to the mixture and then extracted with EtOAc 3 times (3 × 50 mL). The extract was dried over anhydrous Na_2_SO_4_ and concentrated under reduced pressure. The crude residues were purified by column chromatography using ethyl acetate/petroleum ether mixture to obtain the desired products (Scheme 8).

**Scheme 9:**
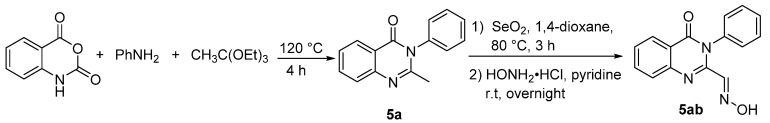
Procedure for synthesis of (*E*)-4-oxo-3-phenyl-3,4-dihydroquinazoline-2-carbaldehyde oxime (**5ab**).

Compound **5ab** was prepared according to the referenced literature (Scheme 9) [86,87,88]. A mixture of isatoic anhydride (2 mmol), aniline (2 mmol) and triethyl orthoacetate (2 mmol) was reacted under 120 °C for 4 h. After completion of the reaction, the crude reaction mixture was recrystallized from EtOH to obtain analytically pure product **5a**. Then, **5a** (0.5 mmol) and SeO_2_ (6.5 mmol) were stirred at 80 °C in 1,4-dioxane for 3 h to corresponding aldehyde. A solution of the corresponding aldehyde (0.5 mmol) in pyridine (6 mL) was added to the solution of hydroxylamine hydrochloride (0.5 mmol), keeping the reaction mixture overnight. Then, the mixture was filtered and washed water to obtain the **5ab**.

#### 3.2.2. Typical Procedure (TP 2) for the Synthesis of **6a**

A sealed tube charged with 2-methyl-3-phenyl quinazoline-4(3*H*)-one (**5a**) or (*E*)-4-oxo-3-phenyl-3,4-dihydroquinazoline-2-carbaldehyde oxime (**5ab**) (0.2 mmol), phenylacetylene (0.22 mmol), TBN (1.1 mmol), NCS (0.1 mmol) and AcOH (0.1mmol) was heated in acetonitrile (2 mL) at 100 °C for 10 h under argon atmosphere. After the reaction was completed, 50 mL water was added to the mixture and then extracted with EtOAc 3 times (3 × 50 mL). The extract was dried over anhydrous Na_2_SO_4_ and concentrated under reduced pressure. The crude residues were purified by column chromatography using ethyl acetate/petroleum ether mixture to obtain the corresponding products **6a**.

### 3.3. Characterization of Products

**4*H*,6*H*-Isoxazolo[3′,4′:3,4]pyrrolo[2,1-*b*]quinazolin-6-one (2a)**, 40 mg, brown solid, m.p.: over 280 °C. ^1^H NMR (500 MHz, DMSO-*d*_6_) *δ* 9.22 (s, 1H), 8.26 (dd, *J* = 8.0, 1.5 Hz, 1H), 7.93 (td, *J* = 7.6, 7.0, 1.5 Hz, 1H), 7.88 (d, *J* = 8.1 Hz, 1H), 7.69–7.62 (m, 1H), 5.03 (s, 2H). ^13^C NMR (126 MHz, DMSO-*d*_6_) *δ* 163.4, 159.7, 154.7, 150.0, 145.3, 134.7, 127.9, 126.0, 120.8, 119.7, 43.3. HRMS (ESI): *m*/*z* [M+H]^+^ calcd for C_12_H_7_N_3_O_2_: 226.0611; found: 226.0610.

**8-Methyl-4*H*,6*H*-isoxazolo[3′,4′:3,4]pyrrolo[2,1-*b*]quinazolin-6-one (2b)**, 38 mg, white solid, m.p.: over 280 °C. ^1^H NMR (500 MHz, DMSO-*d*_6_) *δ* 9.20 (s, 1H), 8.06 (s, 1H), 7.76 (q, *J* = 8.4 Hz, 2H), 5.02 (s, 2H), 2.50 (s, 9H). ^13^C NMR (126 MHz, DMSO-*d*_6_) *δ* 163.3, 159.6, 154.6, 146.0, 144.5, 137.9, 135.9, 127.8, 125.3, 120.6, 119.6, 43.2, 20.9. HRMS (ESI): *m*/*z* [M+H]^+^ calcd for C_13_H_9_N_3_O_2_: 240.0768; found: 240.0763.

**9,10-Dimethyl-4*H*,6*H*-isoxazolo[3′,4′:3,4]pyrrolo[2,1-*b*]quinazolin-6-one (2c)**, 38 mg, white solid, m.p.: 259–260 °C. ^1^H NMR (500 MHz, DMSO-*d*_6_) *δ* 9.20 (s, 1H), 7.98 (d, *J* = 8.1 Hz, 1H), 7.44 (d, *J* = 8.1 Hz, 1H), 4.99 (s, 2H), 2.55 (s, 3H), 2.43 (s, 3H). ^13^C NMR (126 MHz, DMSO-*d_6_*) *δ* 163.5, 159.9, 154.6, 146.2, 144.1, 143.4, 134.2, 129.5, 122.8, 119.6, 118.7, 43.0, 20.5, 13.1. HRMS (ESI): *m*/*z* [M+H]^+^ calcd for C_14_H_11_N_3_O_2_: 254.0924; found: 254.0924.

**8,10-Dimethyl-4*H*,6*H*-isoxazolo[3′,4′:3,4]pyrrolo[2,1-*b*]quinazolin-6-one (2d)**, 42 mg, white solid, m.p.: over 280 °C. ^1^H NMR (500 MHz, DMSO-*d*_6_) *δ* 9.20 (s, 1H), 7.91 (s, 1H), 7.63 (s, 1H), 5.03 (s, 2H), 2.60 (s, 3H), 2.46 (s, 3H). ^13^C NMR (126 MHz, DMSO-*d*_6_) *δ* 163.4, 159.8, 154.5, 144.5, 143.6, 137.3, 136.5, 135.9, 123.1, 120.7, 119.6, 43.2, 20.9, 17.3. HRMS (ESI): *m*/*z* [M+H]^+^ calcd for C_14_H_11_N_3_O_2_: 254.0924; found: 254.0924.

**9-Methoxy-4*H*,6*H*-isoxazolo[3′,4′:3,4]pyrrolo[2,1-*b*]quinazolin-6-one (2e)**, 37 mg, white solid, m.p.: over 280 °C. ^1^H NMR (500 MHz, DMSO-*d*_6_) *δ* 9.21 (s, 1H), 8.15 (d, *J* = 8.8 Hz, 1H), 7.34 (d, *J* = 2.5 Hz, 1H), 7.22 (dd, *J* = 8.9, 2.5 Hz, 1H), 5.01 (s, 2H), 3.94 (s, 3H). ^13^C NMR (126 MHz, DMSO-*d*_6_) *δ* 164.2, 163.4, 159.3, 154.7, 150.3, 145.8, 127.5, 119.8, 117.2, 114.3, 109.2, 55.9, 43.2. HRMS (ESI): *m*/*z* [M+H]^+^ calcd for C_13_H_9_N_3_O_3_: 256.0717; found:256.0714.

**9-(*p*-Tolyl)-4*H*,6*H*-isoxazolo[3′,4′:3,4]pyrrolo[2,1-*b*]quinazolin-6-one (2g)**, 47 mg, white solid, m.p.: 271–272 °C. ^1^H NMR (500 MHz, DMSO-*d*_6_) *δ* 9.22 (s, 1H), 8.29 (d, *J* = 8.3 Hz, 1H), 8.12 (d, *J* = 1.8 Hz, 1H), 7.94 (dd, *J* = 8.4, 1.9 Hz, 1H), 7.77 (d, *J* = 7.8 Hz, 2H), 7.35 (d, *J* = 7.8 Hz, 2H), 5.04 (s, 2H), 2.38 (s, 3H). ^13^C NMR (126 MHz, DMSO-*d*_6_) *δ* 163.4, 159.6, 154.7, 148.6, 146.0, 145.6, 138.4, 135.4, 129.8, 127.1, 126.6, 126.2, 124.9, 119.8, 119.4, 43.3, 20.7. HRMS (ESI): *m*/*z* [M+H]^+^ calcd for C_19_H_13_N_3_O_2_: 316.1081; found:316.1081.

**9-(4-Methoxyphenyl)-4*H*,6*H*-isoxazolo[3′,4′:3,4]pyrrolo[2,1-*b*]quinazolin-6-one (2h)**, 33 mg, yellow solid, m.p.: 252–253 °C. ^1^H NMR (500 MHz, DMSO-*d*_6_) 9.22 (d, *J* = 1.2 Hz, 1H), 8.27 (d, *J* = 8.3 Hz, 1H), 8.10 (d, *J* = 1.8 Hz, 1H), 7.94 (dd, *J* = 8.3, 1.9 Hz, 1H), 7.87–7.81 (m, 2H), 7.12–7.07 (m, 2H), 5.06–5.03 (m, 2H), 3.83. ^13^C NMR (126 MHz, DMSO-*d*_6_) *δ* 163.4, 160.0, 159.6, 154.7, 148.6, 145.7, 145.6, 130.5, 128.5, 126.6, 125.9, 124.4, 119.8, 119.1, 114.7, 55.3, 43.3. HRMS (ESI): *m*/*z* [M+H]^+^ calcd for C_19_H_13_N_3_O_3_: 332.1030; found:332.1030.

**9-Fluoro-4*H*,6*H*-isoxazolo[3′,4′:3,4]pyrrolo[2,1-*b*]quinazolin-6-one (2i)**, 36 mg, white solid, m.p.:277–278 °C. ^1^H NMR (500 MHz, DMSO-*d*_6_) *δ* 9.23 (d, *J* = 1.3 Hz, 1H), 8.30 (dd, *J* = 8.9, 6.2 Hz, 1H), 7.69 (dd, *J* = 10.0, 2.6 Hz, 1H), 7.51 (td, *J* = 8.7, 2.6 Hz, 1H), 5.02 (s, 2H). ^13^C NMR (126 MHz, DMSO-*d*_6_) *δ*165.7 (d, *J* = 251.5 Hz), 163.2, 159.1, 154.9, 150.3 (d, *J* = 13.4 Hz), 146.7, 129.0 (d, *J* = 11.0 Hz), 119.9, 117.9(d, *J* = 2.52 Hz), 116.4 (d, *J* = 23.3 Hz), 113.1 (d, *J* = 22.1 Hz), 43.5. HRMS (ESI): *m*/*z* [M+H]^+^ calcd for C_12_H_6_FN_3_O_2_: 244.0517; found: 244.0512.

**8,9-Difluoro-4*H*,6*H*-isoxazolo[3′,4′:3,4]pyrrolo[2,1-*b*]quinazolin-6-one (2j)**, 39 mg, white solid, m.p.:224–226 °C. ^1^H NMR (500 MHz, DMSO-*d*_6_) *δ* 9.24 (s, 1H), 8.19 (td, *J* = 9.3, 3.3 Hz, 1H), 8.02 (td, *J* = 8.7, 7.5, 3.0 Hz, 1H), 5.03 (s, 2H). ^13^C NMR (126 MHz, DMSO-*d*_6_) *δ* 163.2, 158.5, 155.0, 153.9 (dd, *J* = 251.5, 14.5 Hz), 149.2 (dd, *J* = 251.5, 16.1 Hz), 146.3, 146.0 (dd, *J* = 11.1, 2.6 Hz), 119.8, 118.4 (d, *J* = 7.2 Hz), 116.1 (d, *J* = 17.9 Hz), 113.7 (d, *J* = 18.8 Hz), 43.6. HRMS (ESI): *m*/*z* [M+H]^+^ calcd for C_12_H_5_F_2_N_3_O_2_: 262.0423; found: 262.0422.

**8-Chloro-4*H*,6*H*-isoxazolo[3′,4′:3,4]pyrrolo[2,1-*b*]quinazolin-6-one (2k)**, 39 mg, white solid, m.p.:248–250 °C, ^1^H NMR (500 MHz, DMSO-*d*_6_) *δ* 9.23 (s, 1H), 8.16 (d, *J* = 2.5 Hz, 1H), 7.94 (dd, *J* = 8.7, 2.5 Hz, 1H), 7.89 (d, *J* = 8.7 Hz, 1H), 5.02 (s, 2H). ^13^C NMR (126 MHz, DMSO-*d*_6_) *δ* 163.2, 158.7, 154.9, 146.7, 145.7, 134.8, 132.2, 130.1, 124.9, 122.1, 119.7, 43.5. HRMS (ESI): *m*/*z* [M+H]^+^ calcd for C_12_H_6_ClN_3_O_2_: 260.0221; found: 260.0220.

**8-Nitro-4*H*,6*H*-isoxazolo[3′,4′:3,4]pyrrolo[2,1-*b*]quinazolin-6-one (2l)**, 32 mg, yellow solid, m.p.: over 280 °C. ^1^H NMR (500 MHz, DMSO-*d*_6_) *δ* 9.28 (s, 1H), 8.90 (d, *J* = 2.7 Hz, 1H), 8.64 (dd, *J* = 8.9, 2.7 Hz, 1H), 8.08 (d, *J* = 8.9 Hz, 1H), 5.08 (s, 2H). ^13^C NMR (126 MHz, DMSO-*d*_6_) *δ* 163.1, 159.0, 155.3, 152.2, 148.4, 145.7, 129.7, 128.7, 121.9, 121.1, 120.0, 43.9. HRMS (ESI): *m*/*z* [M+H]^+^ calcd for C_12_H_6_N_4_O_4_: 271.0462; found: 271.0641.

**4*H*,6*H*-Isoxazolo[3′,4′:3,4]pyrrolo[1,2-*a*]pyrido[2,3-*d*]pyrimidin-6-one (2m)**, 21mg, white solid, m.p.: over 280 °C. ^1^H NMR (500 MHz, DMSO-*d*_6_) *δ* 9.27 (d, *J* = 1.6 Hz, 1H), 9.07 (dd, *J* = 4.5, 2.0 Hz, 1H), 8.66 (dd, *J* = 7.9, 2.1 Hz, 1H), 7.68 (dd, *J* = 7.9, 4.6 Hz, 1H), 5.05 (s, 2H). ^13^C NMR (126 MHz, DMSO-*d*_6_) *δ* 163.3, 160.2, 158.1, 156.1, 155.0, 148.3, 135.7, 123.2, 119.8, 116.3, 43.6. HRMS (ESI): *m*/*z* [M+H]^+^ calcd for C_11_H_6_N_4_O_2_: 227.0564; found:227.0560.

**4*H*-Isoxazolo[3′,4′:4,5]pyrano[3,2-*b*]quinoline (4a)**, 36 mg, brown solid, m.p.: 194–196 °C. ^1^H NMR (500 MHz, CDCl_3_) *δ* 8.40 (s, 1H), 8.21 (d, *J* = 8.5 Hz, 1H), 7.74–7.68 (m, 2H), 7.62 (dd, *J* = 8.5, 6.7 Hz, 1H), 7.53 (t, *J* = 7.5 Hz, 1H), 5.35 (s, 2H). ^13^C NMR (126 MHz, CDCl_3_) *δ* 154.1, 152.0, 148.9, 144.6, 137.0, 130.2, 130.0, 128.2, 128.1, 126.7, 120.9, 113.8, 61.5. HRMS (ESI): *m*/*z* [M+H]^+^ calcd for C_13_H_8_N_2_O_2_: 225.0659; found: 225.0658.

**8-Chloro-4*H*-isoxazolo[3′,4′:4,5]pyrano[3,2-*b*]quinoline (4b)**, 39 mg, White solid, m.p.: 273–274 °C. ^1^H NMR (500 MHz, CDCl_3_) *δ* 8.42 (s, 1H), 8.15 (d, *J* = 9.0 Hz, 1H), 7.72 (d, *J* = 2.5 Hz, 1H), 7.62 (s, 1H), 7.55 (dd, *J* = 9.1, 2.3 Hz, 1H), 5.38 (s, 2H). ^13^C NMR (126 MHz, CDCl_3_) *δ* 153.8, 152.2, 149.5, 142.9, 137.2, 134.1, 131.6, 130.8, 129.3, 125.3, 120.0, 113.6, 61.7. HRMS (ESI): *m*/*z* [M+H]^+^ calcd for C_13_H_7_ClN_2_O_2_: 259.0269; found: 259.0269.

**4*H***-[1,3]**Dioxolo[4,5-*g*]isoxazolo[3′,4′:4,5]pyrano[3,2-*b*]quinoline (4c)**, 13 mg, brown solid, m.p.: over 280 °C. ^1^H NMR (500 MHz, CDCl_3_) *δ* 8.35 (d, *J* = 1.3 Hz, 1H), 7.55 (s, 1H), 7.48 (s, 1H), 6.96 (s, 1H), 6.11 (s, 2H), 5.32–5.29 (m, 2H). ^13^C NMR (126 MHz, CDCl_3_) δ 154.2, 151.6, 150.1, 149.4, 148.4, 142.7, 134.1, 128.0, 120.8, 113.4, 106.1, 101.9, 101.7, 61.5. HRMS (ESI): *m*/*z* [M+H]^+^ calcd for C_14_H_8_N_2_O_4_: 269.0557; found: 269.0556.

**Methyl 4*H*-isoxazolo[3′,4′:4,5]pyrano[3,2-*b*]quinoline-6-carboxylate (4d)**, 38 mg, yellow solid, m.p.: 252–254 °C. ^1^H NMR (500 MHz, CDCl_3_) *δ* 8.45 (s, 1H), 8.25 (d, *J* = 7.7 Hz, 1H), 7.75 (dd, *J* = 8.4, 1.5 Hz, 1H), 7.67 (ddd, *J* = 8.5, 6.9, 1.5 Hz, 1H), 7.60 (ddd, *J* = 8.3, 6.9, 1.4 Hz, 1H), 5.43 (d, *J* = 1.3 Hz, 2H), 4.08 (s, 3H). ^13^C NMR (126 MHz, CDCl_3_) *δ* 165.6, 153.6, 152.4, 145.2, 144.1, 137.0, 130. 6, 129.2, 128.6, 126.1, 124.8, 124.0, 113.4, 62.2, 53.0. HRMS (ESI): *m*/*z* [M+H]^+^ calcd for C_15_H_10_N_2_O_4_: 283.0713; found: 283.0713.

**Ethyl 4*H*-isoxazolo[3′,4′:4,5]pyrano[3,2-*b*]quinoline-6-carboxylate (4e)**, 44 mg, brown solid. m.p.: 197–198 °C. ^1^H NMR (500 MHz, CDCl_3_) *δ* 8.44 (s, 1H), 8.24 (d, *J* = 7.8 Hz, 1H), 7.75 (dd, *J* = 8.4, 1.4 Hz, 1H), 7.66 (ddd, *J* = 8.4, 6.9, 1.5 Hz, 1H), 7.59 (ddd, *J* = 8.3, 6.9, 1.3 Hz, 1H), 5.42 (d, *J* = 1.3 Hz, 2H), 4.56 (q, *J* = 7.1 Hz, 2H), 1.46 (t, *J* = 7.2 Hz, 3H). ^13^C NMR (126 MHz, CDCl_3_) *δ* 165.2, 153.6, 152.3, 145.1, 144.1, 137.0, 130.5, 129.1, 128.6, 126.1, 125.2, 123.9, 113.4, 62.2, 62.1, 14.3. HRMS (ESI): *m*/*z* [M+H]^+^ calcd for C_16_H_12_N_2_O_4_: 297.0870; found: 297.0869.

**4-Methoxybenzyl 4*H*-isoxazolo[3′,4′:4,5]pyrano[3,2-*b*]quinoline-6-carboxylate (4f)**, 44 mg, yellow solid, m.p.: 120–122 °C. ^1^H NMR (500 MHz, CDCl_3_) *δ* 8.42 (s, 1H), 8.22 (d, *J* = 8.4 Hz, 2H), 7.68–7.60 (m, 2H), 7.57–7.50 (m, 1H), 7.43 (d, *J* = 8.7 Hz, 3H), 6.93 (d, *J* = 8.7 Hz, 3H), 5.46 (s, 4H), 5.38 (s, 2H), 3.82 (s, 5H). ^13^C NMR (126 MHz, CDCl_3_) *δ* 165.1, 159.9, 153.5, 152.3, 145.2, 144.1, 136.9, 130.5, 130.4, 129.1, 128.5, 127.3, 126.1, 124.8, 123.8, 114.0, 113.4, 67.7, 62.0, 55.3. HRMS (ESI): *m*/*z* [M+H]^+^ calcd for C_22_H_16_N_2_O_5_:389.1132; found:389.1131.

**2-Bromobenzyl 4*H*-isoxazolo[3′,4′:4,5]pyrano[3,2-*b*]quinoline-6-carboxylate (4g)**, 50 mg, yellow solid, m.p.: 166–168 °C. ^1^H NMR (500 MHz, CDCl_3_) *δ* 8.44 (s, 1H), 8.24 (d, *J* = 8.5 Hz, 1H), 7.81 (d, *J* = 8.4 Hz, 1H), 7.69–7.54 (m, 4H), 7.34 (t, *J* = 7.5 Hz, 1H), 7.23 (td, *J* = 7.8, 1.7 Hz, 1H), 5.62 (s, 2H), 5.41 (s, 2H). ^13^C NMR (126 MHz, CDCl_3_) *δ* 164.9, 153.5, 152.4, 145.4, 144.1, 137.0, 134.4, 133.0, 130.5, 130.4, 130.2, 129.2, 128.6, 127.6, 126.1, 124.4, 124.00, 123.7, 113.4, 67.4, 62.1. HRMS (ESI): *m*/*z* [M+H]^+^ calcd for C_21_H_13_BrN_2_O_4_:437.0131; found:437.0133.

**Benzyl 4*H*-isoxazolo[3′,4′:4,5]pyrano[3,2-*b*]quinoline-6-carboxylate (4h)**, 56 mg, white solid, m.p.: 126–128 °C. ^1^H NMR (500 MHz, CDCl_3_) *δ* 8.43 (d, *J* = 1.5 Hz, 1H), 8.23 (d, *J* = 8.4 Hz, 1H), 7.69 (d, *J* = 8.5 Hz, 1H), 7.65 (t, *J* = 7.7 Hz, 1H), 7.58–7.52 (m, 1H), 7.49 (d, *J* = 6.8 Hz, 2H), 7.40 (dt, *J* = 12.3, 6.8 Hz, 3H), 5.54 (s, 2H), 5.39 (s, 2H). ^13^C NMR (126 MHz, CDCl_3_) *δ* 165.1, 153.5, 152.3, 145.2, 144.1, 137.00, 135.1, 130.5, 129.2, 128.7, 128.6, 128.6, 128.5, 126.1, 124.7, 123.9, 113.4, 67.8, 62.1. HRMS (ESI): *m*/*z* [M+H]^+^ calcd for: C_21_H_14_N_2_O_4_: 359.1026; found:359.1025.

**N,N-Dibenzyl-4*H*-isoxazolo[3′,4′:4,5]pyrano[3,2-*b*]quinoline-6-carboxamide (4i)**, 45 mg, yellow solid, m.p.: 89–91 °C. ^1^H NMR (500 MHz, CDCl_3_) *δ* 8.46 (s, 1H), 8.25 (d, *J* = 8.4 Hz, 1H), 7.74 (d, *J* = 8.7 Hz, 1H), 7.68 (t, *J* = 7.6 Hz, 1H), 7.60 (t, *J* = 7.8 Hz, 1H), 7.52–7.42 (m, 4H), 7.39 (t, *J* = 6.8 Hz, 1H), 7.32–7.21 (m, 4H), 7.07 (d, *J* = 6.4 Hz, 2H), 5.48 (d, *J* = 13.7 Hz, 1H), 5.25 (d, *J* = 13.6 Hz, 1H), 4.98 (d, *J* = 14.5 Hz, 1H), 4.79 (d, *J* = 14.5 Hz, 1H), 4.32–4.21 (m, 2H). ^13^C NMR (126 MHz, CDCl_3_) *δ* 177.2, 166.0, 153.5, 152.4, 144.2, 143.9, 136.7, 136.3, 135.2, 130.5, 129.1, 128.7, 128.7, 128.6, 128.6, 127.8, 127.6, 127.5, 126.4, 123.8, 113.3, 61.9, 51.1, 46.7. HRMS (ESI): *m*/*z* [M+H]^+^ calcd for C_28_H_21_N_3_O_3_:448.1656; found:448.1656.

**4*H*-Isoxazolo[3′,4′:4,5]pyrano[3,2-*b*]quinolin-6-yl)(morpholino)methanone (4j)**, 33 mg, yellow solid, m.p.: over 280 °C. ^1^H NMR (500 MHz, CDCl_3_) *δ* 8.44 (s, 1H), 8.22 (d, *J* = 8.3 Hz, 1H), 7.68 (s, 1H), 7.66 (d, *J* = 8.9 Hz, 2H), 5.41 (q, *J* = 13.7 Hz, 2H), 4.03–3.91 (m, 2H), 3.84 (ddt, *J* = 18.4, 7.8, 5.7 Hz, 2H), 3.62 (ddd, *J* = 10.1, 6.2, 3.2 Hz, 1H), 3.53 (ddd, *J* = 11.2, 6.7, 3.2 Hz, 1H), 3.27 (ddd, *J* = 13.8, 6.7, 3.2 Hz, 1H), 3.19 (ddd, *J* = 13.6, 6.3, 3.3 Hz, 1H). ^13^C NMR (126 MHz, CDCl_3_) *δ* 163.9, 153.6, 152.4, 144.3, 143.9, 136.8, 130.6, 129.2, 128.8, 127.0, 126.2, 123.8, 113.4, 67.0, 66.8, 62.1, 47.0, 42.1. HRMS (ESI): *m*/*z* [M+H]^+^ calcd for C_18_H_15_N_3_O_4_: 338.1135; found: 338.1132.

**(1*S*,2*R*,5*S*)-2-Isopropyl-5-methylcyclohexyl4*H*-isoxazolo[3′,4′:4,5]pyrano[3,2-*b*]quinoline-6-carboxylate (4k)**, 18 mg, yellow solid, m.p.:176–178 °C. ^1^H NMR (500 MHz, CDCl_3_) *δ* 8.44 (s, 1H), 8.24 (d, *J* = 8.5 Hz, 1H), 7.73 (d, *J* = 8.4 Hz, 1H), 7.70–7.64 (m, 1H), 7.64–7.57 (m, 1H), 5.40 (q, *J* = 13.6 Hz, 2H), 5.13 (td, *J* = 11.0, 4.3 Hz, 1H), 2.34 (d, *J* = 12.1 Hz, 1H), 2.11 (pd, *J* = 6.9, 2.7 Hz, 1H), 1.78–1.72 (m, 2H), 1.63 (d, *J* = 2.9 Hz, 1H), 1.54–1.46 (m, 1H), 1.28–1.11 (m, 2H), 1.00 (d, *J* = 6.5 Hz, 3H), 0.92 (dd, *J* = 7.0, 4.0 Hz, 7H). ^13^C NMR (126 MHz, CDCl_3_) *δ* 165.0, 154.6, 152.3, 144.7, 144.2, 137.0, 130.5, 129.1, 128.6, 126.1, 125.8, 123.8, 113.4, 61.9, 46.9, 40.9, 34.1, 31.6, 26.0, 23.2, 22.0, 20.8, 16.0. HRMS (ESI): *m*/*z* [M+H]^+^ calcd for C_24_H_26_N_2_O_4_:407.1965; found: 407.1960.

**((3*aS*,4*S*,6*S*,6*aS*)-6-Methoxy-2,2-dimethyltetrahydrofuro[3,4-*d*][1,3]dioxol-4-yl)methyl4*H*-isoxazolo[3′,4′:4,5]pyrano[3,2-*b*]quinoline-6-carboxylate (4l)**, 31 mg, yellow solid, m.p.:139–141 °C. ^1^H NMR (500 MHz, CDCl_3_) *δ* 8.45 (s, 1H), 8.25 (d, *J* = 8.4 Hz, 2H), 7.82 (d, *J* = 8.3 Hz, 2H), 7.67 (dd, *J* = 8.4, 7.0 Hz, 2H), 7.64–7.57 (m, 1H), 5.43 (s, 3H), 5.04 (s, 2H), 4.77 (d, *J* = 5.9 Hz, 2H), 4.66 (d, *J* = 5.9 Hz, 2H), 4.58–4.52 (m, 3H), 4.51–4.45 (m, 2H), 3.38 (s, 5H), 1.50 (s, 5H), 1.33 (s, 5H). ^13^C NMR (126 MHz, CDCl_3_) *δ* 164.7, 153.5, 152.4, 145.4, 144.1, 137.0, 130.6, 129.3, 128.6, 126.0, 124.4, 123.9, 113.4, 112.7, 109.4, 85.1, 83.9, 81.8, 65.9, 62.1, 55.1, 26.4, 25.0. HRMS (ESI): *m*/*z* [M+H]^+^ calcd for C_23_H_22_N_2_O_8_:455.1449; found:455.1442.

**(7*S*,11*S*,*E*)-3,7,11,15-Tetramethylhexadec-2-en-1-yl 4*H*-isoxazolo[3′,4′:4,5]pyrano[3,2-*b*]quinoline-6-carboxylate (4m)**, 73 mg, orange oil, ^1^H NMR (500 MHz, CDCl_3_) *δ* 8.44 (d, *J* = 5.4 Hz, 1H), 8.23 (t, *J* = 7.3 Hz, 1H), 7.79 (dd, *J* = 37.8, 8.4 Hz, 1H), 7.65 (q, *J* = 7.6, 7.0 Hz, 1H), 7.58 (h, *J* = 8.2, 7.3 Hz, 1H), 5.57–5.48 (m, 1H), 5.41 (s, 2H), 5.00 (t, *J* = 7.6 Hz, 1H), 4.78–4.68 (m, 1H), 2.25–2.03 (m, 1H), 1.79 (d, *J* = 8.1 Hz, 2H), 1.51 (dtd, *J* = 13.2, 6.5, 2.3 Hz, 1H), 1.38–1.18 (m, 4H), 1.15–1.09 (m, 1H), 1.09–1.02 (m, 2H), 0.90–0.76 (m, 12H). ^13^C NMR (126 MHz, CDCl_3_) *δ* 165.2, 153.6, 152.3, 145.0, 144.6, 144.1, 136.9, 130.5, 129.0, 128.5, 126.1, 123.9, 117.9, 117.2, 113.4, 63.0, 62.1, 39.9, 39.3, 37.4, 37.3, 37.2, 36.6, 32.7, 32.6, 27.9, 25.6, 25.1, 24.7, 24.4, 22.7, 22.6, 19.7, 16.5. HRMS (ESI): *m*/*z* [M+H]^+^ calcd for C_34_H_46_N_2_O_4_:547.3530; found: 547.3528.

**(3*S*,8*S*,9*S*,10*R*,13*R*,14*S*,17*R*)-10,13-Dimethyl-17-((*R*)-6-methylheptan-2-yl)-2,3,4,7,8,9,10,11,12,13,14,15,16,17-tetradecahydro-1*H*-cyclopenta[a]phenanthren-3-yl 4*H*-isoxazolo[3′,4′:4,5]pyrano[3,2-*b*]quinoline-6-carboxylate (4n)**, 42 mg, yellow soild, m.p.: 196–197 °C. ^1^H NMR (500 MHz, CDCl_3_) *δ* 8.44 (s, 1H), 8.24 (d, *J* = 8.2 Hz, 1H), 7.81–7.74 (m, 1H), 7.70–7.64 (m, 1H), 7.63–7.58 (m, 1H), 5.49 (d, *J* = 5.4 Hz, 1H), 5.43 (d, *J* = 5.1 Hz, 2H), 5.09 (td, *J* = 11.5, 5.6 Hz, 1H), 2.64–2.46 (m, 2H), 2.18–1.92 (m, 2H), 1.85 (ddd, *J* = 9.8, 5.8, 3.8 Hz, 1H), 1.62–1.59 (m, 5H), 1.50 (qd, *J* = 11.4, 9.1, 3.8 Hz, 2H), 1.42–1.33 (m, 2H), 1.25 (ddt, *J* = 19.4, 13.3, 6.0 Hz, 2H), 1.20–1.07 (m, 3H), 1.05 (s, 4H), 0.93 (d, *J* = 6.3 Hz, 3H), 0.87 (dd, *J* = 6.6, 2.3 Hz, 8H), 0.70 (d, *J* = 8.2 Hz, 3H). ^13^C NMR (126 MHz, CDCl_3_) *δ* 164.6, 153.6, 152.3, 144.9, 144.1, 139.2, 137.0, 130.5, 129.1, 128.6, 126.1, 125.5, 123.9, 123.3, 113.5, 76.3, 62.1, 56.7, 56.1, 50.0, 42.3, 39.7, 39.5, 38.1, 37.0, 36.6, 36.2, 35.8, 31.9, 31.8, 28.2, 28.0, 27.9, 24.3, 23.8, 22.8, 22.5, 21.0, 19.3, 18.7, 11.9. HRMS (ESI): *m*/*z* [M+H]^+^ calcd for C_41_H_52_N_2_O_4_:637.4000; found: 637.4000.

**(3*S*,8*S*,9*S*,10*R*,13*R*,14*S*,17*R*)-17-((2*R*,5*S*,*E*)-5-Ethyl-6-methylhept-3-en-2-yl)-10,13-dimethyl-2,3,4,7,8,9,10,11,12,13,14,15,16,17-tetradecahydro-1*H*-cyclopenta[a]phenanthren-3-yl 4*H*-isoxazolo[3′,4′:4,5]pyrano[3,2-*b*]quinoline-6-carboxylate (4o)**, 38 mg, yellow solid, m.p.: 213–214 °C. ^1^H NMR (500 MHz, CDCl_3_) *δ* 8.44 (s, 1H), 8.24 (d, *J* = 8.4 Hz, 1H), 7.77 (dd, *J* = 8.4, 1.4 Hz, 1H), 7.70–7.63 (m, 1H), 7.63–7.57 (m, 1H), 5.49 (d, *J* = 5.5 Hz, 1H), 5.43 (s, 2H), 5.27–5.09 (m, 1H), 5.07–4.99 (m, 1H), 2.63–2.46 (m, 2H), 2.18–1.93 (m, 3H), 1.81–1.66 (m, 2H), 1.60–1.45 (m, 3H), 1.29–1.14 (m, 2H), 1.04 (d, *J* = 13.4 Hz, 5H), 0.93 (d, *J* = 6.2 Hz, 1H), 0.88–0.78 (m, 7H), 0.70 (d, *J* = 9.0 Hz, 3H). ^13^C NMR (126 MHz, CDCl_3_) *δ* 164.6, 153.6, 152.3, 144.9, 144.1, 139.2, 138.3, 137.0, 130.5, 129.3, 129.1, 128.5, 126.0, 125.5, 123.8, 123.3, 113.4, 76.3, 62.1, 56.8, 55.9, 51.2, 50.0, 45.8, 42.2, 40.5, 39.7, 39.6, 38.1, 37.0, 36.6, 31.9, 31.9, 31.8, 28.9, 27.9, 25.4, 24.3, 21.2, 21.1, 21.0, 19.3, 19.0, 19.0, 12.2, 12.0. HRMS (ESI): *m*/*z* [M+H]^+^ calcd for C_43_H_54_N_2_O_4_:663.4156; found: 663.4150.

**3-Phenyl-2-(5-phenylisoxazol-3-yl)quinazolin-4(3*H*)-one (6a)**, 41 mg, yellow soild, m.p.: 120–122 °C. ^1^H NMR (500 MHz, CDCl_3_) *δ* 8.39 (d, *J* = 8.0 Hz, 1H), 7.92–7.83 (m, 4H), 7.69–7.64 (m, 3H), 7.64–7.58 (m, 1H), 7.52–7.40 (m, 9H), 7.32 (dd, *J* = 8.0, 1.7 Hz, 3H), 6.51 (s, 2H). ^13^C NMR (126 MHz, CDCl_3_) *δ* 170.1, 161.8, 159.3, 147.1, 145.6, 137.0, 134.9, 130.6, 129.4, 129.0, 128.8, 128.3, 128.2, 127.3, 126.6, 125.9, 121.6. HRMS (ESI): *m*/*z* [M+H]^+^ calcd for C_23_H_15_N_3_O_2_:366.1237; found: 366.1228.

## 4. Conclusions

In summary, a facile and practical 1,3-dipolar cycloaddition reaction that accessed a wide variety of isoxazole-fused tricyclic quinazoline alkaloids and their derivatives has been developed under metal-free conditions. In this system, methyl azaarenes were transformed into nitrile oxides in situ by using TBN as the radical initiator and source of N-O without transition metal. This strategy has broad substrate applicability and good functional group tolerance with facile manipulation of readily available starting materials. Natural product modifications confirmed the practical utility of this synthetic method.

## Figures and Tables

**Table 1:** Optimization of reaction conditions ^a^.

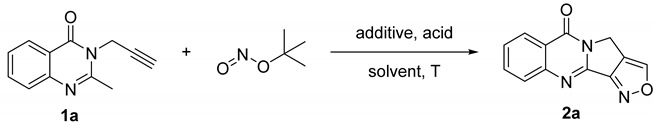					
Entry	Solvent	Acid	Additive	Temp (°C)	Yield ^b^ (%)
1	DMSO			80	45
2	DMF			80	62
3	1,4-Dioxane			80	56
4	MeCN			80	64
5	MeCN			60	55
6	MeCN			100	65
7 ^c^	MeCN			100	72
8 ^c,d^	MeCN			100	75
9 ^c,d^	MeCN		NCS	100	76
10 ^c,d^	MeCN	TFA	NCS	100	46
11 ^c,d^	MeCN	AcOH	NCS	100	80
12 ^c,d^	MeCN	Propanoic acid	NCS	100	68
13 ^c,d^	MeCN	Propanedioic acid	NCS	100	58
14 ^c,d^	MeCN	4-Nitrobenzoic acid	NCS	100	68
15 ^c,d^	MeCN	2-Nitrobenzoic acid	NCS	100	73
16 ^c,d^	MeCN	4-Chlorobenzoic acid	NCS	100	71
17 ^c,d^	MeCN	PTA ^e^	NCS	100	55
18 ^c,d^	MeCN	AcOH		100	66
19 ^c,d^	MeCN	4-Chlorobenzoic acid		100	42

^a^ Reaction conditions: **1a** (0.2 mmol), TBN (1.0 mmol), NCS (0.1 mmol), AcOH (0.1 mmol) were stirred in acetonitrile (2 mL) at 100 °C for 6 h under air. ^b^ Isolated yields. ^c^ TBN (5.5 equiv.). ^d^ under argon. ^e^ p-Toluic acid.

## Data Availability

Not applicable.

## Supplementary Materials

The following supporting information can be downloaded at: https://www.mdpi.com/article/10.3390/molecules28062787/s1, Characterization data for product **2**, **4** and **6a**, include ^1^H-NMR, ^13^C-NMR and high-resolution mass spectrometry (HRMS) spectroscopies are available online.

## Sample Availability

Samples of the compounds **2** and **4** are available from the authors.
