# Supplementary material for: One-Pot Synthesis of Isoxazole-Fused Tricyclic Quinazoline Alkaloid Derivatives via Intramolecular Cycloaddition of Propargyl-Substituted Methyl Azaarenes under Metal-Free Conditions
Source: Molecules. 2023 Mar 20;28(6):2787. doi: 10.3390/molecules28062787 (PMC10057414; doi:10.3390/molecules28062787)
Supplement: Supplementary file 1 [file molecules-28-02787-s001.zip › molecules-2250270-supplementary.pdf]

Supporting Information

One-Pot Synthesis of Isoxazole-Fused Tricyclic Quinazoline Alkaloid Derivatives via Intramolecular Cycloaddition of Methyl Azaarenes under Metal-free Conditions

Zhuo Wang [1], Yu-Han Zhao [1], Jia-Xin Chen [1], Meng-Yao Chen [1], Xue-Han Li [1], Ting Jiang [1], Fang Liu [1], Xi Yang [1], Yuan-Yuan Sun[1] and Yan-Ping Zhu [1,2 *]

[1] Key Laboratory of Molecular Pharmacology and Drug Evaluation, Ministry of Education, Collaborative Innovation Center of Advanced Drug Delivery System and Biotech Drugs in Universities of Shandong, School of Pharmacy, Yantai University, Shandong, Yantai, 264005, China.

[2] Anhui Laboratory of Molecule-Based Materials, College of Chemistry and Materials Science, Anhui Normal University, Wuhu 241000, China

E-mail: chemzyp@foxmail.com; chemzyp@ytu.edu.cn,

Table of Contents

1. Evidence in support of the hypothetic mechanism 3

2. Copy of ^{1}H、^{13}C NMR and HR-MS Spectra of Products............................... 4

1. Evidence in support of the hypothetic mechanism

To gain further insight into the reaction mechanism, some control experiments were performed as illustrated in Scheme S1. To address the possible reaction intermediate, 2-methyl-3-phenylquinazolinone (**5a**) was reacted under the standard conditions for 20 mins, nitrile oxides (**5ac**) could be detected by MS (APCI).

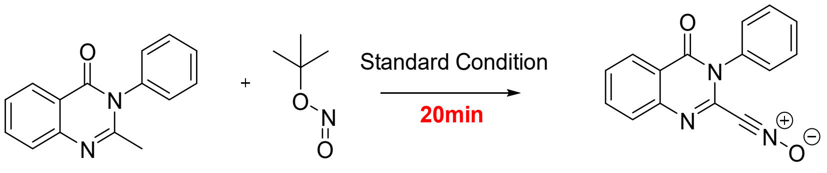

Scheme S1. Control experiment of **5a** with TBN.

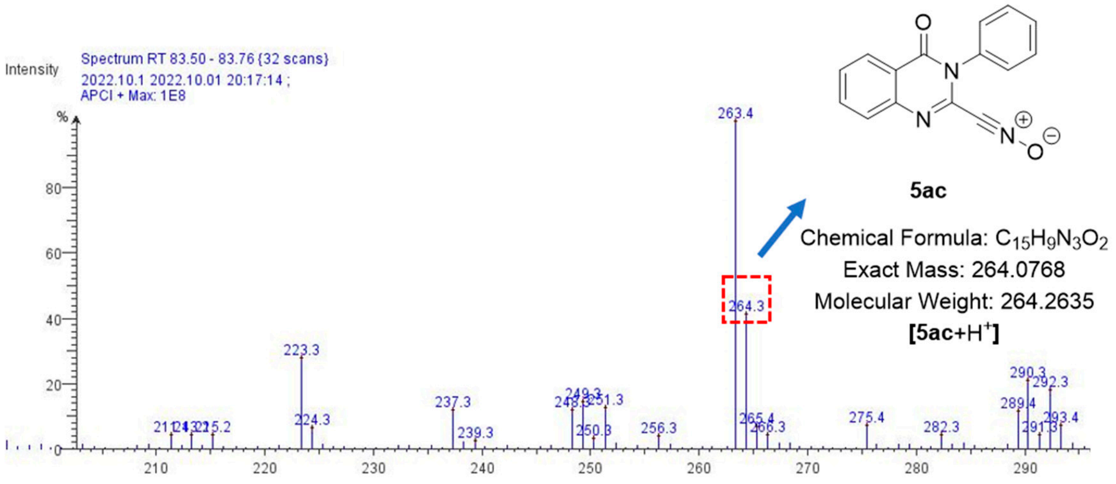

Scheme S2. The MS(APCI) results for the reaction of **5a** was reacted under the standard conditions for 20 mins.

2. Copy of ¹H、¹³C NMR and HR-MS Spectra of Products

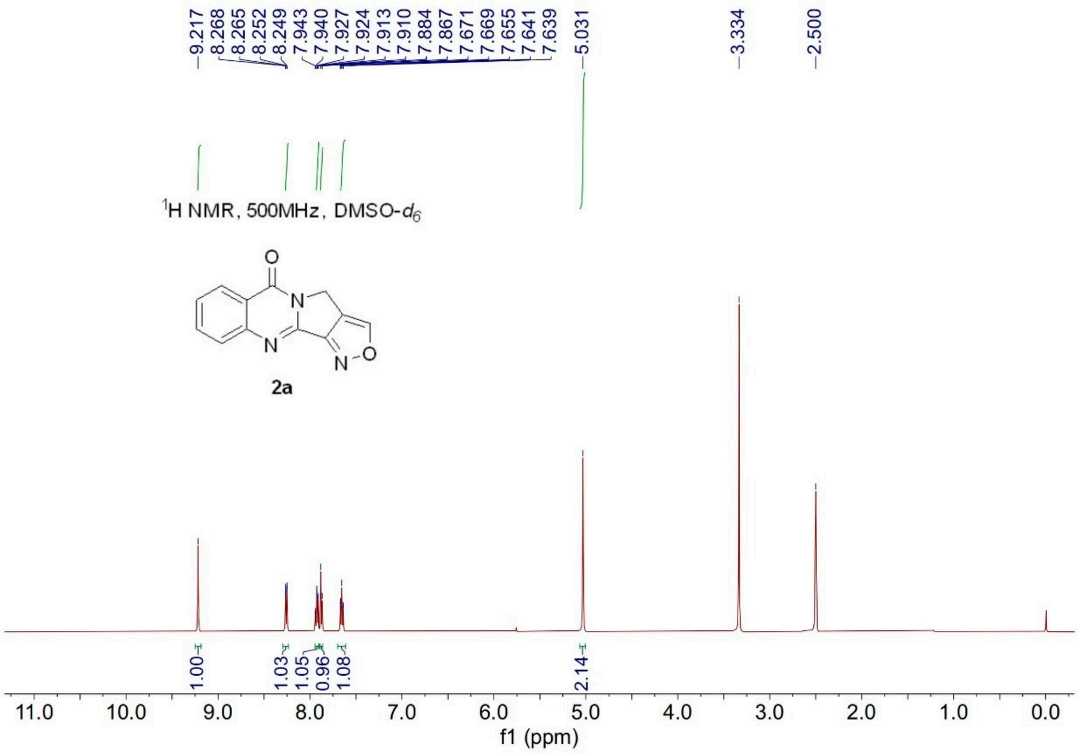

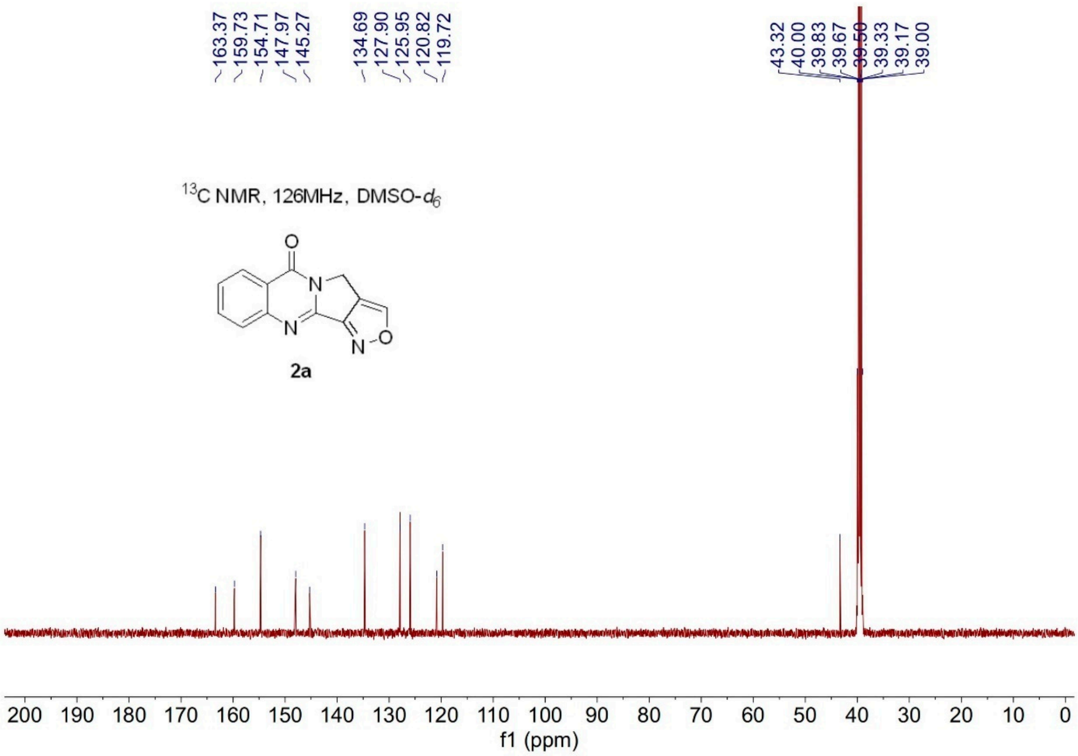

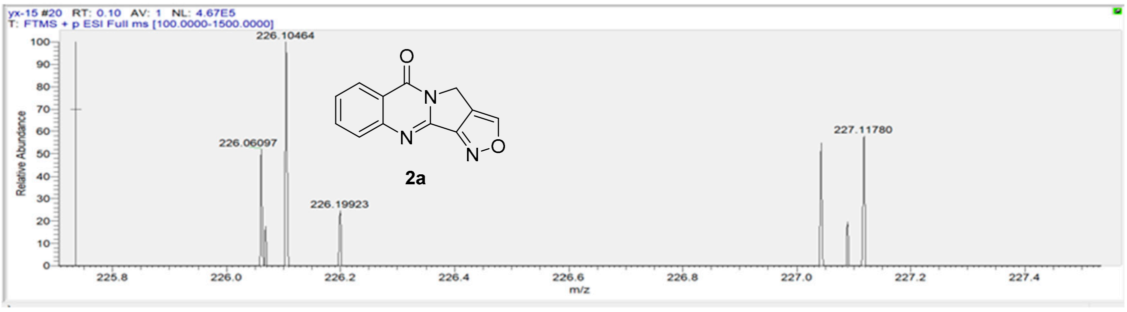
yx-15 #20 RT: 0.10 AV: 1 NL: 4.67E5
T: FTMS + p ESI Full ms [100.0000-1500.0000]
226.10464
226.06097
226.19923
227.11780
Relative Abundance
m/z
2a

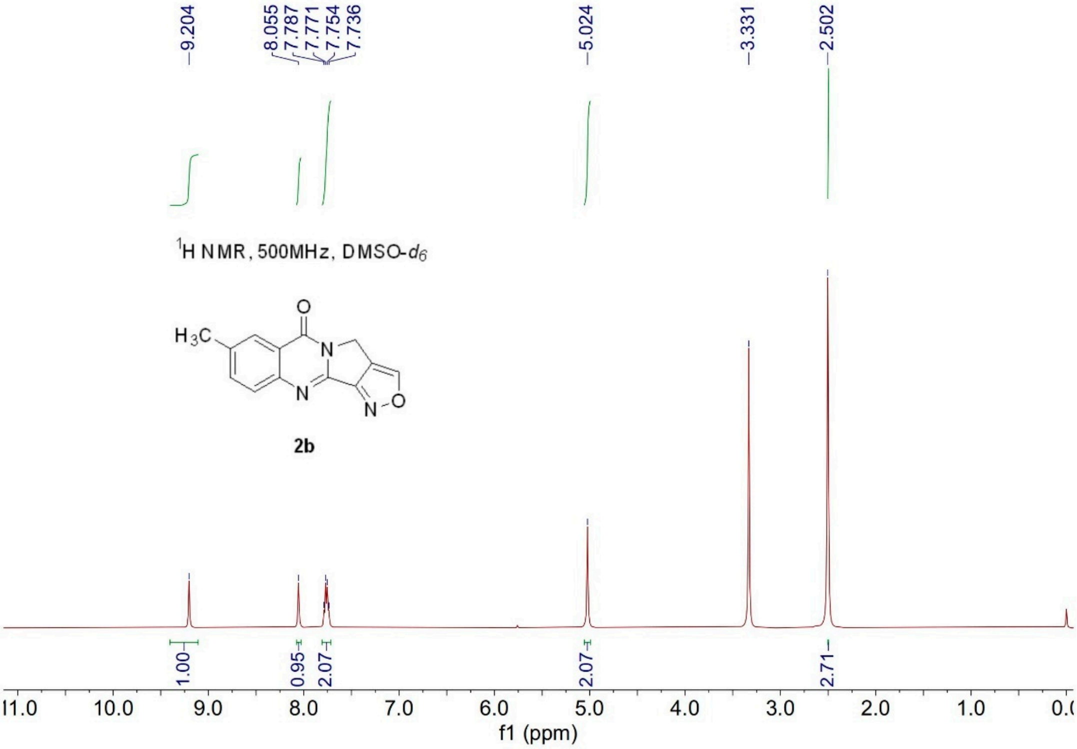
9.204
8.055
7.787
7.771
7.754
7.736
5.024
3.331
2.502
1H NMR, 500MHz, DMSO-d6
H3C
2b
1.00
0.95
2.07
2.07
2.71
f1 (ppm)

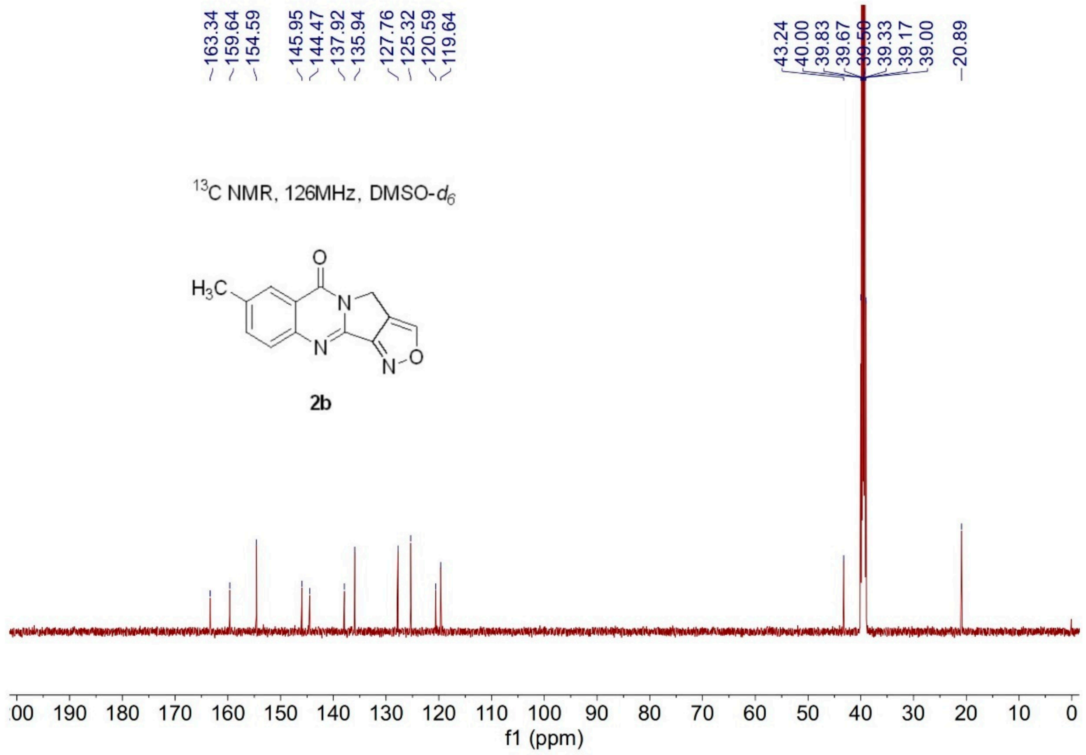
163.34
159.64
154.59
145.95
144.47
137.92
135.94
127.76
125.32
120.59
119.64
43.24
40.00
39.83
39.67
39.50
39.33
39.17
39.00
20.89
^{13}C NMR, 126MHz, DMSO-d_6
H_3C
O
N
N
N
O
2b
00 190 180 170 160 150 140 130 120 110 100 90 80 70 60 50 40 30 20 10 0
f1 (ppm)

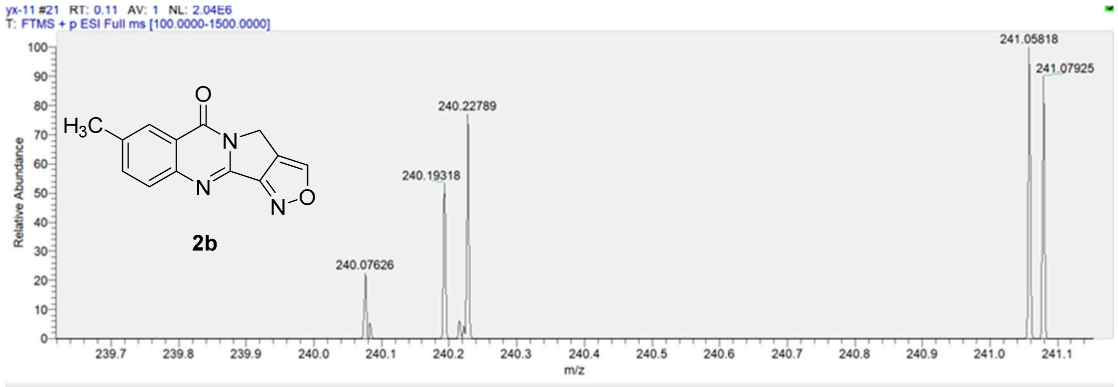
yx-11 #21 RT: 0.11 AV: 1 NL: 2.04E6
T: FTMS + p ESI Full ms [100.0000-1500.0000]
241.05818
241.07925
240.22789
240.19318
240.07626
Relative Abundance
H_3C
O
N
N
N
O
2b
239.7 239.8 239.9 240.0 240.1 240.2 240.3 240.4 240.5 240.6 240.7 240.8 240.9 241.0 241.1
m/z

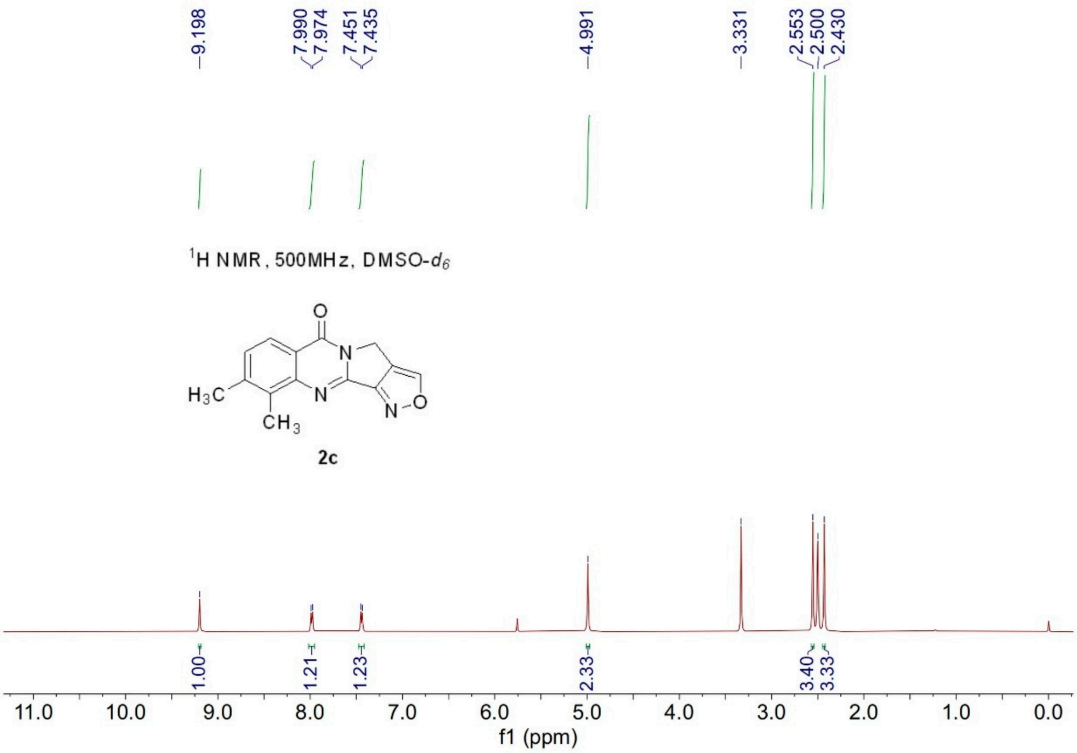
9.198
7.990
7.974
7.451
7.435
4.991
3.331
2.553
2.500
2.430
1H NMR, 500MHz, DMSO-d6
H3C
CH3
N
N
O
N
O
2c
1.00
1.21
1.23
2.33
3.40
3.33
11.0 10.0 9.0 8.0 7.0 6.0 5.0 4.0 3.0 2.0 1.0 0.0
f1 (ppm)

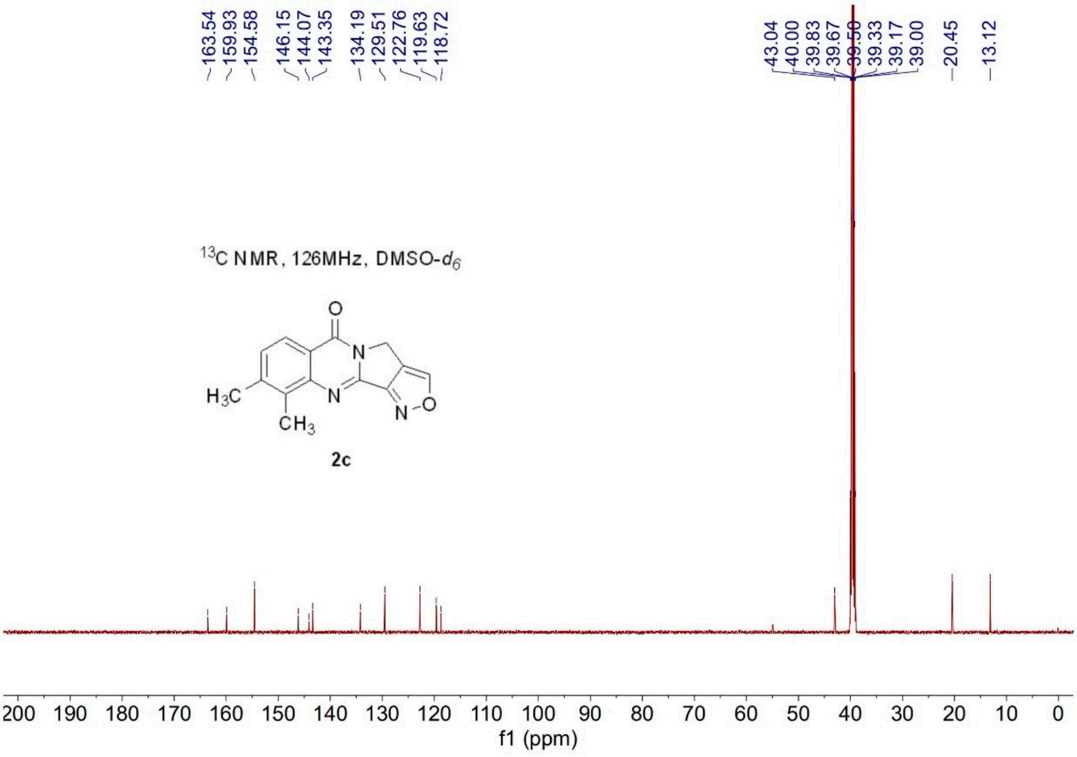
163.54
159.93
154.58
146.15
144.07
143.35
134.19
129.51
122.76
119.63
118.72
43.04
40.00
39.83
39.67
39.50
39.33
39.17
39.00
20.45
13.12
13C NMR, 126MHz, DMSO-d6
H3C
CH3
N
N
O
N
O
2c
200 190 180 170 160 150 140 130 120 110 100 90 80 70 60 50 40 30 20 10 0
f1 (ppm)

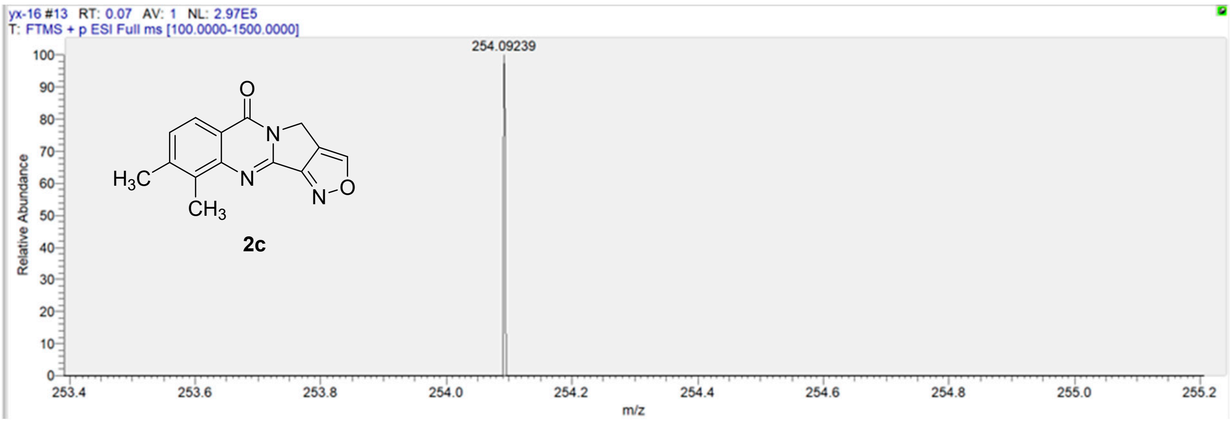
yx-16 #13 RT: 0.07 AV: 1 NL: 2.97E5
T: FTMS + p ESI Full ms [100.0000-1500.0000]
254.09239
Relative Abundance
m/z
2c

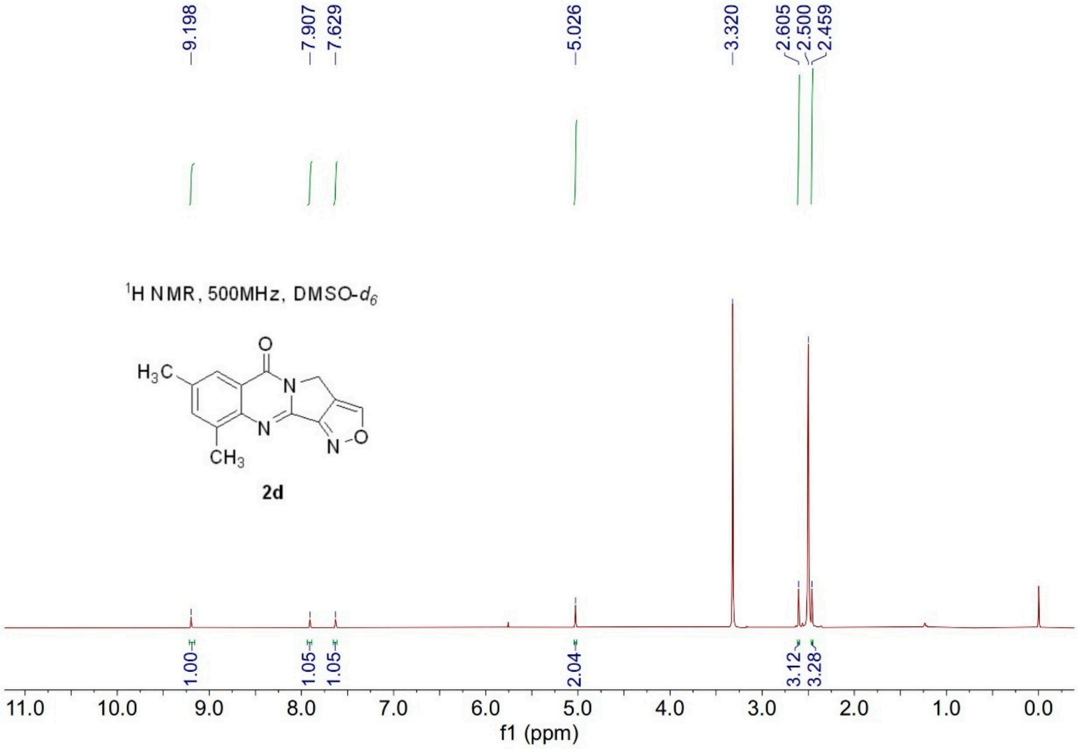
9.198
7.907
7.629
5.026
3.320
2.605
2.500
2.459
1H NMR, 500MHz, DMSO-d6
2d
1.00
1.05
1.05
2.04
3.12
3.28
f1 (ppm)

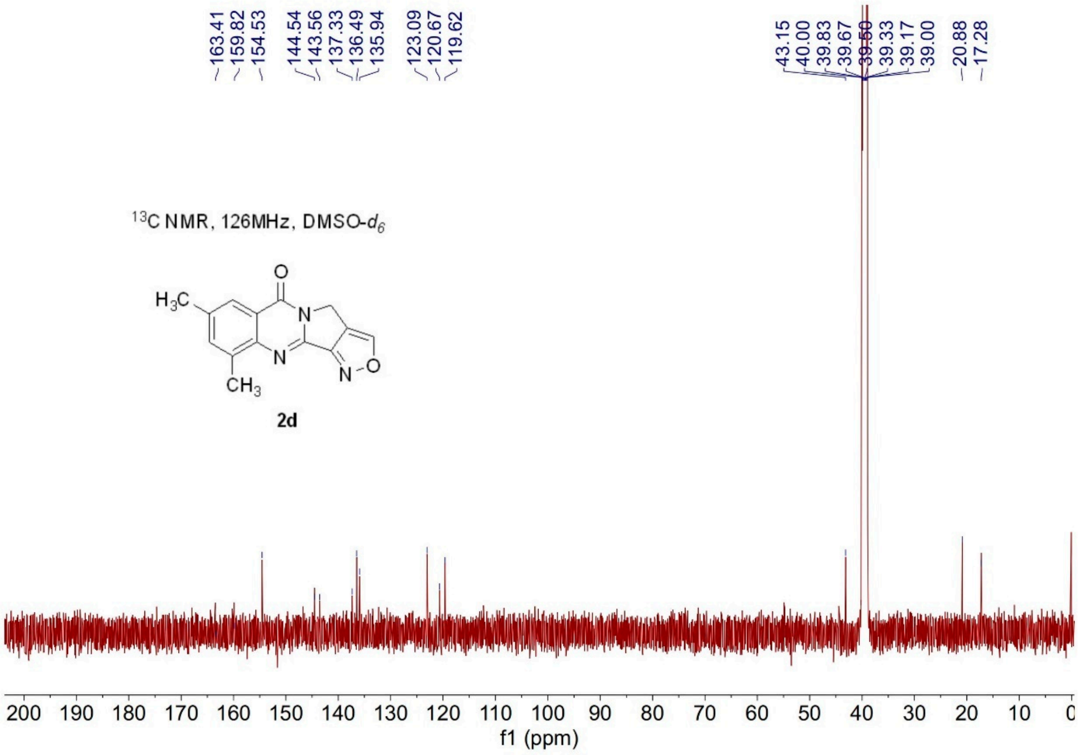
163.41
159.82
154.53
144.54
143.56
137.33
136.49
135.94
123.09
120.67
119.62
43.15
40.00
39.83
39.67
39.50
39.33
39.17
39.00
20.88
17.28
^{13}C NMR, 126MHz, DMSO-d_6
2d
f1 (ppm)

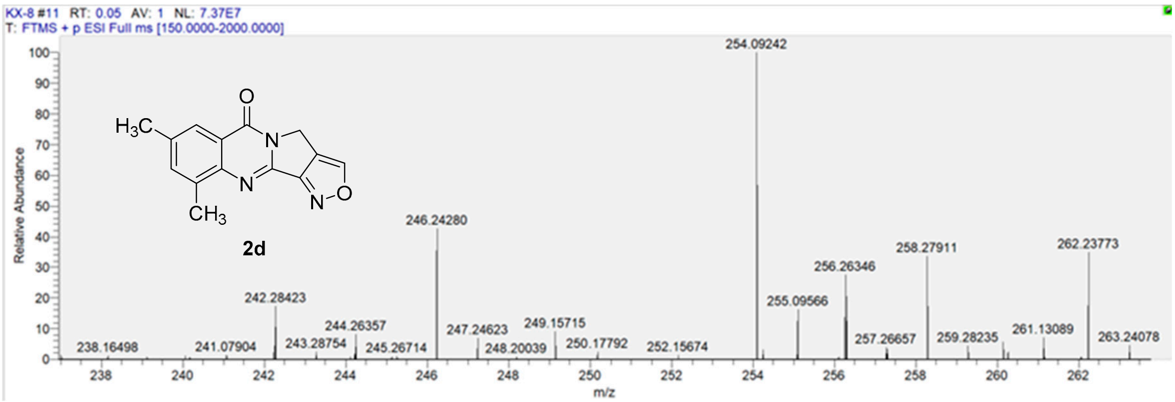
KX-8 #11 RT: 0.05 AV: 1 NL: 7.37E7
T: FTMS + p ESI Full ms [150.0000-2000.0000]
254.09242
2d
246.24280
262.23773
258.27911
256.26346
255.09566
242.28423
244.26357
249.15715
261.13089
247.24623
Relative Abundance
m/z

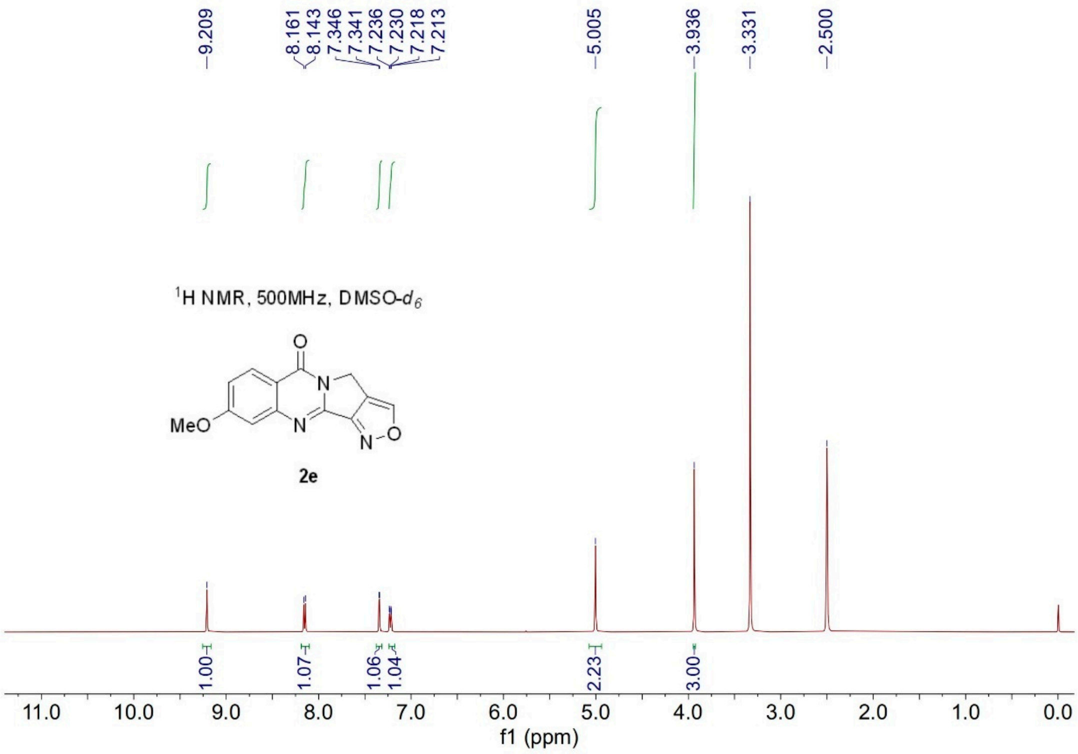
9.209
8.161
8.143
7.346
7.341
7.236
7.230
7.218
7.213
5.005
3.936
3.331
2.500
1H NMR, 500MHz, DMSO-d6
MeO
O
N
N
N
O
2e
1.00
1.07
1.06
1.04
2.23
3.00
11.0 10.0 9.0 8.0 7.0 6.0 5.0 4.0 3.0 2.0 1.0 0.0
f1 (ppm)

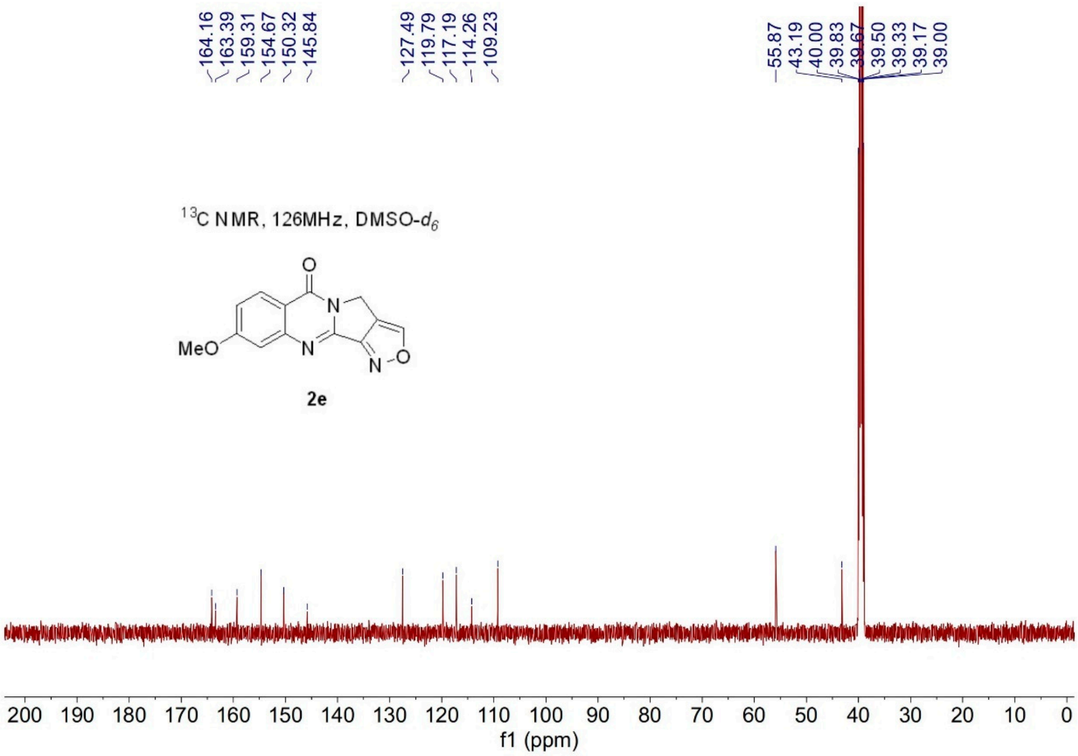
164.16
163.39
159.31
154.67
150.32
145.84
127.49
119.79
117.19
114.26
109.23
55.87
43.19
40.00
39.83
39.67
39.50
39.33
39.17
39.00
13C NMR, 126MHz, DMSO-d6
MeO
O
N
N
N
O
2e
200 190 180 170 160 150 140 130 120 110 100 90 80 70 60 50 40 30 20 10 0
f1 (ppm)

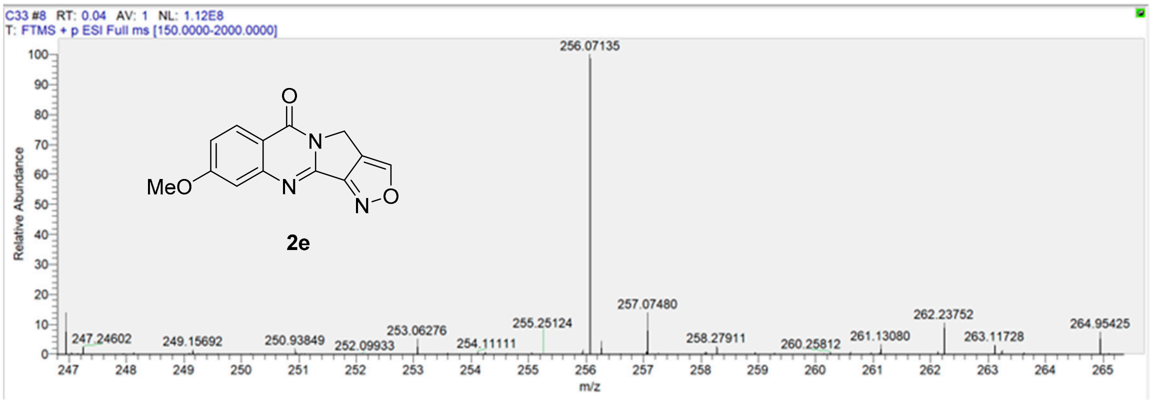
C33 #8 RT: 0.04 AV: 1 NL: 1.12E8
T: FTMS + p ESI Full ms [150.0000-2000.0000]
256.07135
257.07480
262.23752
264.95425
263.11728
261.13080
260.25812
258.27911
255.25124
253.06276
254.11111
252.09933
250.93849
249.15692
247.24602
Relative Abundance
m/z
MeO
N
O
N
O
2e

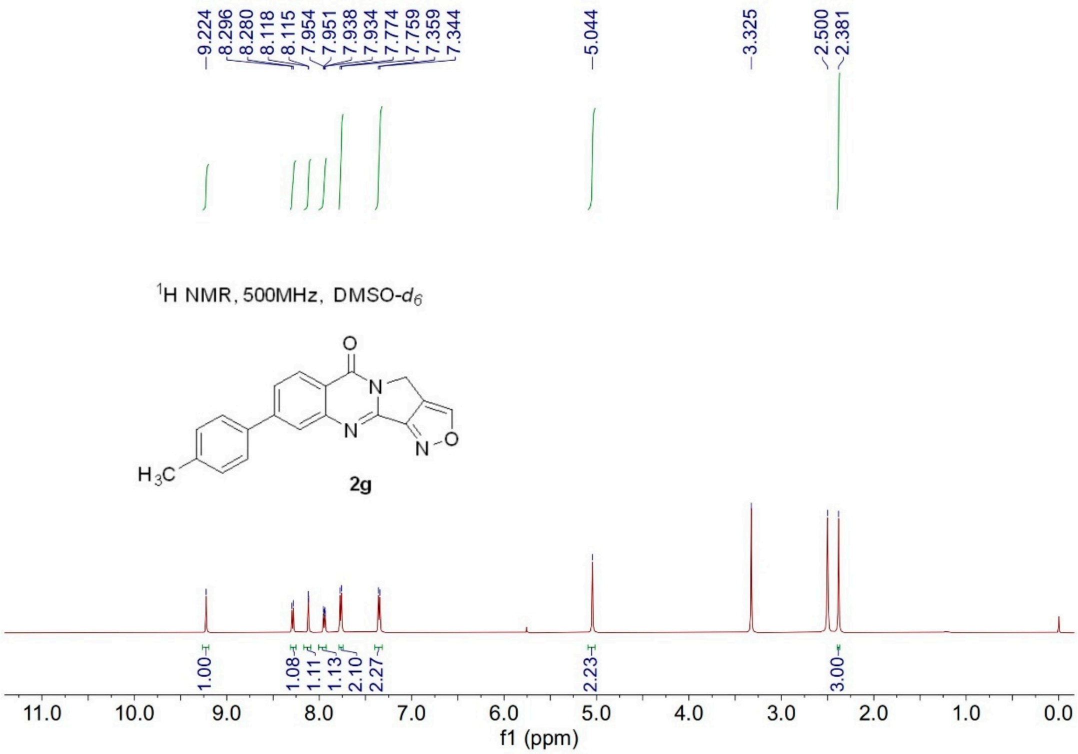
9.224
8.296
8.280
8.118
8.115
7.954
7.951
7.938
7.934
7.774
7.759
7.359
7.344
5.044
3.325
2.500
2.381
1H NMR, 500MHz, DMSO-d6
H3C
N
O
N
O
N
2g
1.00
1.08
1.11
1.13
2.10
2.27
2.23
3.00
f1 (ppm)

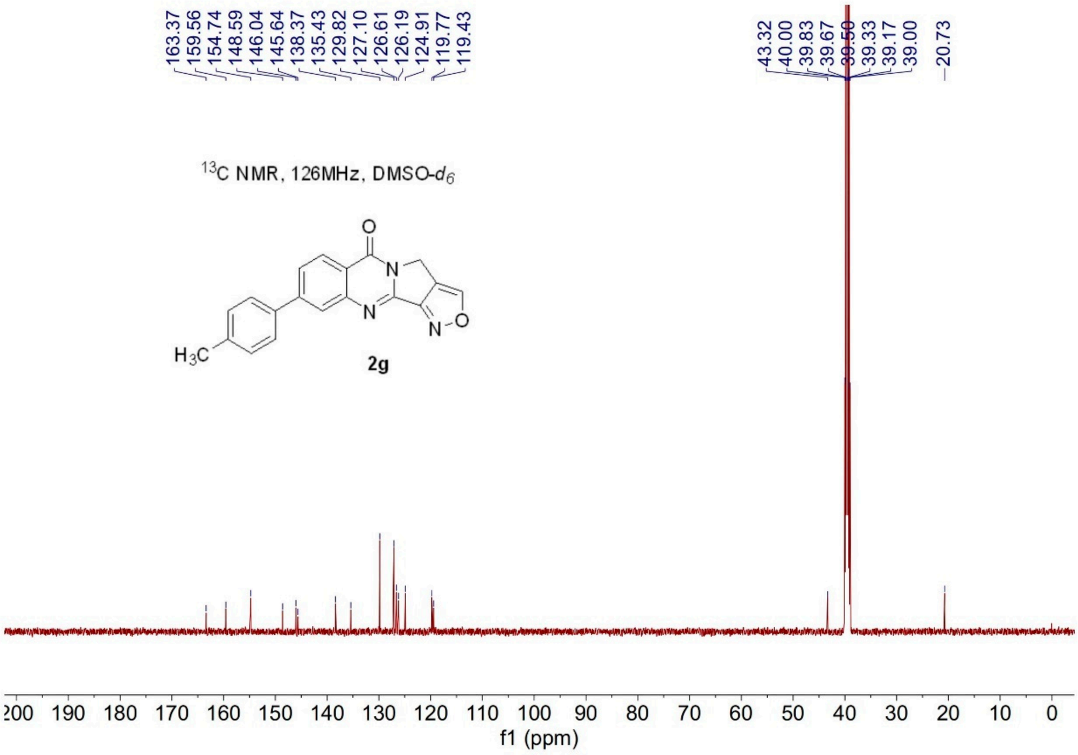
163.37
159.56
154.74
148.59
146.04
145.64
138.37
135.43
129.82
127.10
126.61
126.19
124.91
119.77
119.43
43.32
40.00
39.83
39.67
39.50
39.33
39.17
39.00
20.73
^{13}C NMR, 126MHz, DMSO-d_6
H$_3$C
2g
f1 (ppm)

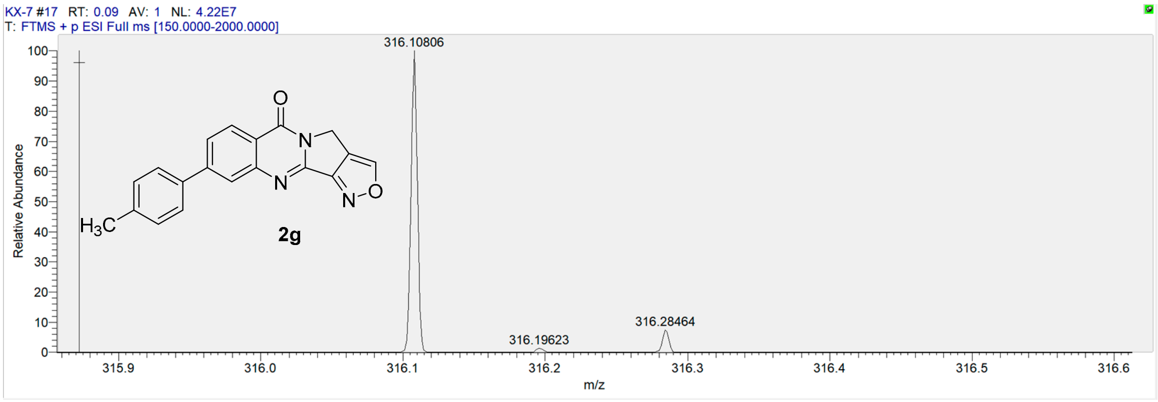
KX-7 #17 RT: 0.09 AV: 1 NL: 4.22E7
T: FTMS + p ESI Full ms [150.0000-2000.0000]
316.10806
316.19623
316.28464
Relative Abundance
m/z
H$_3$C
2g

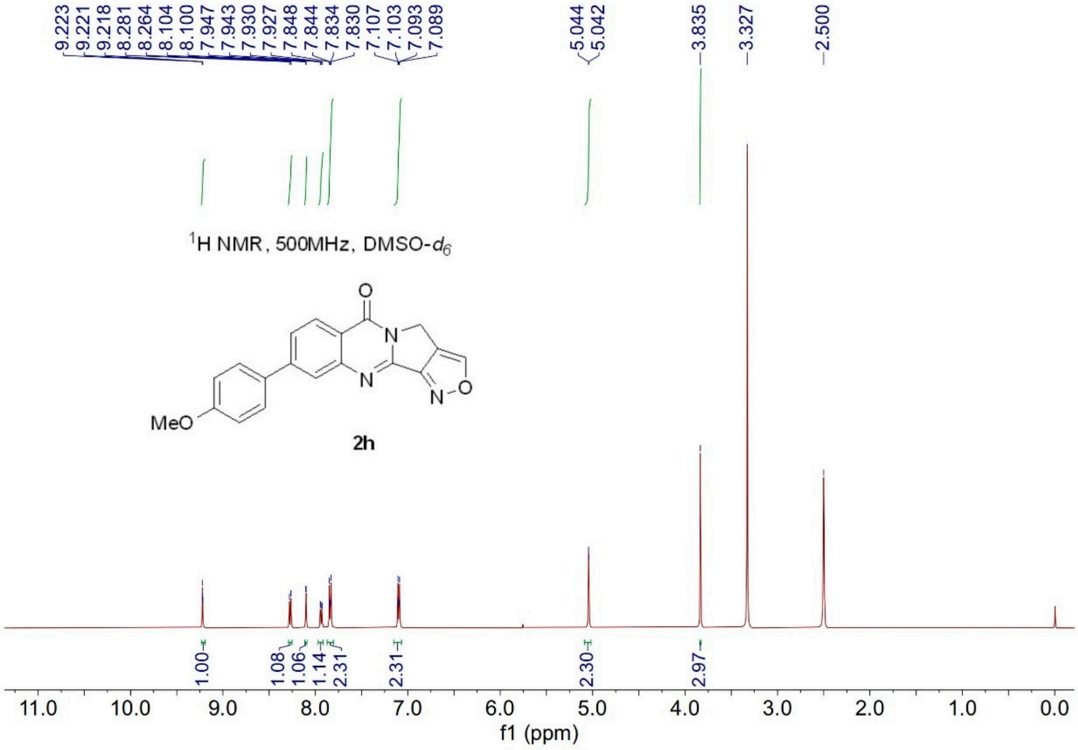
9.223
9.221
9.218
8.281
8.264
8.104
8.100
7.947
7.943
7.930
7.927
7.848
7.844
7.834
7.830
7.103
7.107
7.093
7.089
5.044
5.042
3.835
3.327
2.500
1H NMR, 500MHz, DMSO-d6
O
N
N
N-O
MeO
2h
1.00
1.08
1.06
1.14
2.31
2.31
2.30
2.97
11.0 10.0 9.0 8.0 7.0 6.0 5.0 4.0 3.0 2.0 1.0 0.0
f1 (ppm)

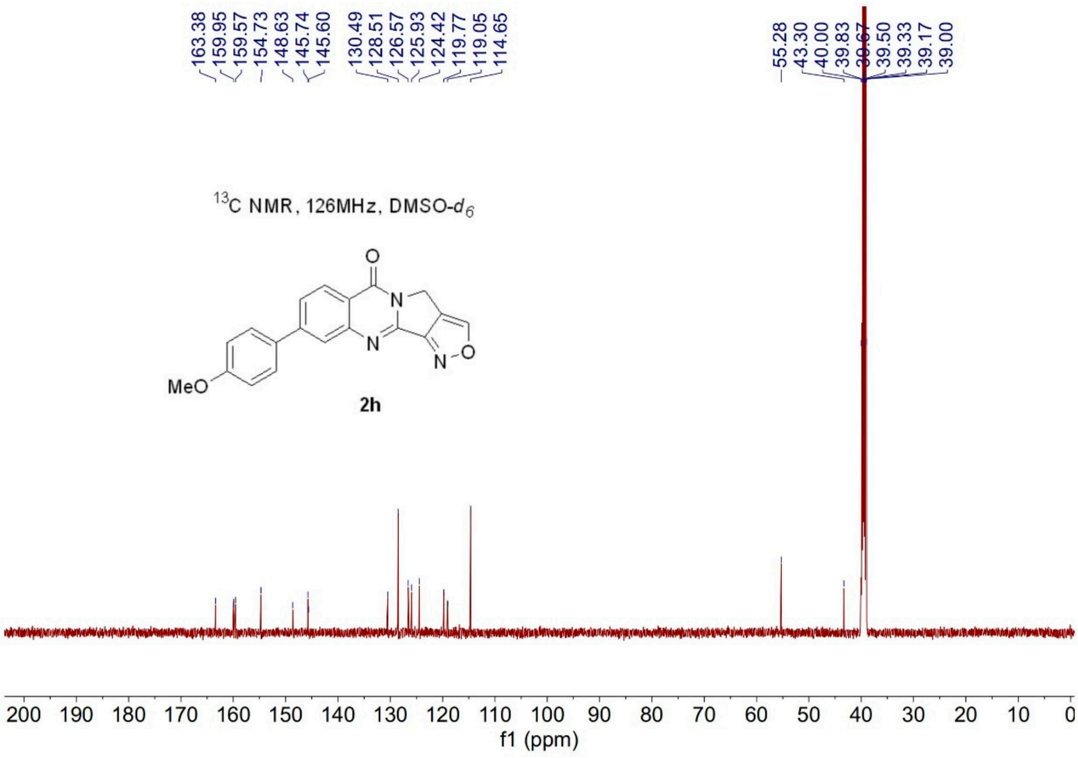
163.38
159.95
159.57
154.73
148.63
145.74
145.60
130.49
128.51
126.57
125.93
124.42
119.77
119.05
114.65
55.28
43.30
40.00
39.83
39.67
39.50
39.33
39.17
39.00
13C NMR, 126MHz, DMSO-d6
O
N
N
N-O
MeO
2h
200 190 180 170 160 150 140 130 120 110 100 90 80 70 60 50 40 30 20 10 0
f1 (ppm)

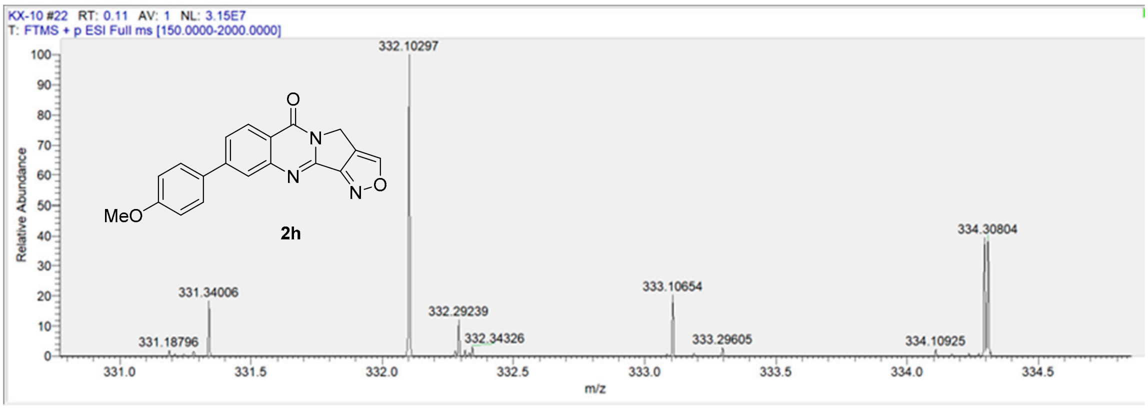
KX-10 #22 RT: 0.11 AV: 1 NL: 3.15E7
T: FTMS + p ESI Full ms [150.0000-2000.0000]
332.10297
331.34006
331.18796
332.29239
332.34326
333.10654
333.29605
334.30804
334.10925
Relative Abundance
m/z
MeO
2h

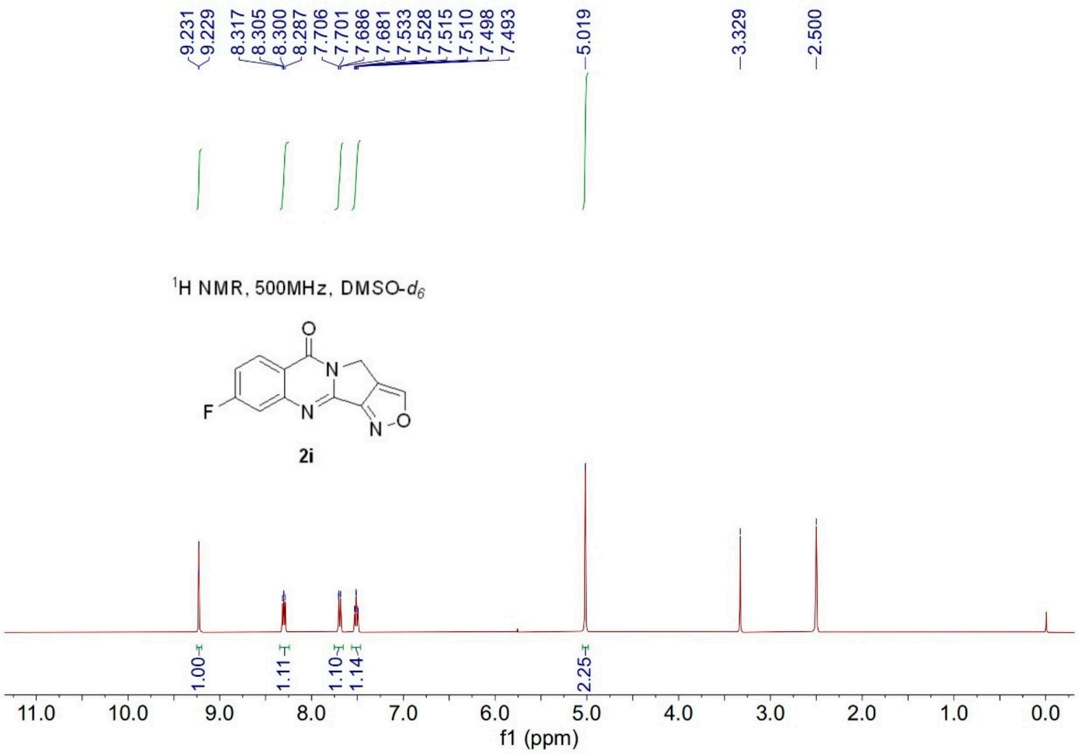
9.231
9.229
8.317
8.305
8.300
8.287
7.706
7.701
7.686
7.681
7.533
7.528
7.515
7.510
7.498
7.493
5.019
3.329
2.500
^{1}H NMR, 500MHz, DMSO-d_6
F
2i
1.00
1.11
1.10
1.14
2.25
f1 (ppm)

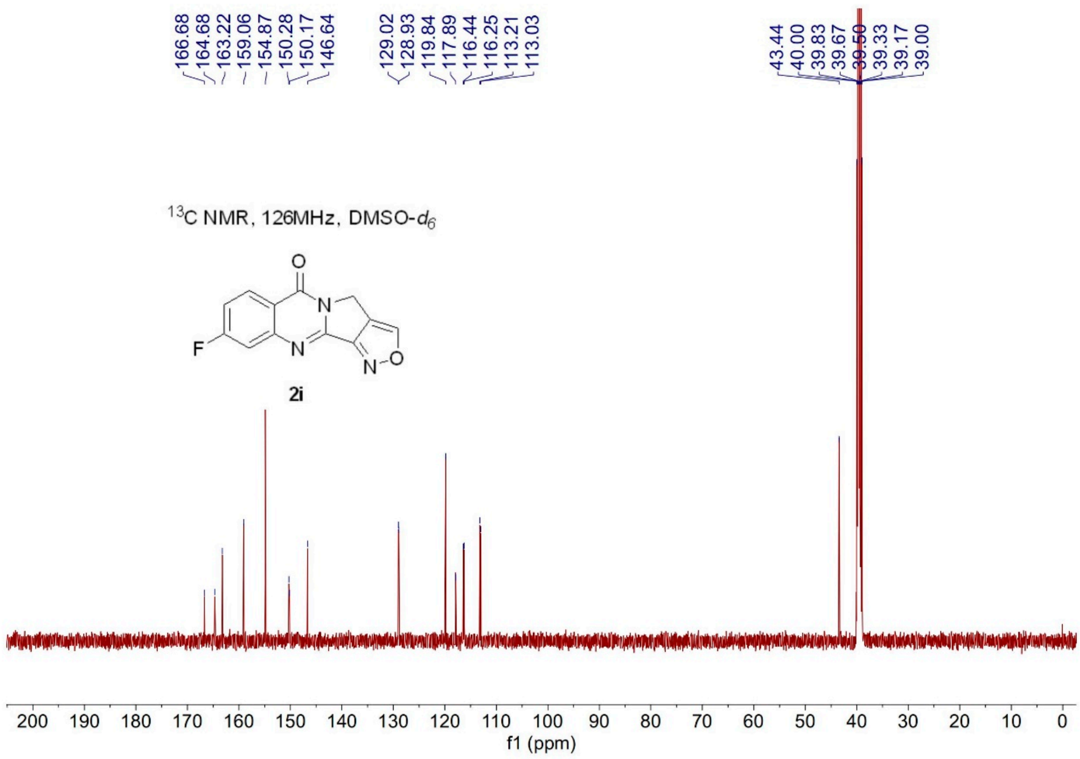
166.68
164.68
163.22
159.06
154.87
150.28
150.17
146.64
129.02
128.93
119.84
117.89
116.44
116.25
113.21
113.03
43.44
40.00
39.83
39.67
39.50
39.33
39.17
39.00
13C NMR, 126MHz, DMSO-d6
O
N
F
N
N
O
2i
200 190 180 170 160 150 140 130 120 110 100 90 80 70 60 50 40 30 20 10 0
f1 (ppm)

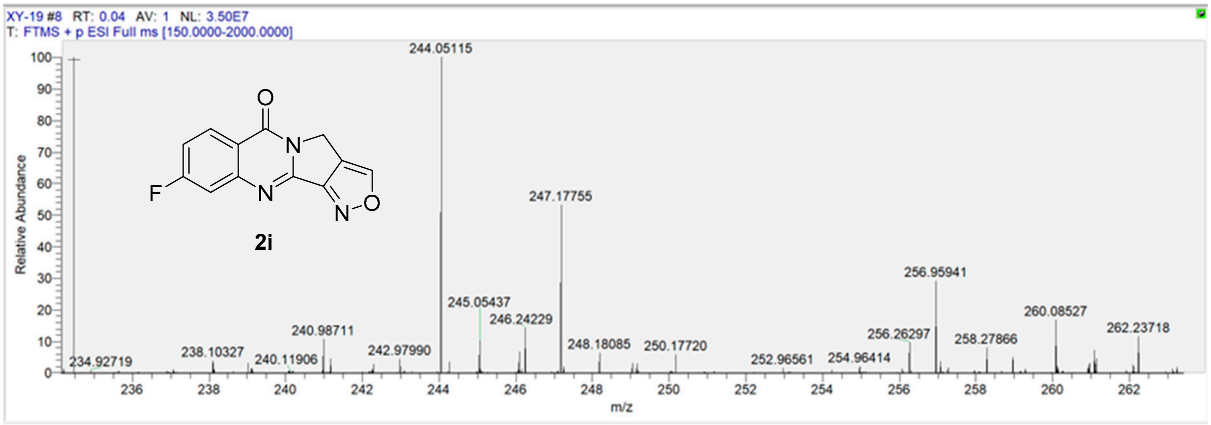
XY-19 #8 RT: 0.04 AV: 1 NL: 3.50E7
T: FTMS + p ESI Full ms [150.0000-2000.0000]
244.05115
247.17755
256.95941
245.05437
246.24229
260.08527
262.23718
240.98711
256.26297
258.27866
248.18085
250.17720
238.10327
242.97990
234.92719
240.11906
252.96561
254.96414
Relative Abundance
m/z
2i

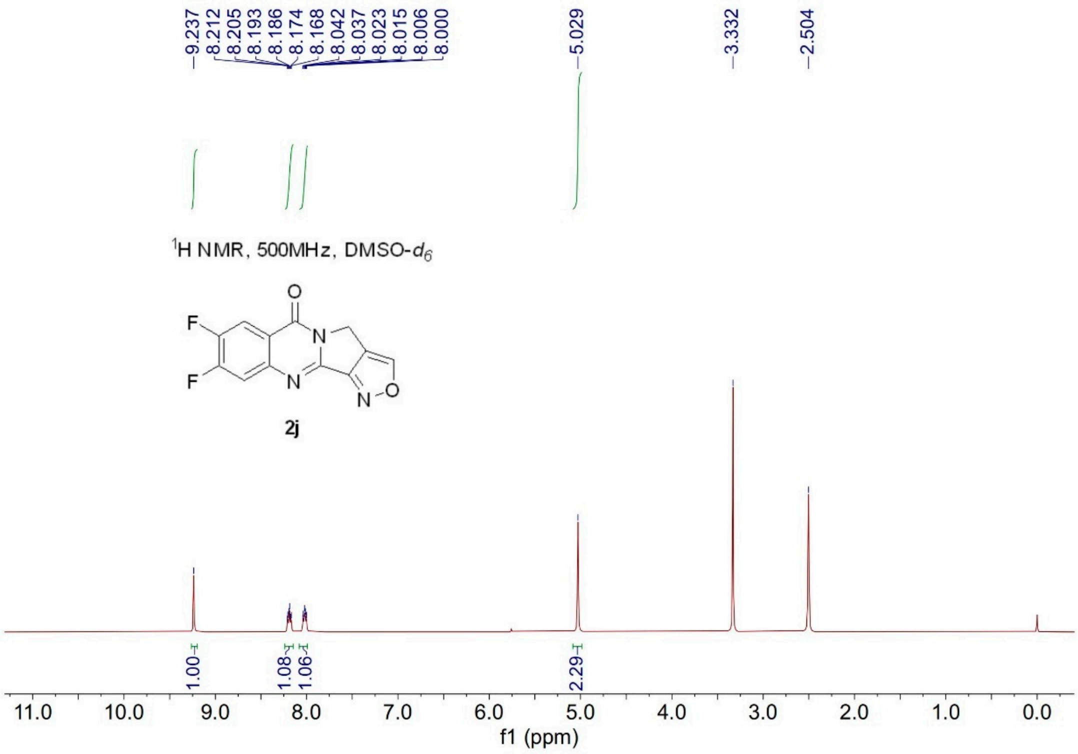
9.237
8.212
8.205
8.193
8.186
8.174
8.168
8.042
8.037
8.023
8.015
8.006
8.000
5.029
3.332
2.504
1H NMR, 500MHz, DMSO-d6
2j
1.00
1.08
1.06
2.29
11.0 10.0 9.0 8.0 7.0 6.0 5.0 4.0 3.0 2.0 1.0 0.0
f1 (ppm)

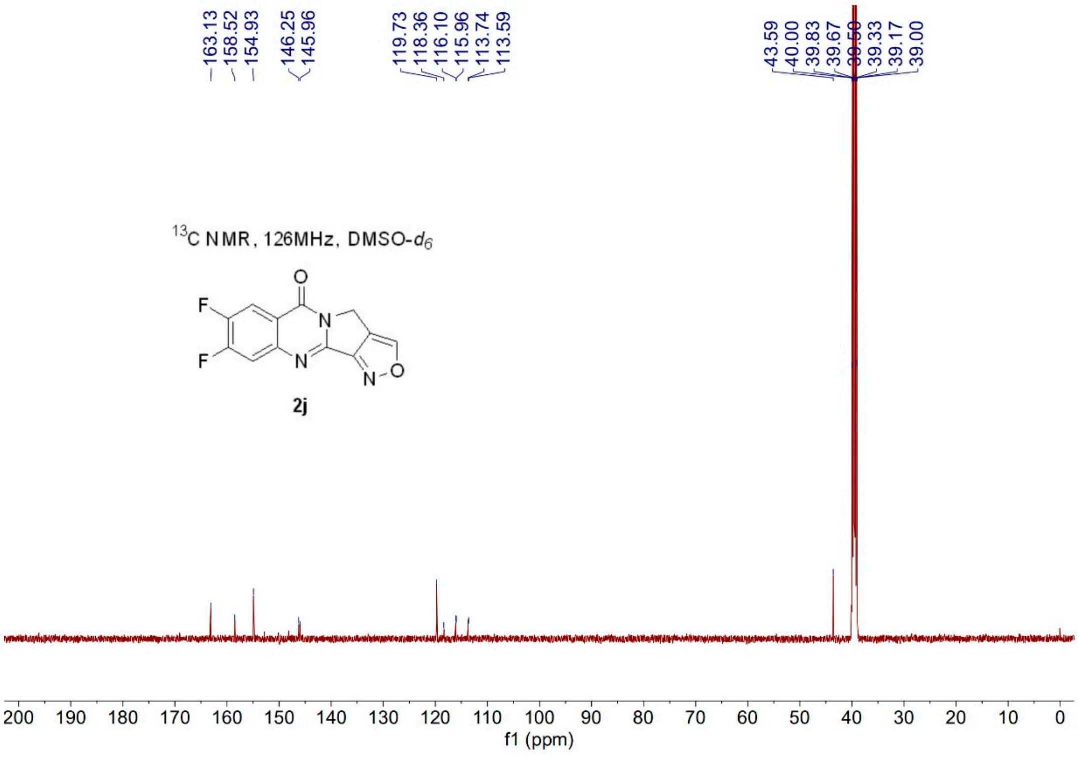
163.13
158.52
154.93
146.25
145.96
119.73
118.36
116.10
115.96
113.74
113.59
43.59
40.00
39.83
39.67
39.50
39.33
39.17
39.00
13C NMR, 126MHz, DMSO-d6
2j
200 190 180 170 160 150 140 130 120 110 100 90 80 70 60 50 40 30 20 10 0
f1 (ppm)

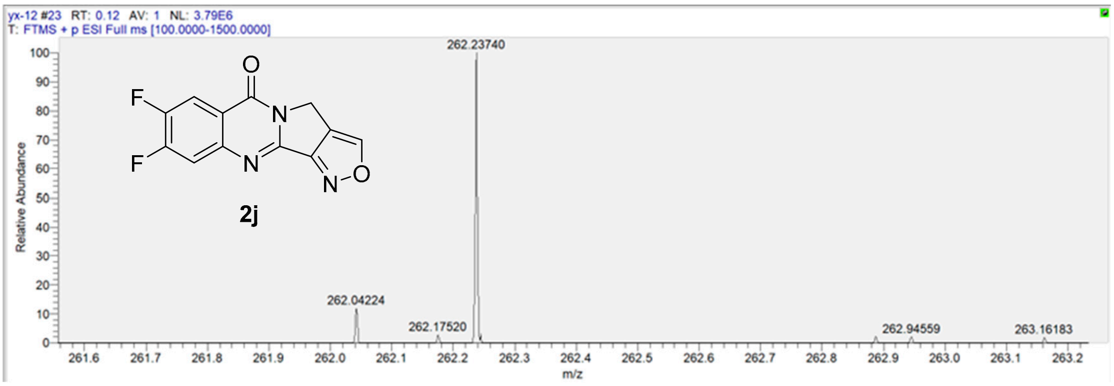

yx-12 #23 RT: 0.12 AV: 1 NL: 3.79E6
T: FTMS + p ESI Full ms [100.0000-1500.0000]
262.23740
262.04224
262.17520
262.94559
263.16183
Relative Abundance
m/z
2j

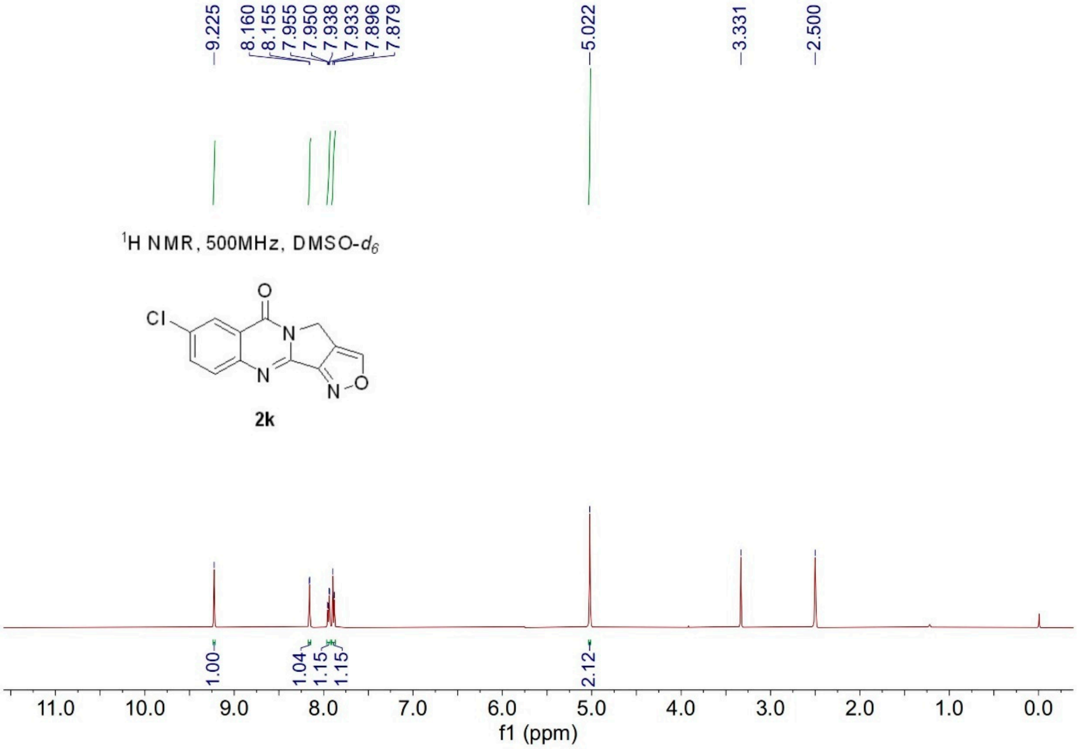

9.225
8.160
8.155
7.955
7.950
7.938
7.933
7.896
7.879
5.022
3.331
2.500
1H NMR, 500MHz, DMSO-d6
2k
1.00
1.04
1.15
1.15
2.12
f1 (ppm)

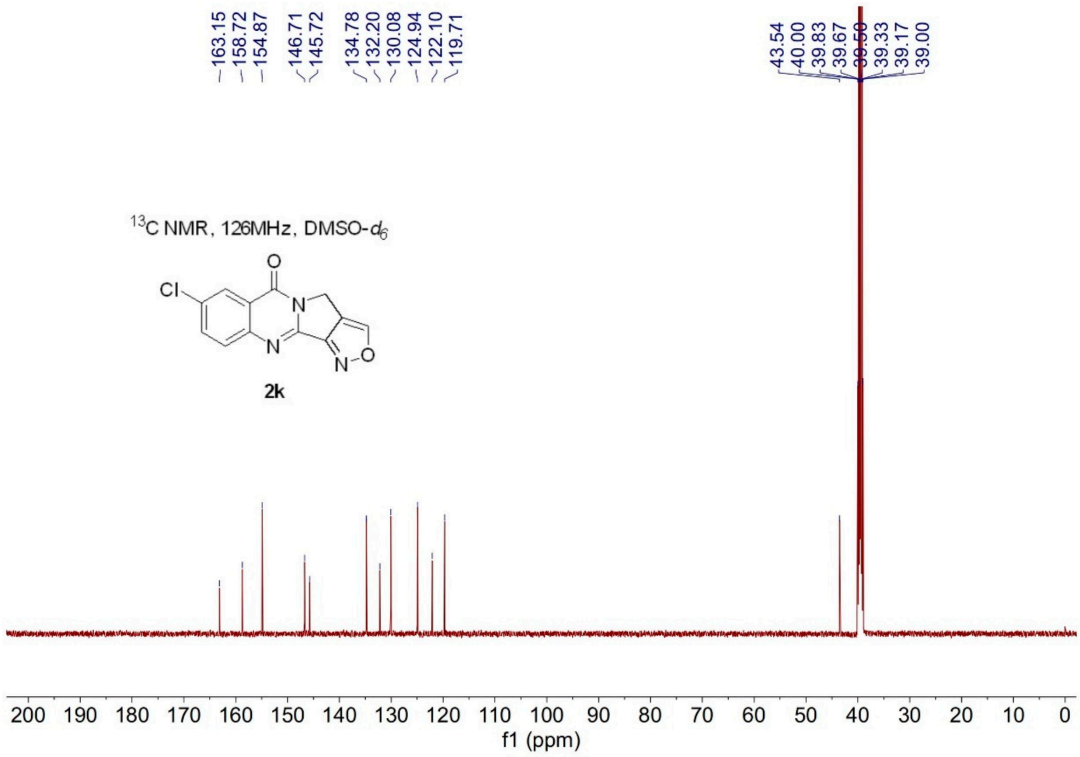
163.15
158.72
154.87
146.71
145.72
134.78
132.20
130.08
124.94
122.10
119.71
43.54
40.00
39.83
39.67
39.50
39.33
39.17
39.00
13C NMR, 126MHz, DMSO-d6
2k
200 190 180 170 160 150 140 130 120 110 100 90 80 70 60 50 40 30 20 10 0
f1 (ppm)

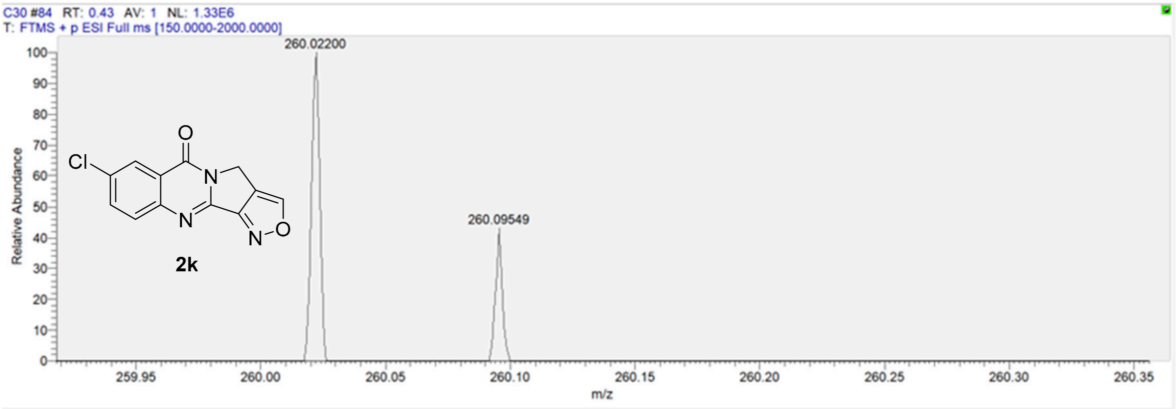
C30 #84 RT: 0.43 AV: 1 NL: 1.33E6
T: FTMS + p ESI Full ms [150.0000-2000.0000]
260.02200
260.09549
Relative Abundance
2k
m/z

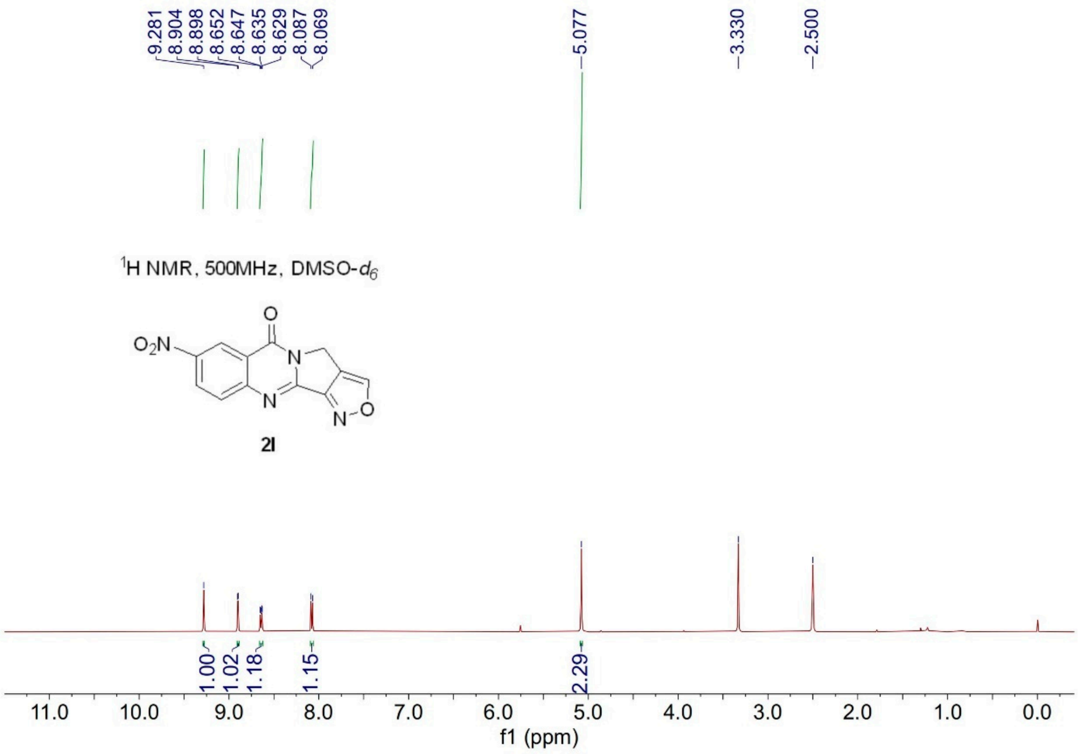
9.281
8.904
8.898
8.652
8.647
8.635
8.629
8.087
8.069
5.077
3.330
2.500
^{1}H NMR, 500MHz, DMSO-d_6
O_2N
2l
1.00
1.02
1.18
1.15
2.29
11.0 10.0 9.0 8.0 7.0 6.0 5.0 4.0 3.0 2.0 1.0 0.0
f1 (ppm)

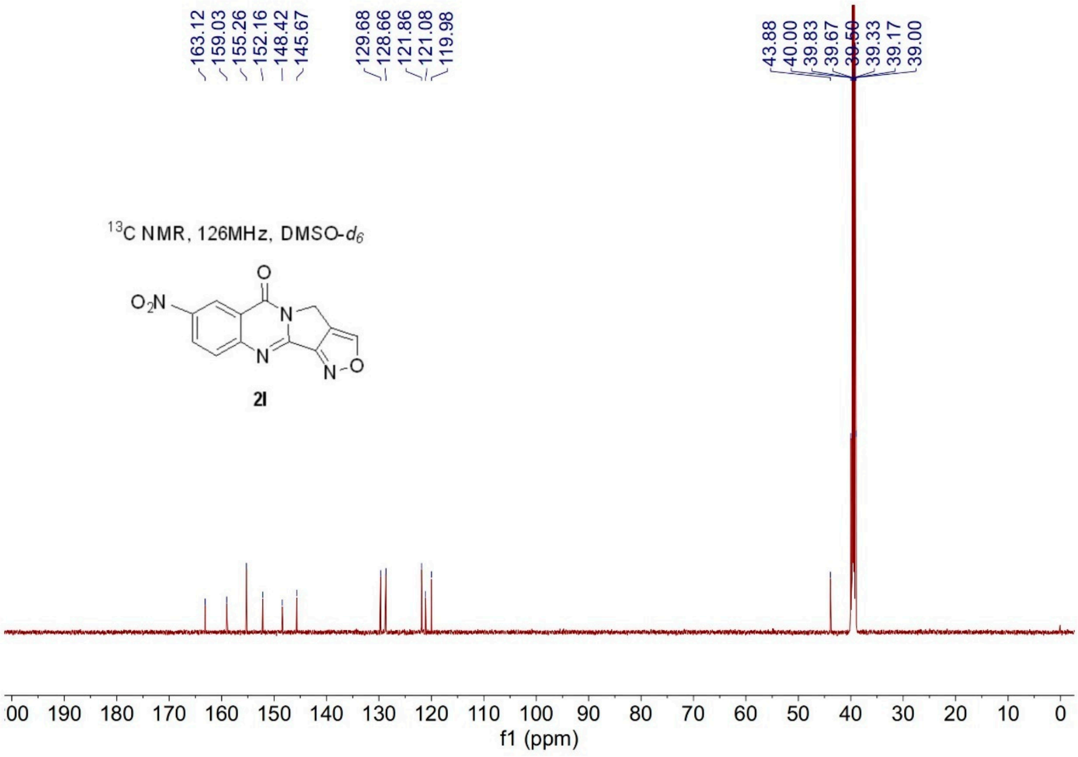
163.12
159.03
155.26
152.16
148.42
145.67
129.68
128.66
121.86
121.08
119.98
43.88
40.00
39.83
39.67
39.50
39.33
39.17
39.00
^{13}C NMR, 126MHz, DMSO-d_6
O_2N
2l
00 190 180 170 160 150 140 130 120 110 100 90 80 70 60 50 40 30 20 10 0
f1 (ppm)

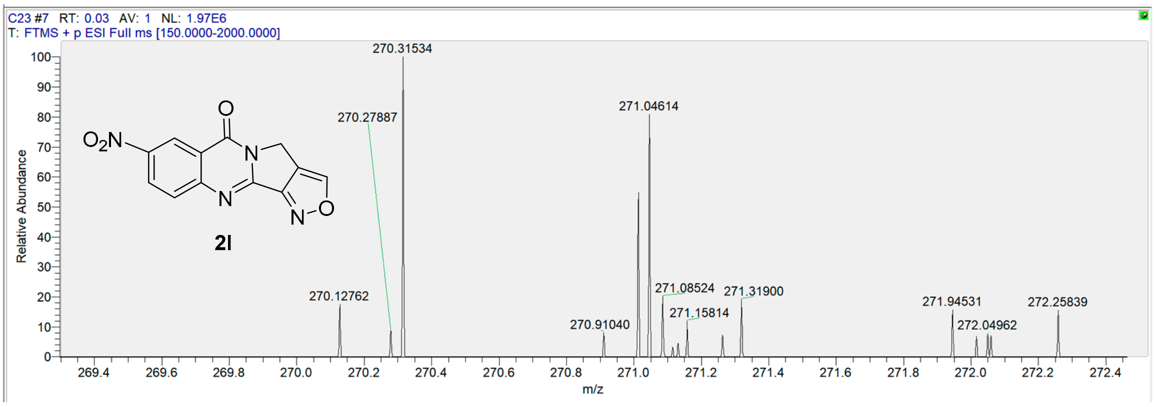

C23 #7 RT: 0.03 AV: 1 NL: 1.97E6
T: FTMS + p ESI Full ms [150.0000-2000.0000]
270.31534
270.27887
271.04614
270.12762
270.91040
271.08524
271.15814
271.31900
271.94531
272.04962
272.25839
Relative Abundance
m/z
O
O_2N
N
N
N-O
2l

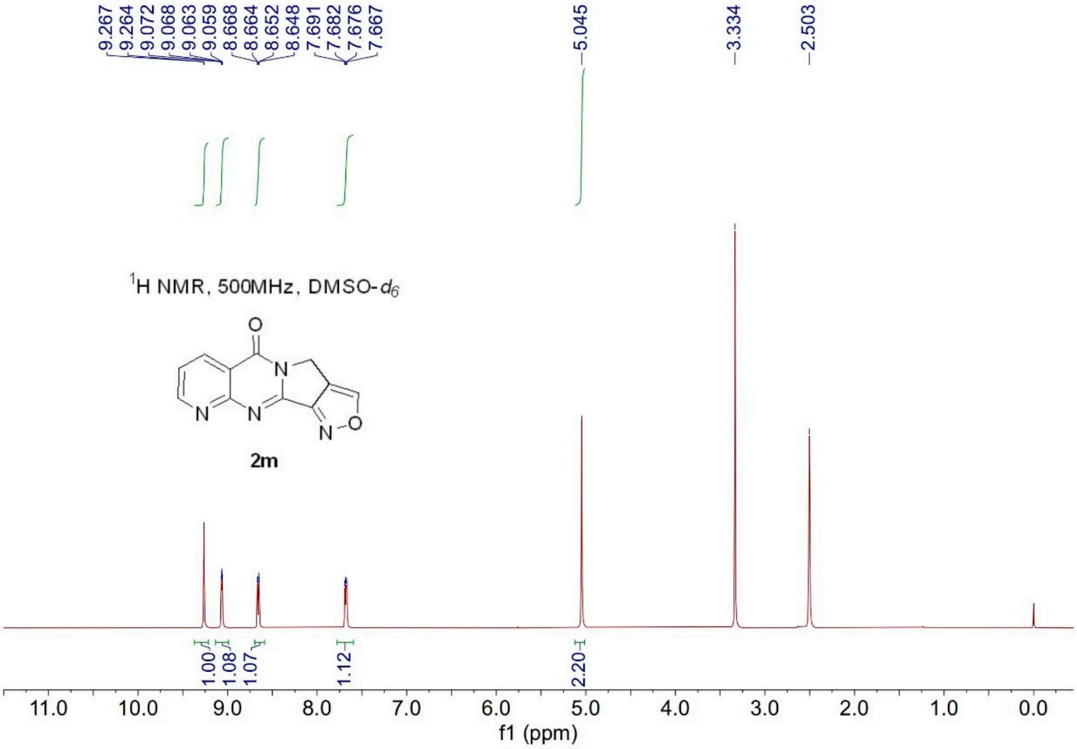

9.267
9.264
9.072
9.068
9.063
9.059
8.668
8.664
8.652
8.648
7.691
7.682
7.676
7.667
5.045
3.334
2.503
^{1}H NMR, 500MHz, DMSO-d_6
O
N
N
N
N-O
2m
1.00
1.08
1.07
1.12
2.20
f1 (ppm)

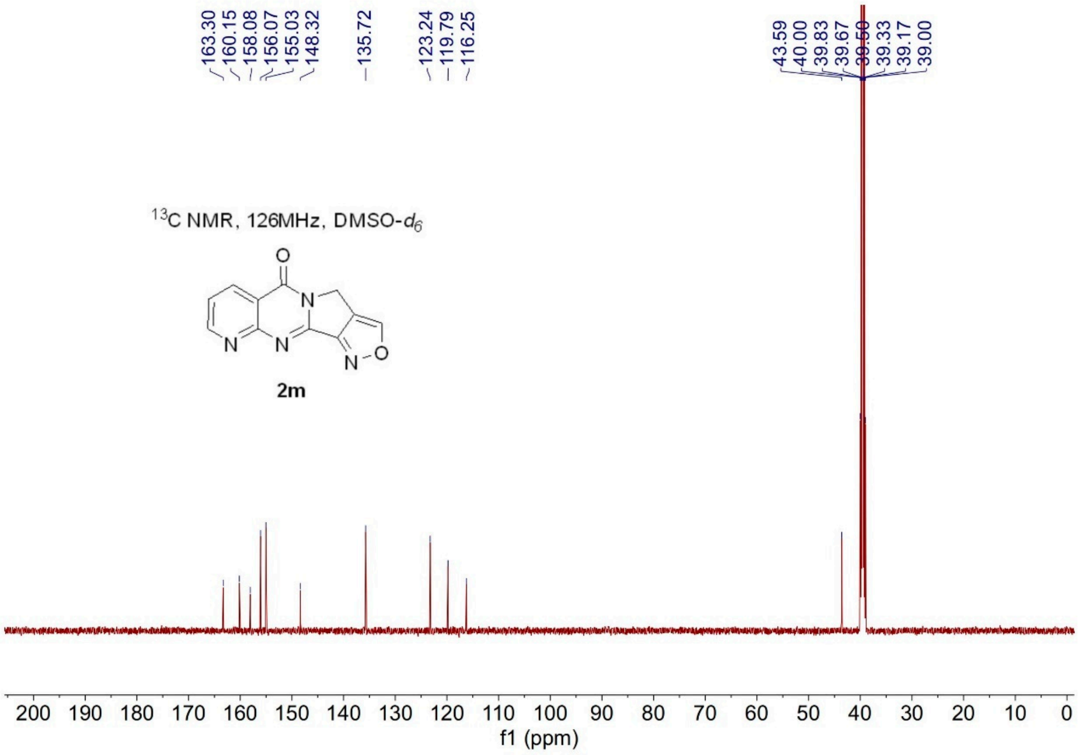

163.30
160.15
158.08
156.07
155.03
148.32
135.72
123.24
119.79
116.25
43.59
40.00
39.83
39.67
39.50
39.33
39.17
39.00
^{13}C NMR, 126MHz, DMSO-d_6
2m
200 190 180 170 160 150 140 130 120 110 100 90 80 70 60 50 40 30 20 10 0
f1 (ppm)

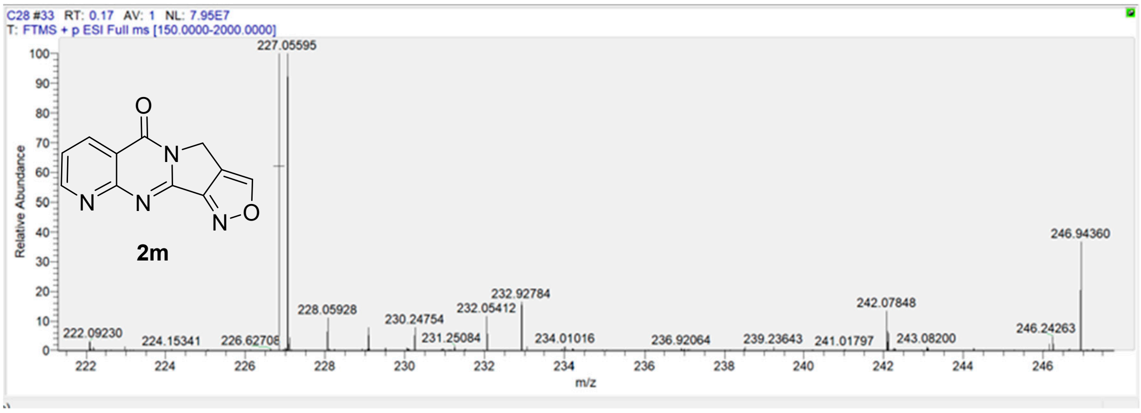

C28 #33 RT: 0.17 AV: 1 NL: 7.95E7
T: FTMS + p ESI Full ms [150.0000-2000.0000]
227.05595
Relative Abundance
2m
222.09230
224.15341
226.62708
228.05928
230.24754
231.25084
232.05412
232.92784
234.01016
236.92064
239.23643
241.01797
242.07848
243.08200
246.24263
246.94360
m/z

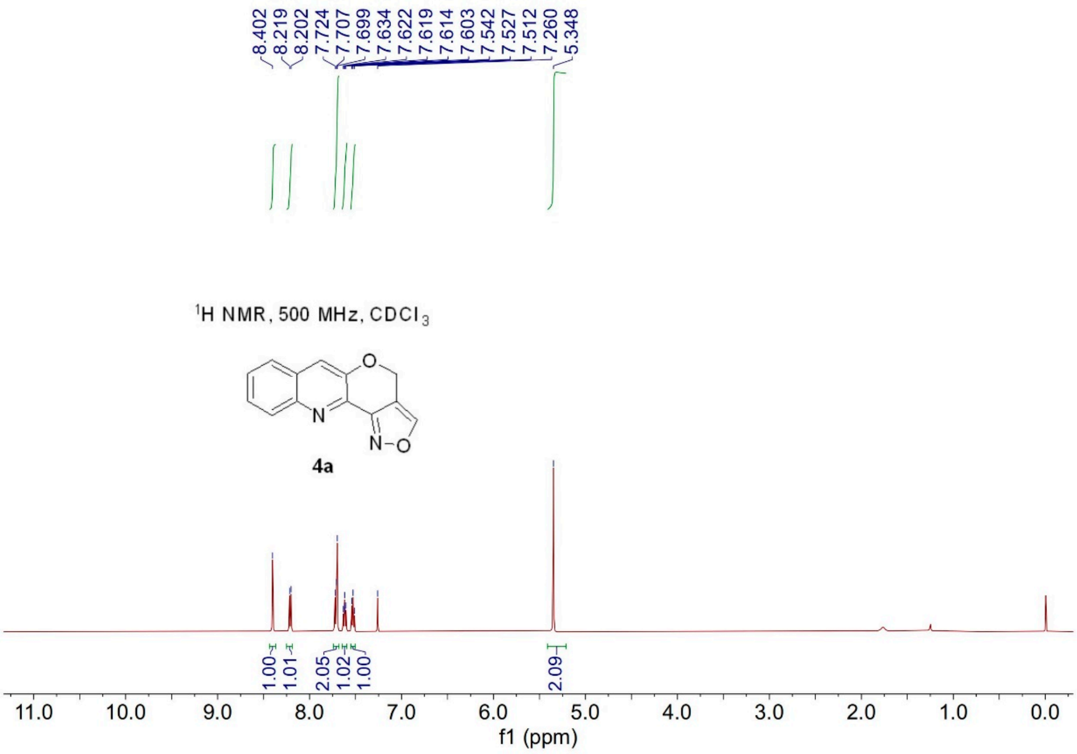
8.402
8.219
8.202
7.724
7.707
7.699
7.634
7.622
7.619
7.614
7.603
7.542
7.527
7.512
7.260
5.348
1H NMR, 500 MHz, CDCl3
N
O
N-O
4a
1.00
1.01
2.05
1.02
1.00
2.09
11.0 10.0 9.0 8.0 7.0 6.0 5.0 4.0 3.0 2.0 1.0 0.0
f1 (ppm)

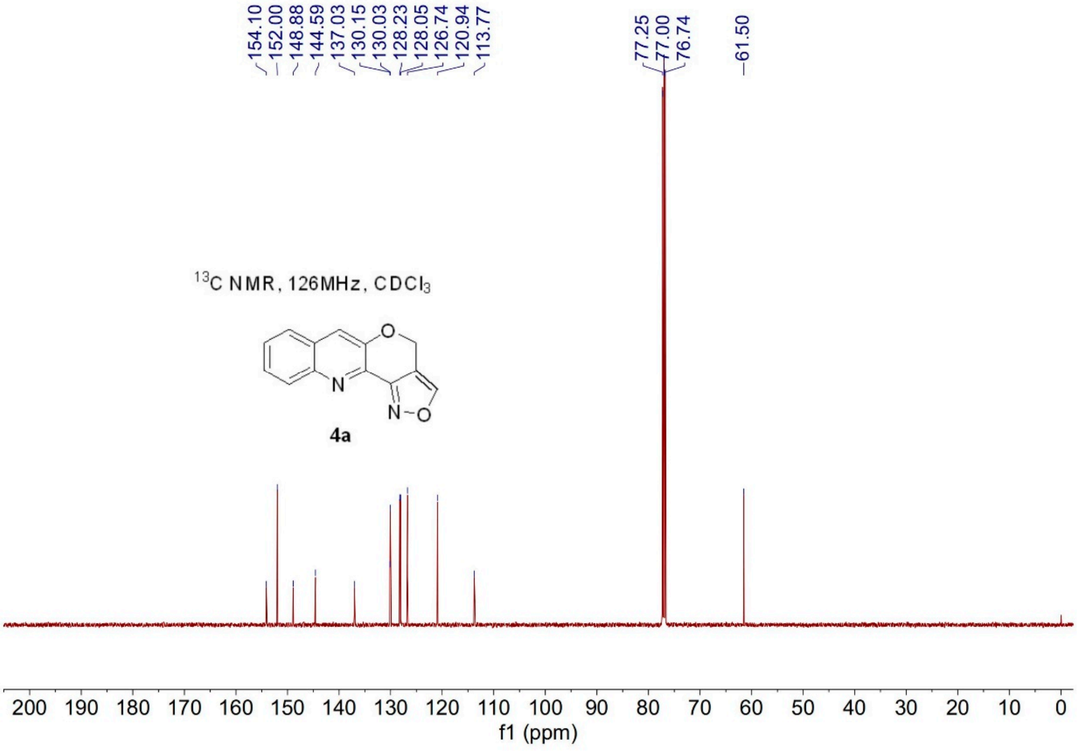
154.10
152.00
148.88
144.59
137.03
130.15
130.03
128.23
128.05
126.74
120.94
113.77
77.25
77.00
76.74
61.50
13C NMR, 126MHz, CDCl3
N
O
N-O
4a
200 190 180 170 160 150 140 130 120 110 100 90 80 70 60 50 40 30 20 10 0
f1 (ppm)

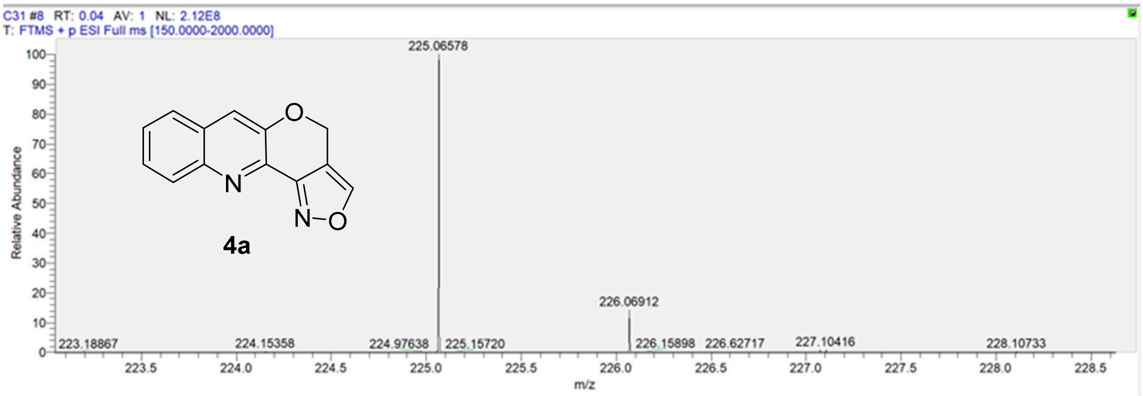
C31 #8 RT: 0.04 AV: 1 NL: 2.12E8
T: FTMS + p ESI Full ms [150.0000-2000.0000]
225.06578
226.06912
223.18867
224.15358
224.97638
225.15720
226.15898
226.62717
227.10416
228.10733
Relative Abundance
m/z
4a

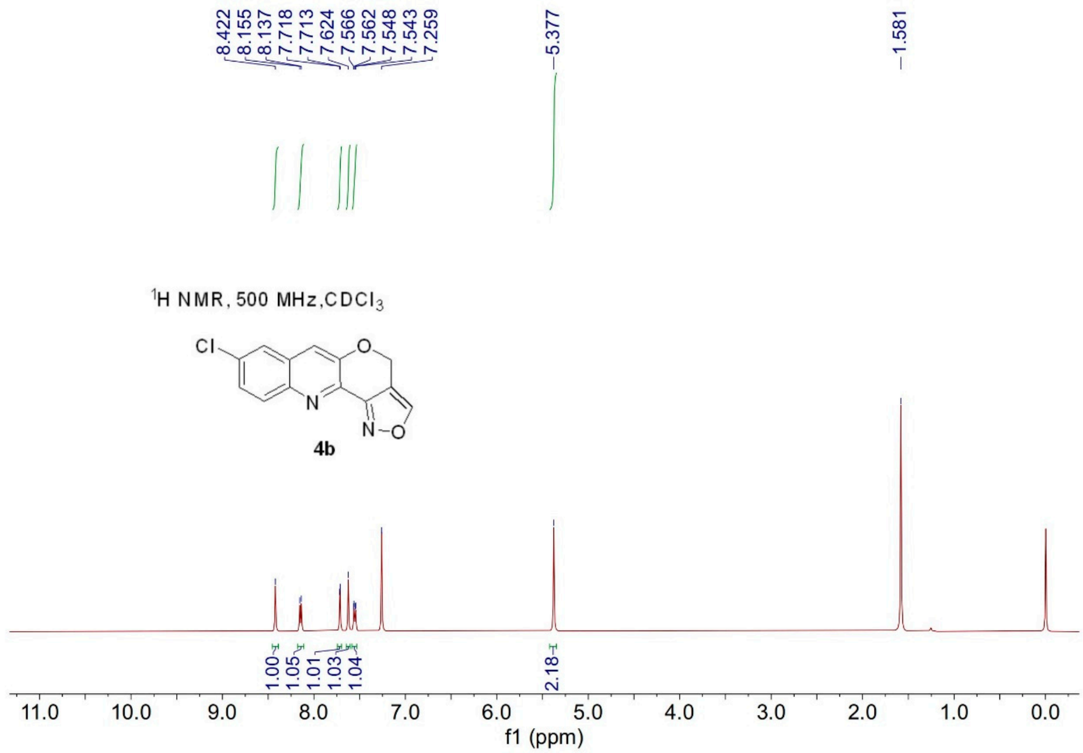
8.422
8.155
8.137
7.718
7.713
7.624
7.566
7.562
7.548
7.543
7.259
5.377
1.581
^{1}H NMR, 500 MHz, $CDCl_3$
Cl
4b
1.00
1.05
1.01
1.03
1.04
2.18
f1 (ppm)

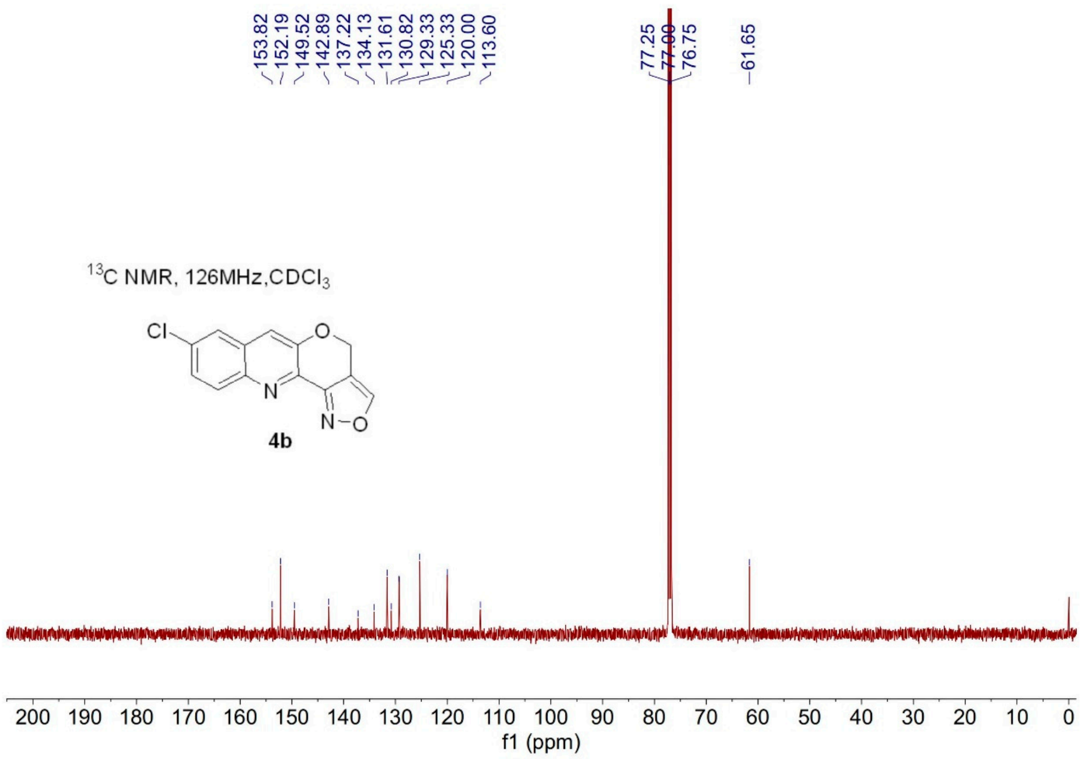
153.82
152.19
149.52
142.89
137.22
134.13
131.61
130.82
129.33
125.33
120.00
113.60
77.25
77.00
76.75
61.65
^{13}C NMR, 126MHz,CDCl$_3$
Cl
O
N
N-O
4b
200 190 180 170 160 150 140 130 120 110 100 90 80 70 60 50 40 30 20 10 0
f1 (ppm)

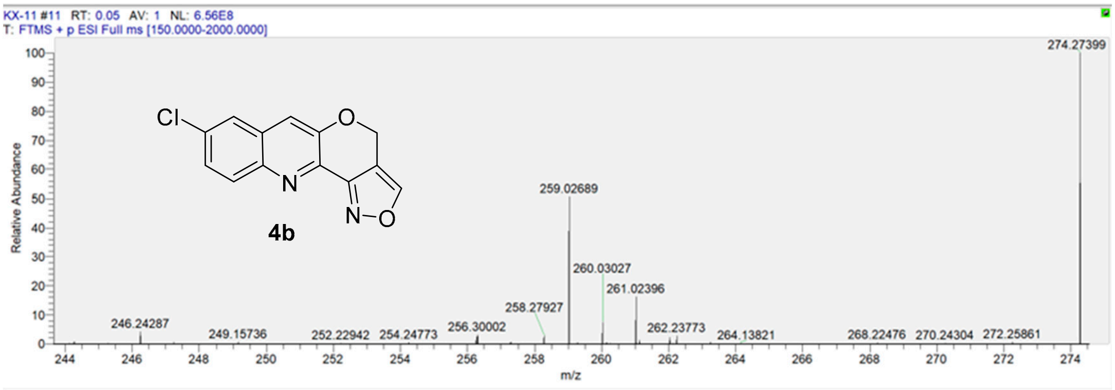
KX-11 #11 RT: 0.05 AV: 1 NL: 6.56E8
T: FTMS + p ESI Full ms [150.0000-2000.0000]
Relative Abundance
Cl
O
N
N-O
4b
274.27399
259.02689
260.03027
261.02396
258.27927
262.23773
246.24287
249.15736
252.22942
254.24773
256.30002
264.13821
268.22476
270.24304
272.25861
m/z

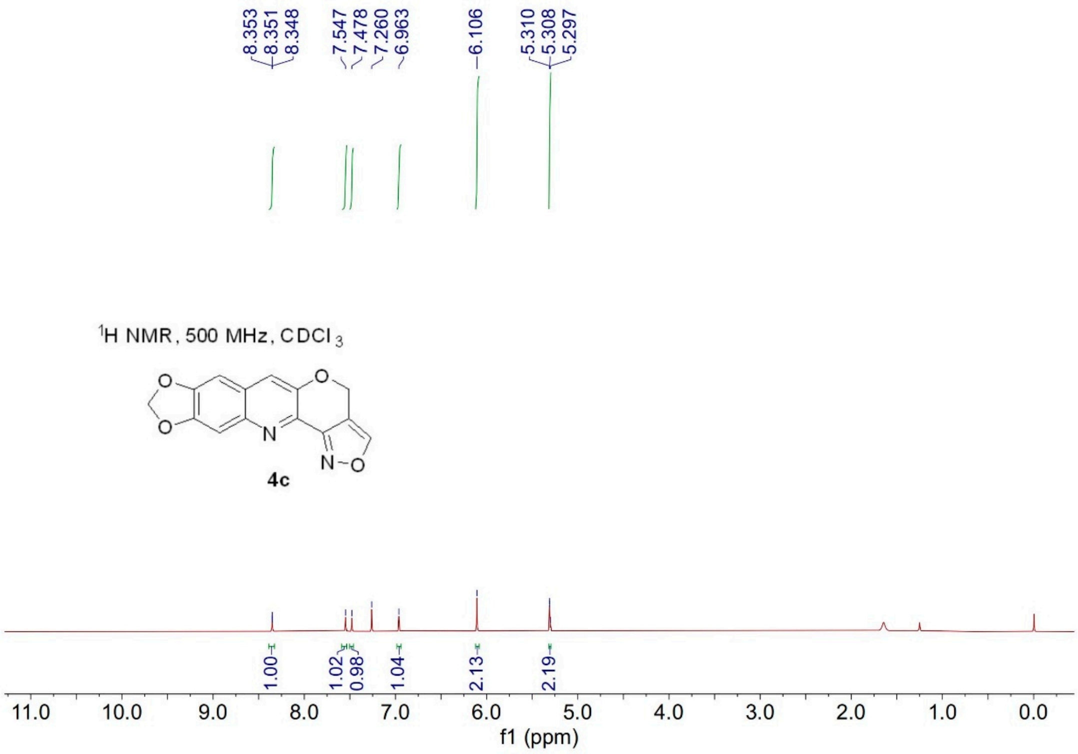
8.353
8.351
8.348
7.547
7.478
7.260
6.963
6.106
5.310
5.308
5.297
1H NMR, 500 MHz, CDCl3
N
O
O
O
N-O
4c
1.00
1.02
0.98
1.04
2.13
2.19
11.0 10.0 9.0 8.0 7.0 6.0 5.0 4.0 3.0 2.0 1.0 0.0
f1 (ppm)

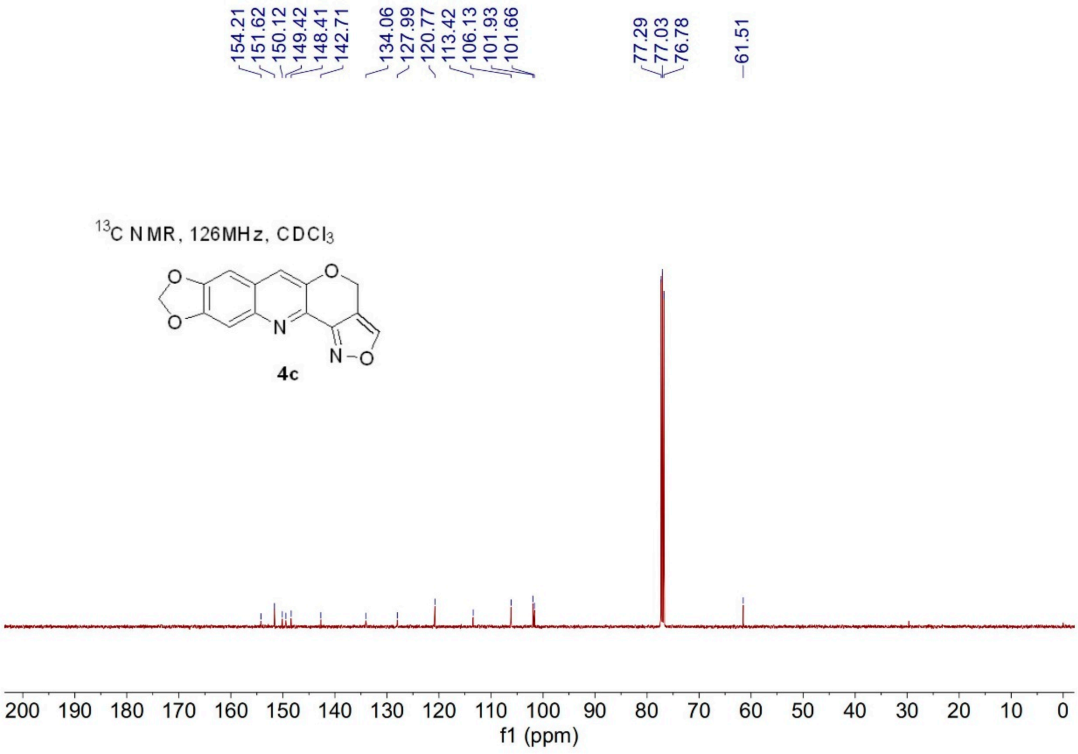
154.21
151.62
150.12
149.42
148.41
142.71
134.06
127.99
120.77
113.42
106.13
101.93
101.66
77.29
77.03
76.78
61.51
13C NMR, 126MHz, CDCl3
4c
200 190 180 170 160 150 140 130 120 110 100 90 80 70 60 50 40 30 20 10 0
f1 (ppm)

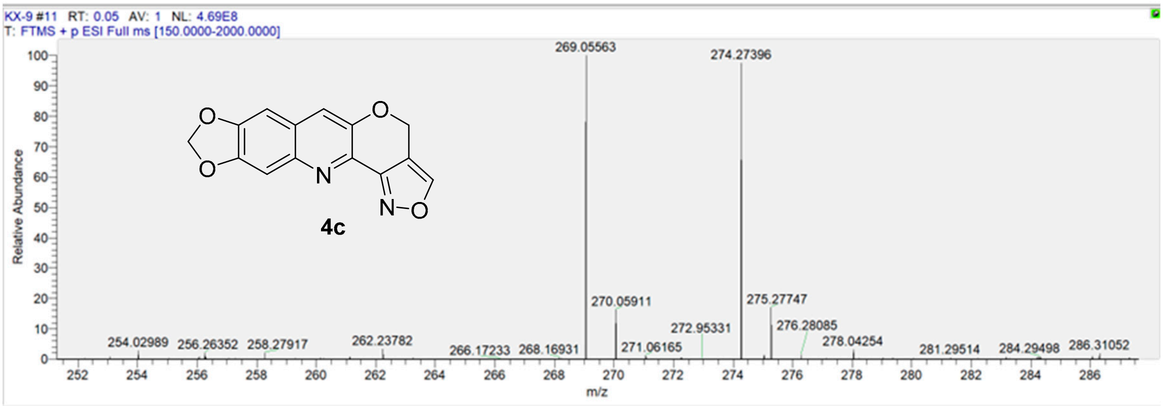

KX-9 #11 RT: 0.05 AV: 1 NL: 4.69E8
T: FTMS + p ESI Full ms [150.0000-2000.0000]
269.05563
274.27396
270.05911
275.27747
276.28085
272.95331
278.04254
271.06165
254.02989
256.26352
258.27917
262.23782
266.17233
268.16931
281.29514
284.29498
286.31052
Relative Abundance
m/z
4c

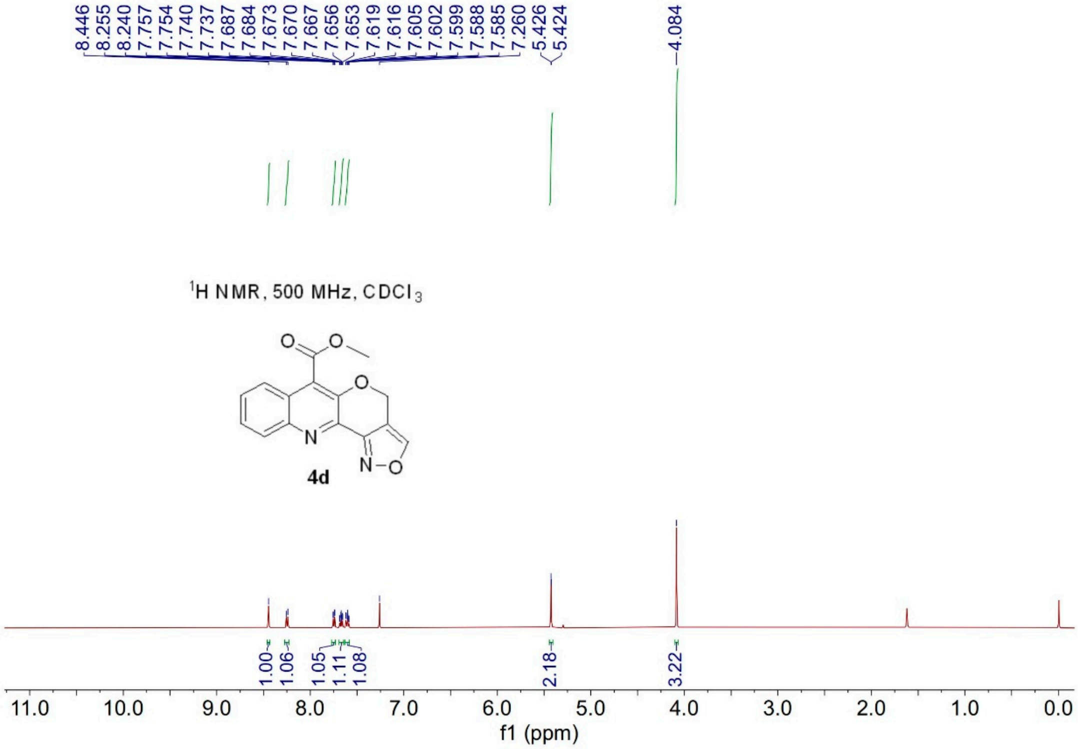

8.446
8.255
8.240
7.757
7.754
7.740
7.737
7.687
7.684
7.673
7.670
7.667
7.656
7.653
7.619
7.616
7.605
7.602
7.599
7.588
7.585
7.260
5.426
5.424
4.084
1H NMR, 500 MHz, CDCl3
4d
1.00
1.06
1.05
1.11
1.08
2.18
3.22
f1 (ppm)

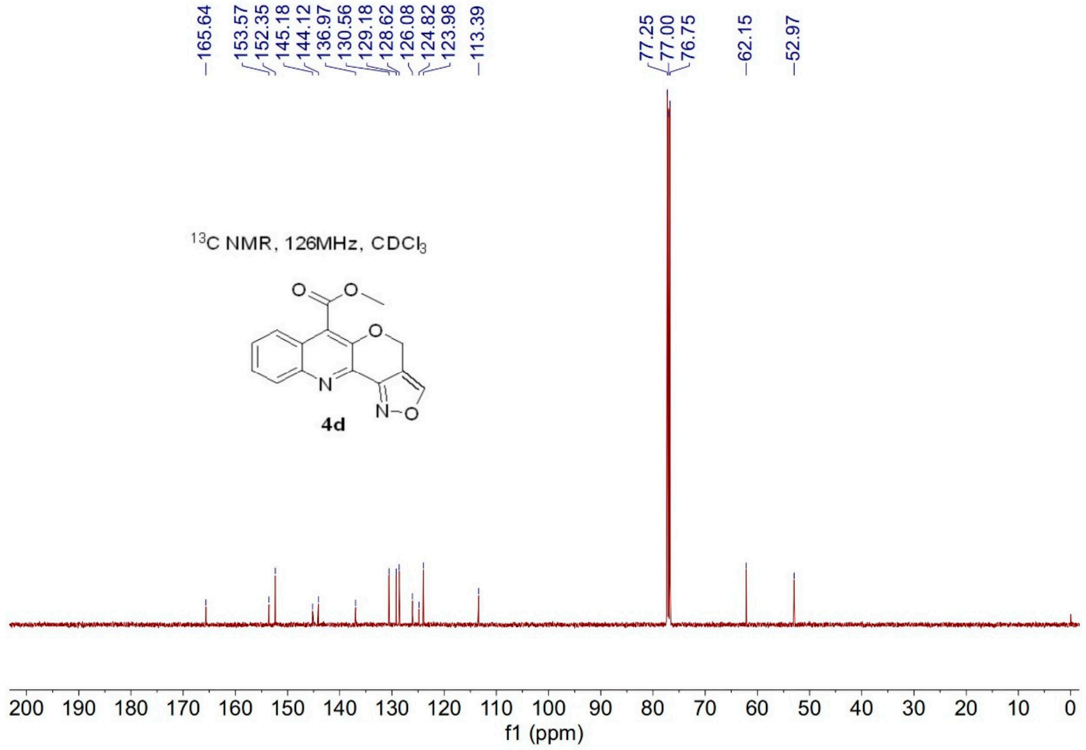

165.64
153.57
152.35
145.18
144.12
136.97
130.56
129.18
128.62
126.08
124.82
123.98
113.39
77.25
77.00
76.75
62.15
52.97
^{13}C NMR, 126MHz, CDCl$_3$
4d
f1 (ppm)

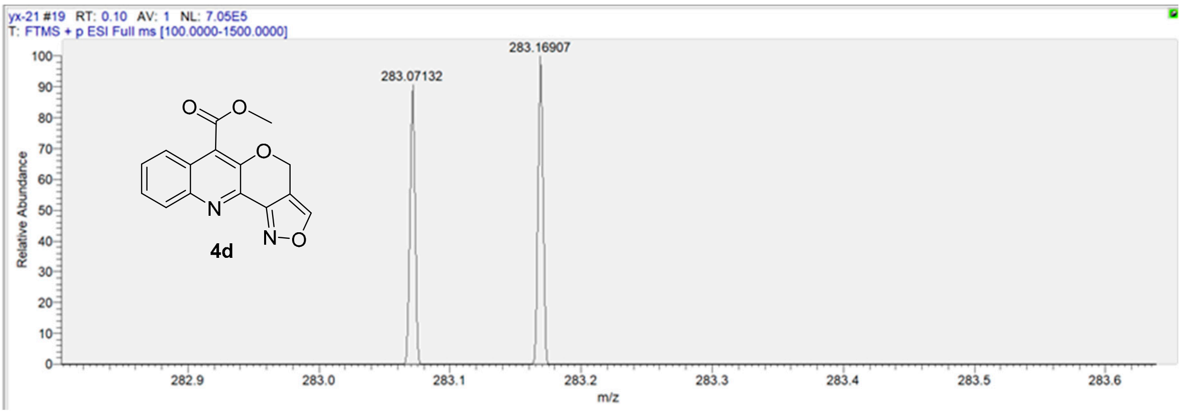

yx-21 #19 RT: 0.10 AV: 1 NL: 7.05E5
T: FTMS + p ESI Full ms [100.0000-1500.0000]
283.16907
283.07132
4d
Relative Abundance
m/z

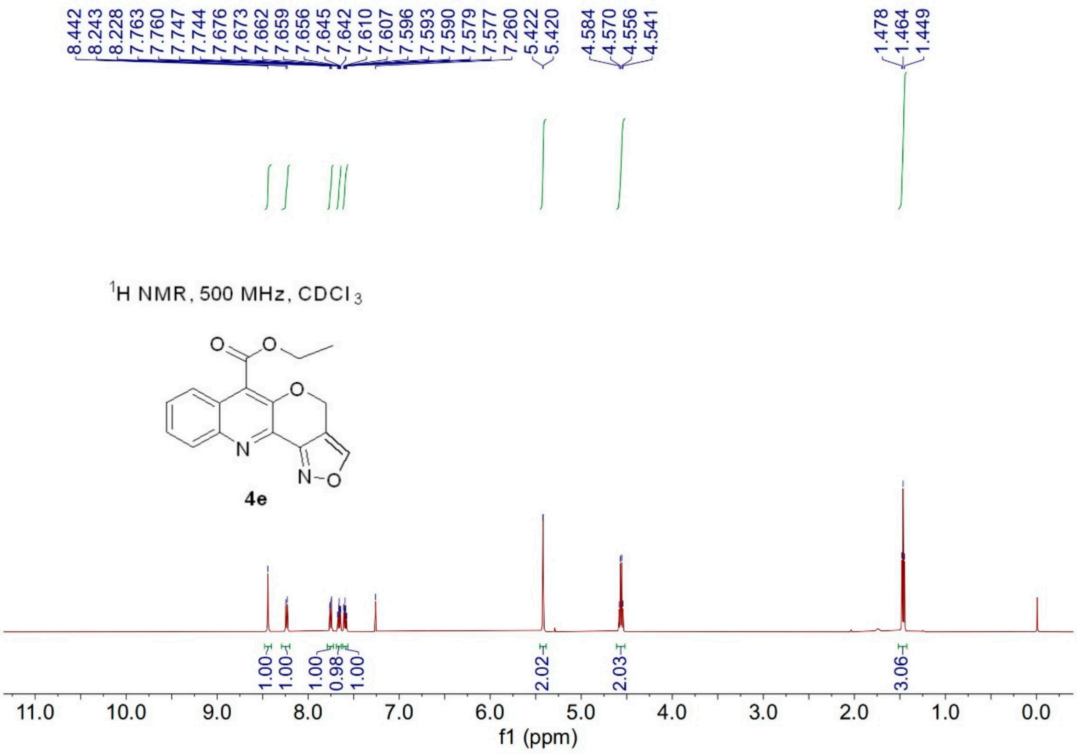
8.442
8.243
8.228
7.763
7.760
7.747
7.744
7.676
7.673
7.662
7.659
7.656
7.645
7.642
7.610
7.607
7.596
7.593
7.590
7.579
7.577
7.260
5.422
5.420
4.584
4.570
4.556
4.541
1.478
1.464
1.449
1H NMR, 500 MHz, CDCl3
4e
1.00
1.00
1.00
0.98
1.00
2.02
2.03
3.06
f1 (ppm)

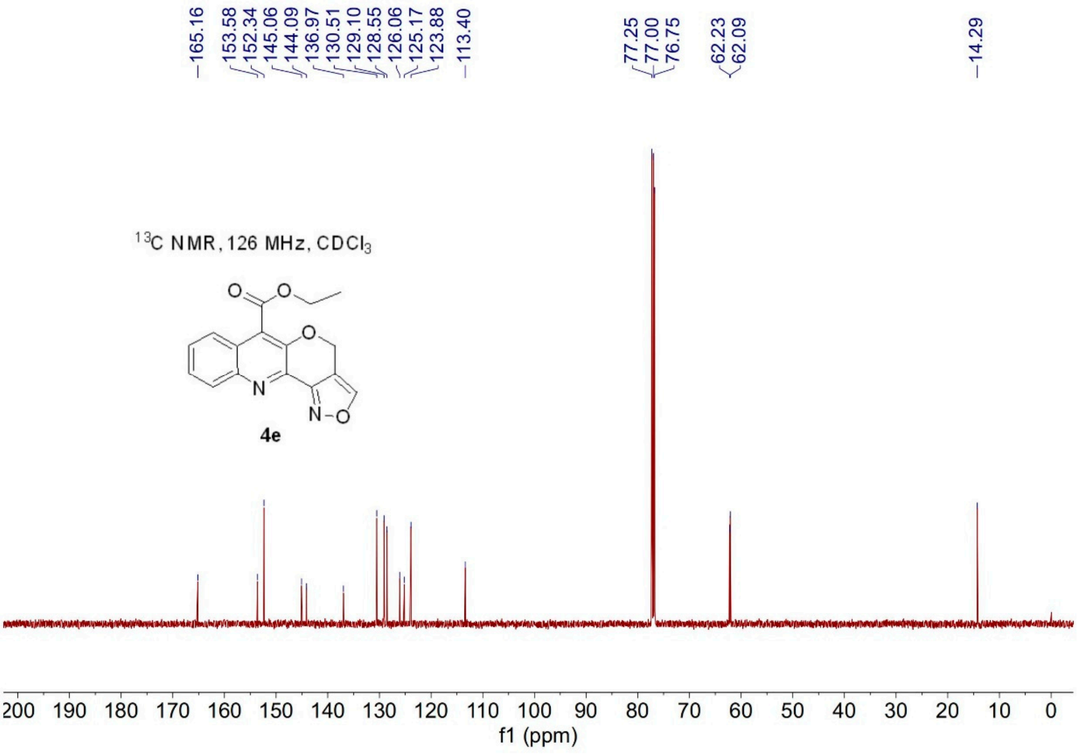
165.16
153.58
152.34
145.06
144.09
136.97
130.51
129.10
128.55
126.06
125.17
123.88
113.40
77.25
77.00
76.75
62.23
62.09
14.29
13C NMR, 126 MHz, CDCl3
4e
f1 (ppm)

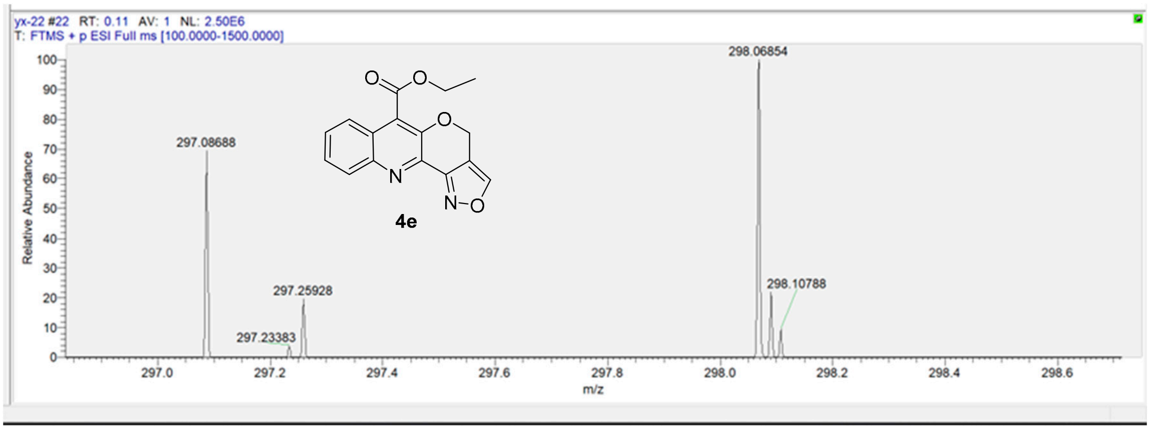
yx-22 #22 RT: 0.11 AV: 1 NL: 2.50E6
T: FTMS + p ESI Full ms [100.0000-1500.0000]
298.06854
297.08688
297.25928
297.23383
298.10788
Relative Abundance
m/z
4e

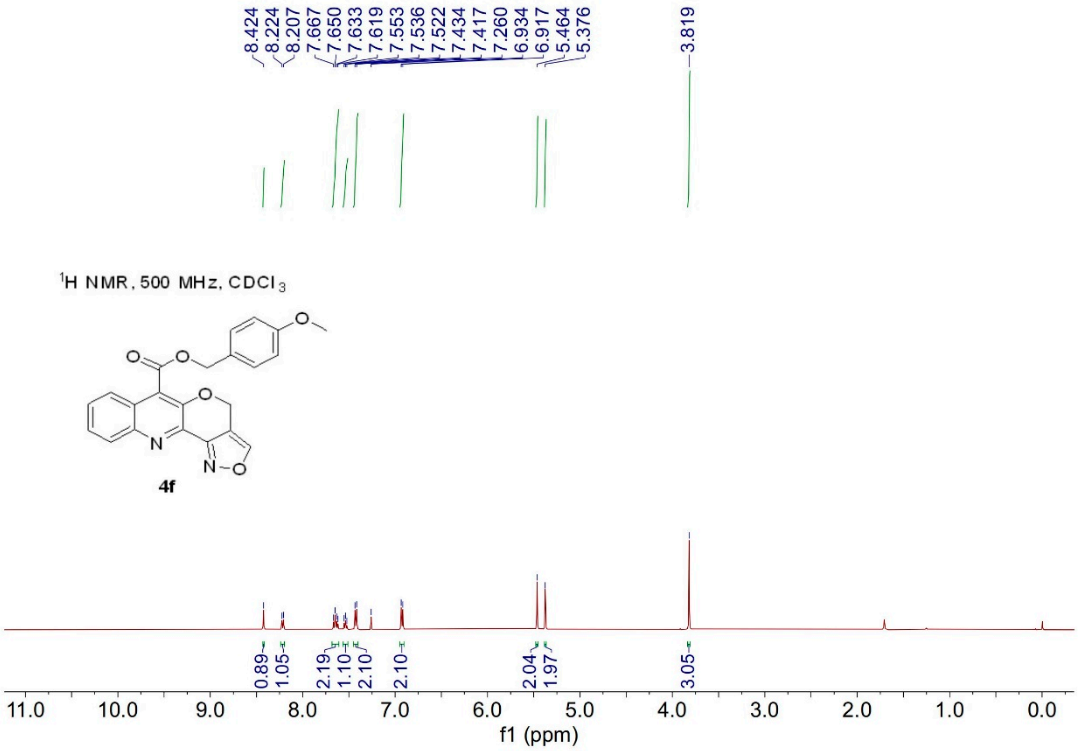
8.424
8.224
8.207
7.667
7.650
7.633
7.619
7.553
7.536
7.522
7.434
7.417
7.260
6.934
6.917
5.464
5.376
3.819
^{1}H NMR, 500 MHz, CDCl$_3$
4f
0.89
1.05
2.19
1.10
2.10
2.10
2.04
1.97
3.05
f1 (ppm)

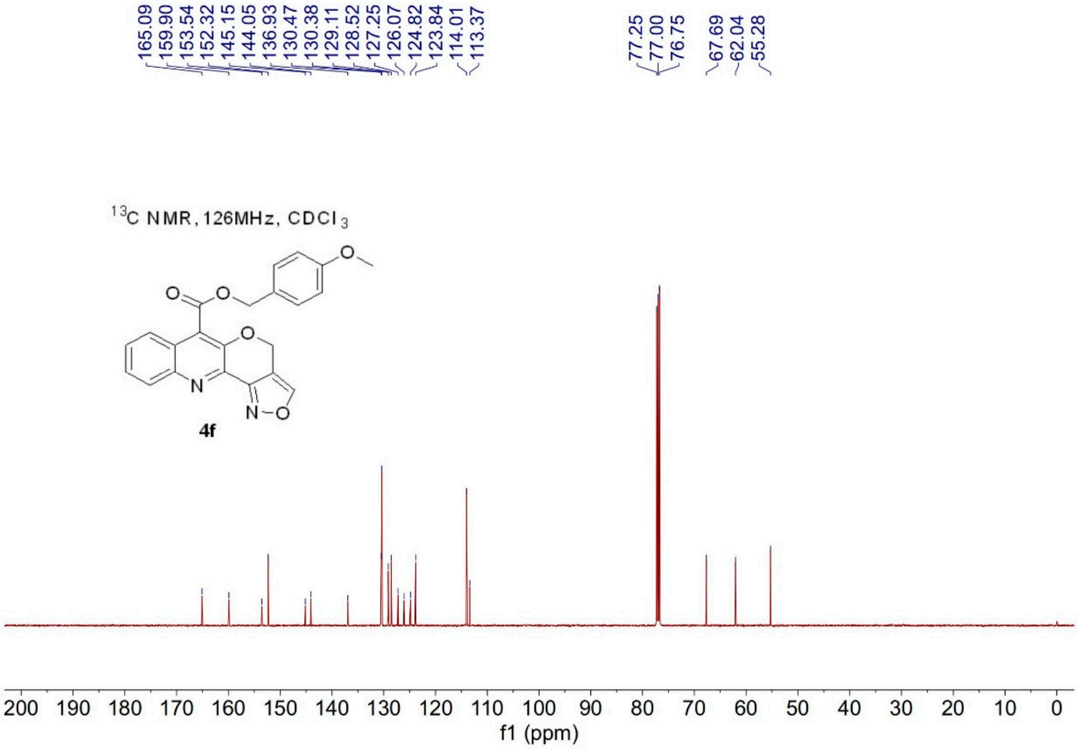
165.09
159.90
153.54
152.32
145.15
144.05
136.93
130.47
130.38
129.11
128.52
127.25
126.07
124.82
123.84
114.01
113.37
77.25
77.00
76.75
67.69
62.04
55.28
^{13}C NMR, 126MHz, $CDCl_3$
4f
f1 (ppm)

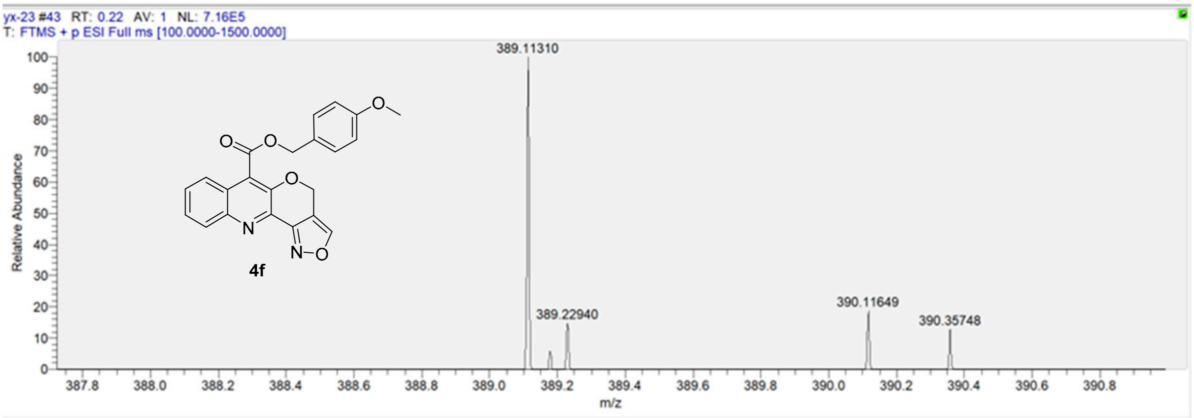
yx-23 #43 RT: 0.22 AV: 1 NL: 7.16E5
T: FTMS + p ESI Full ms [100.0000-1500.0000]
389.11310
389.22940
390.11649
390.35748
4f
Relative Abundance
m/z

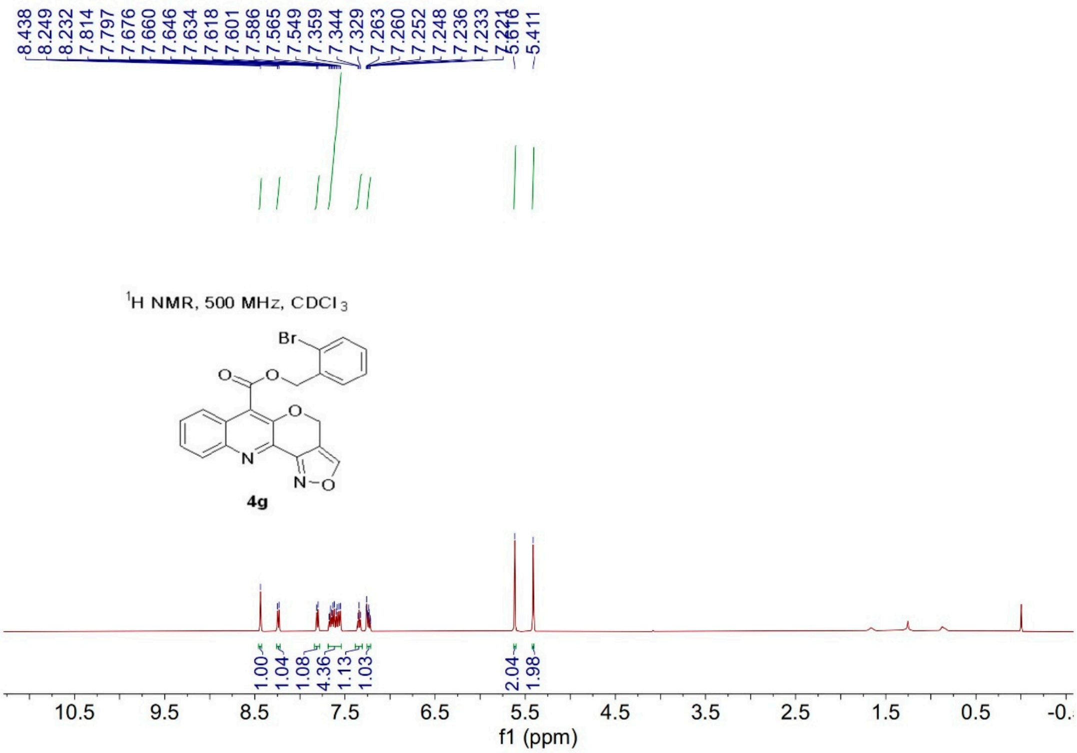
8.438
8.249
8.232
7.814
7.797
7.676
7.660
7.646
7.634
7.618
7.601
7.586
7.565
7.549
7.359
7.344
7.329
7.263
7.260
7.252
7.248
7.236
7.233
7.221
5.516
5.411
1H NMR, 500 MHz, CDCl3
Br
O
O
O
N
N–O
4g
1.00
1.04
1.08
4.36
1.13
1.03
2.04
1.98
10.5 9.5 8.5 7.5 6.5 5.5 4.5 3.5 2.5 1.5 0.5
f1 (ppm)

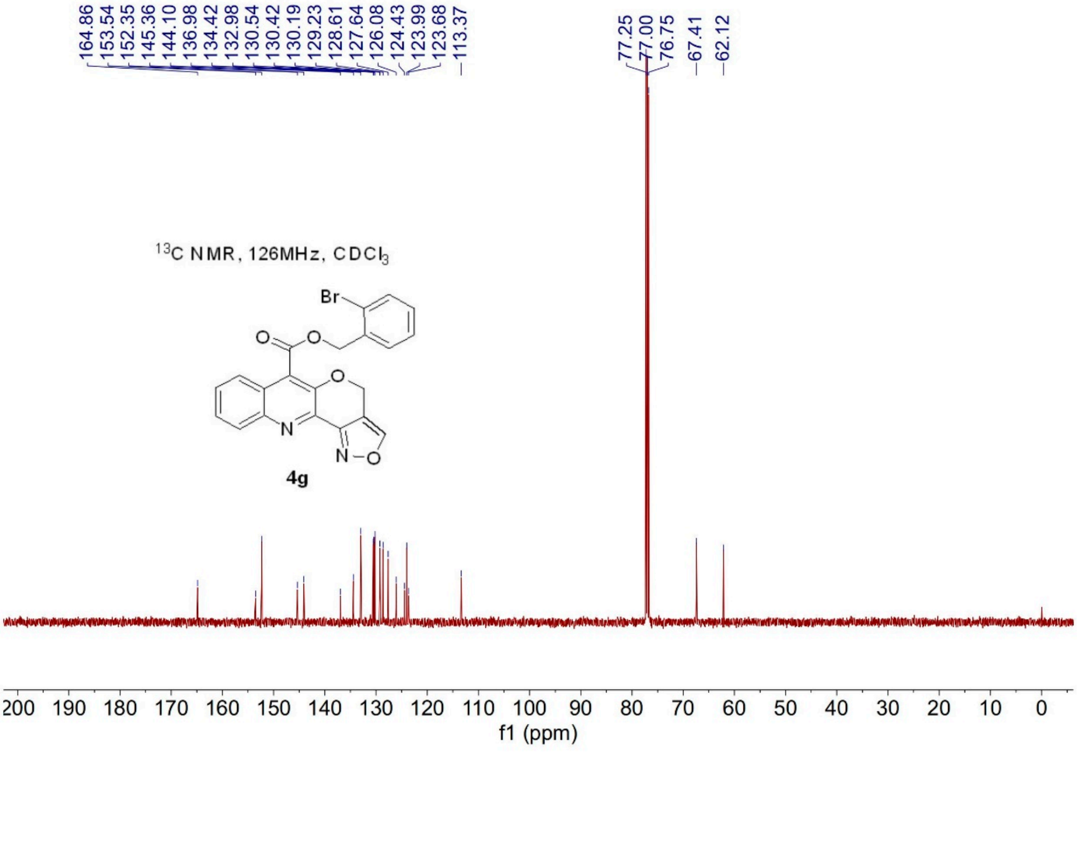
164.86
153.54
152.35
145.36
144.10
136.98
134.42
132.98
130.54
130.42
130.19
129.23
128.61
127.64
126.08
124.43
123.99
123.68
113.37
77.25
77.00
76.75
67.41
62.12
13C NMR, 126MHz, CDCl3
Br
O
O
O
N
N–O
4g
200 190 180 170 160 150 140 130 120 110 100 90 80 70 60 50 40 30 20 10 0
f1 (ppm)

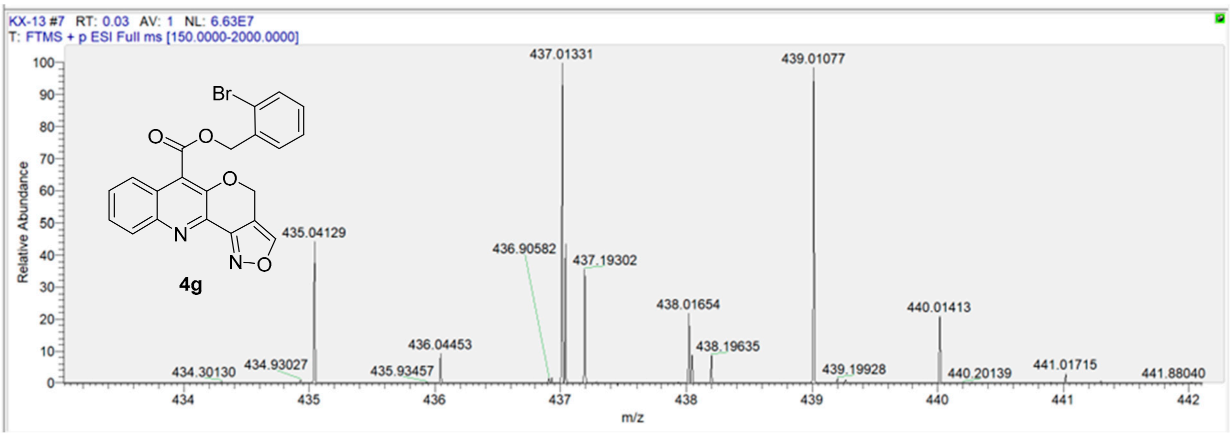
KX-13 #7 RT: 0.03 AV: 1 NL: 6.63E7
T: FTMS + p ESI Full ms [150.0000-2000.0000]
Relative Abundance
m/z
437.01331
439.01077
435.04129
436.90582
437.19302
438.01654
440.01413
436.04453
438.19635
434.30130
434.93027
435.93457
439.19928
440.20139
441.01715
441.88040
Br
4g

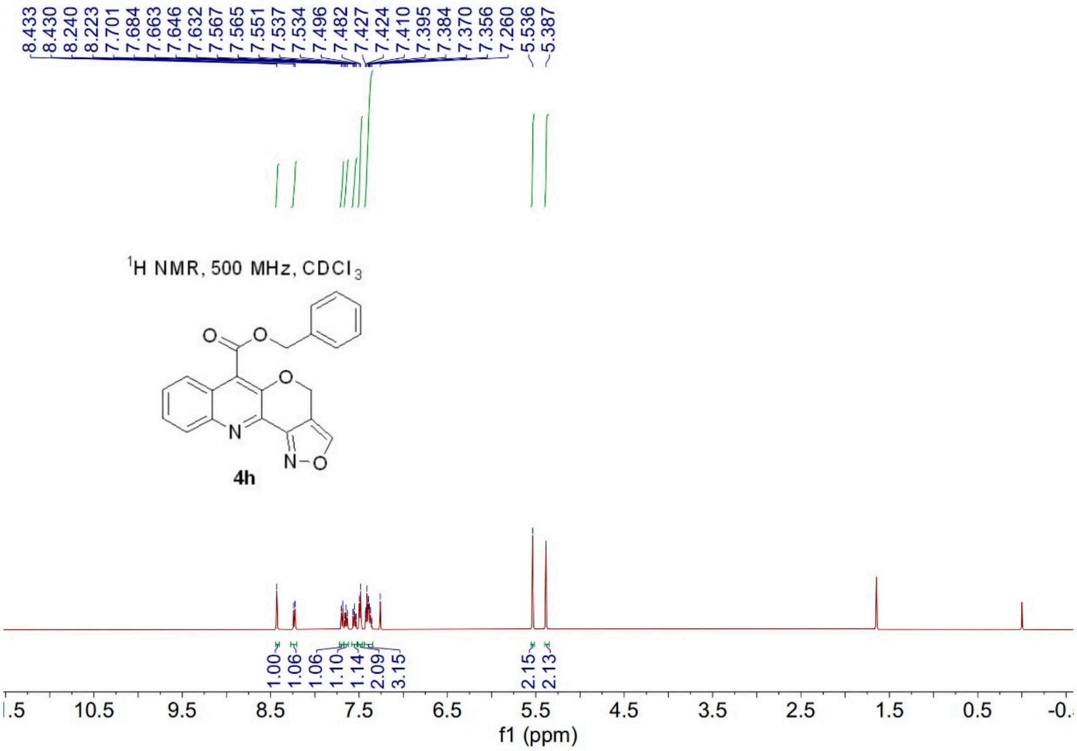
8.433
8.430
8.240
8.223
7.701
7.684
7.663
7.646
7.632
7.567
7.565
7.551
7.537
7.534
7.496
7.482
7.427
7.424
7.410
7.395
7.384
7.370
7.356
7.260
5.536
5.387
1H NMR, 500 MHz, CDCl3
4h
1.00
1.06
1.06
1.10
1.14
2.09
3.15
2.15
2.13
f1 (ppm)

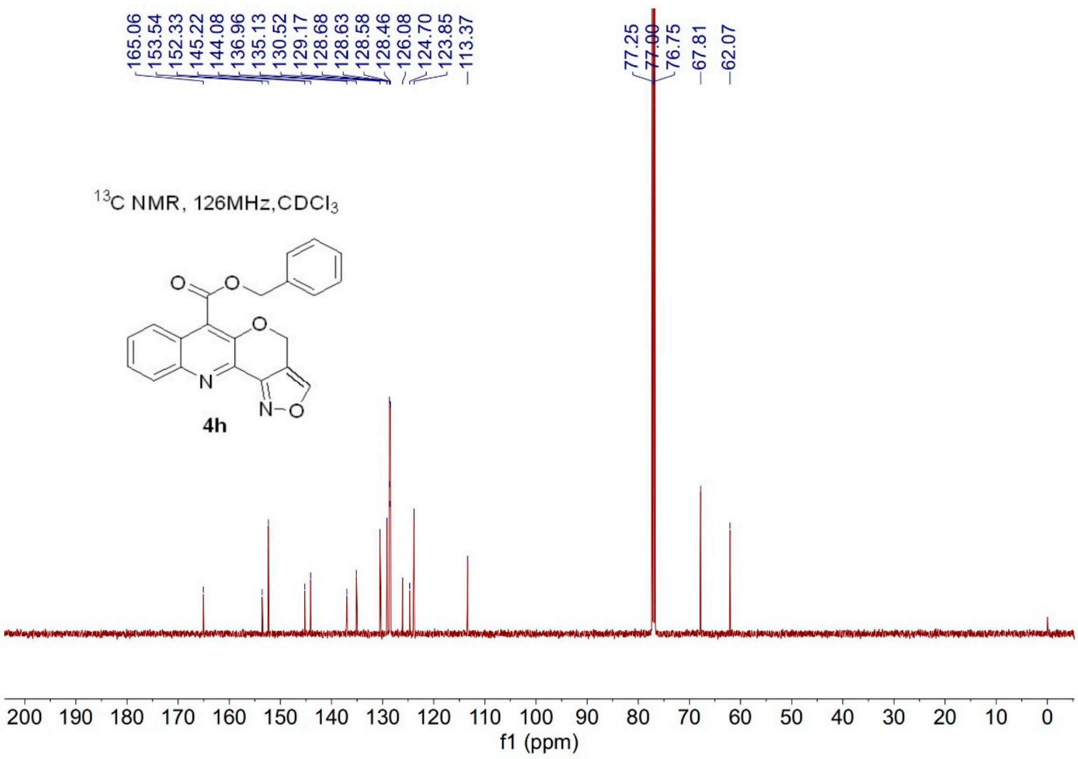
165.06
153.54
152.33
145.22
144.08
136.96
135.13
130.52
129.17
128.68
128.63
128.58
128.46
126.08
124.70
123.85
113.37
77.25
77.00
76.75
67.81
62.07
^{13}C NMR, 126MHz,CDCl$_3$
O
O
O
N
N-O
4h
200 190 180 170 160 150 140 130 120 110 100 90 80 70 60 50 40 30 20 10 0
f1 (ppm)

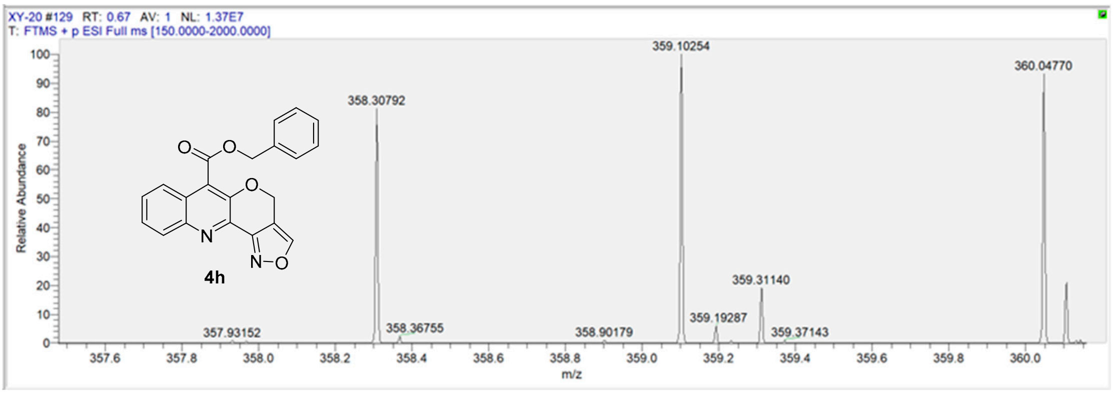
XY-20 #129 RT: 0.67 AV: 1 NL: 1.37E7
T: FTMS + p ESI Full ms [150.0000-2000.0000]
Relative Abundance
359.10254
360.04770
358.30792
359.31140
359.19287
357.93152
358.36755
358.90179
359.37143
4h
m/z

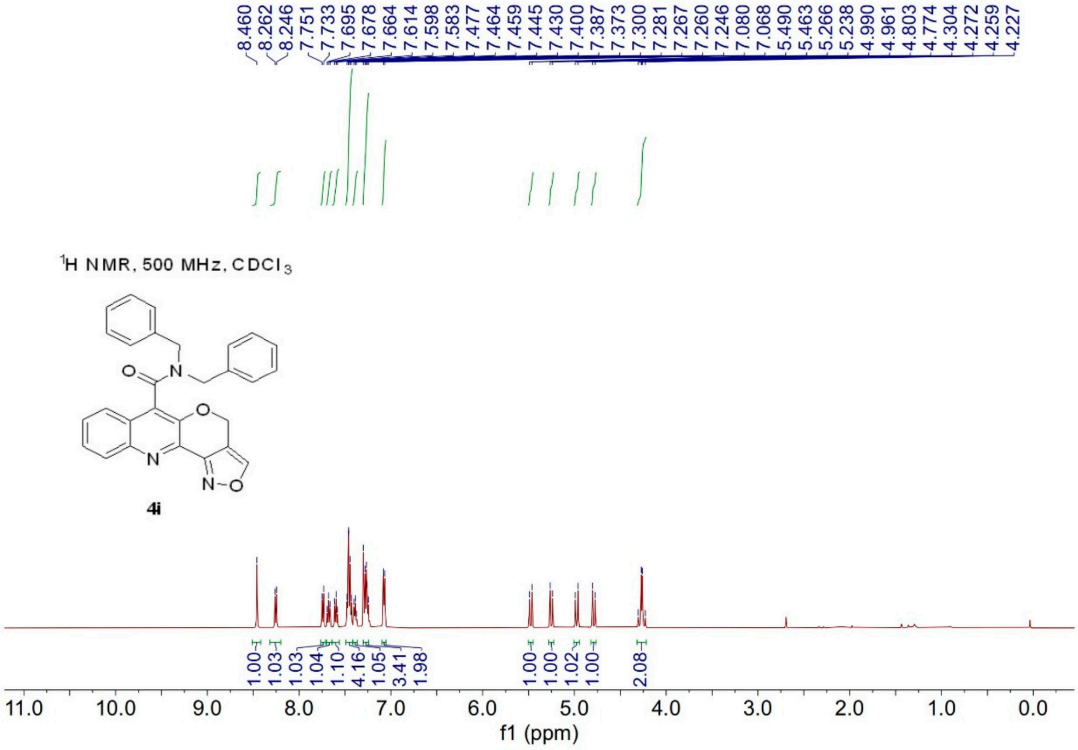
8.460 8.262 8.246 7.751 7.733 7.695 7.678 7.664 7.614 7.598 7.583 7.477 7.464 7.459 7.445 7.430 7.400 7.387 7.373 7.300 7.281 7.267 7.260 7.246 7.080 7.068 5.490 5.463 5.266 5.238 4.990 4.961 4.803 4.774 4.304 4.272 4.259 4.227
1H NMR, 500 MHz, CDCl3
4i
1.00 1.03 1.03 1.04 1.10 4.16 1.05 3.41 1.98 1.00 1.00 1.02 1.00 2.08
11.0 10.0 9.0 8.0 7.0 6.0 5.0 4.0 3.0 2.0 1.0 0.0
f1 (ppm)

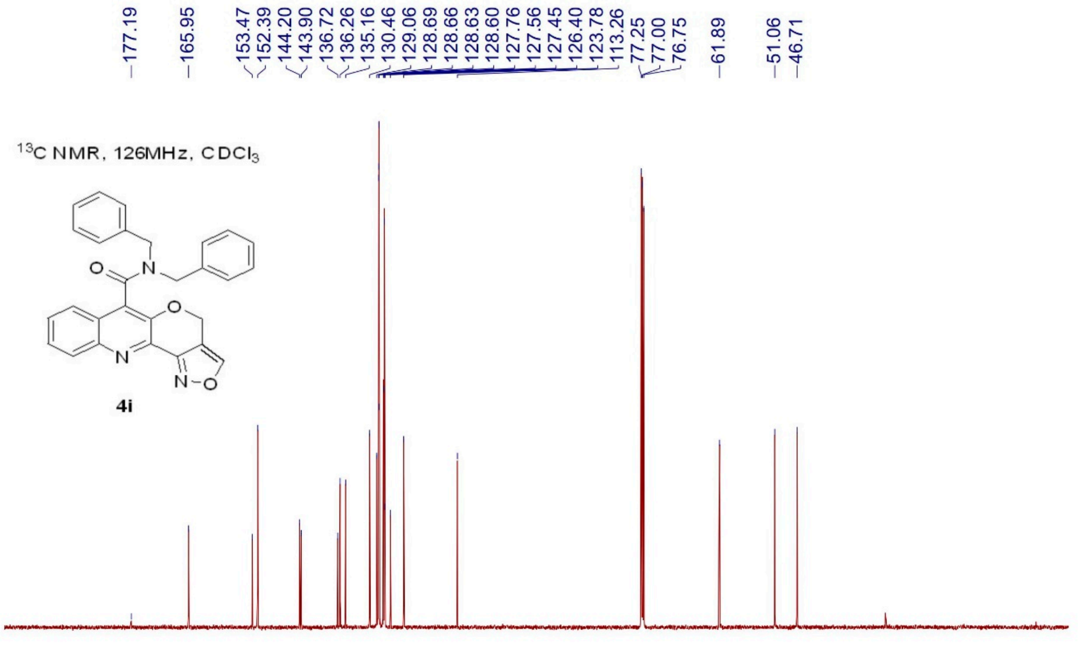
177.19 165.95 153.47 152.39 144.20 143.90 136.72 136.26 135.16 130.46 129.06 128.69 128.66 128.63 128.60 127.76 127.56 127.45 126.40 123.78 113.26 77.25 77.00 76.75 61.89 51.06 46.71
13C NMR, 126MHz, CDCl3
4i
200 190 180 170 160 150 140 130 120 110 100 90 80 70 60 50 40 30 20 10 0
f1 (ppm)

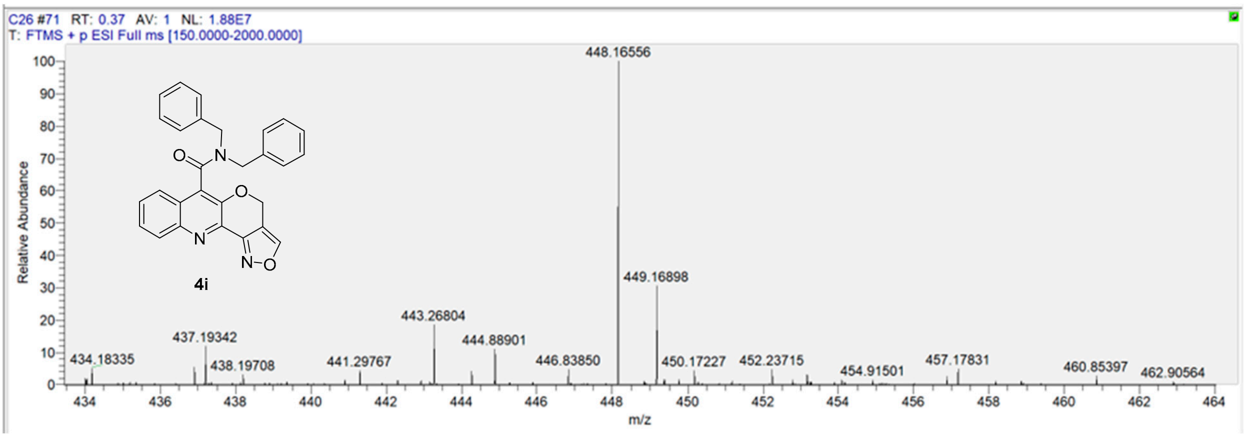
C26 #71 RT: 0.37 AV: 1 NL: 1.88E7
T: FTMS + p ESI Full ms [150.0000-2000.0000]
448.16556
449.16898
443.26804
444.88901
437.19342
434.18335
438.19708
441.29767
446.83850
450.17227
452.23715
454.91501
457.17831
460.85397
462.90564
Relative Abundance
m/z
4i

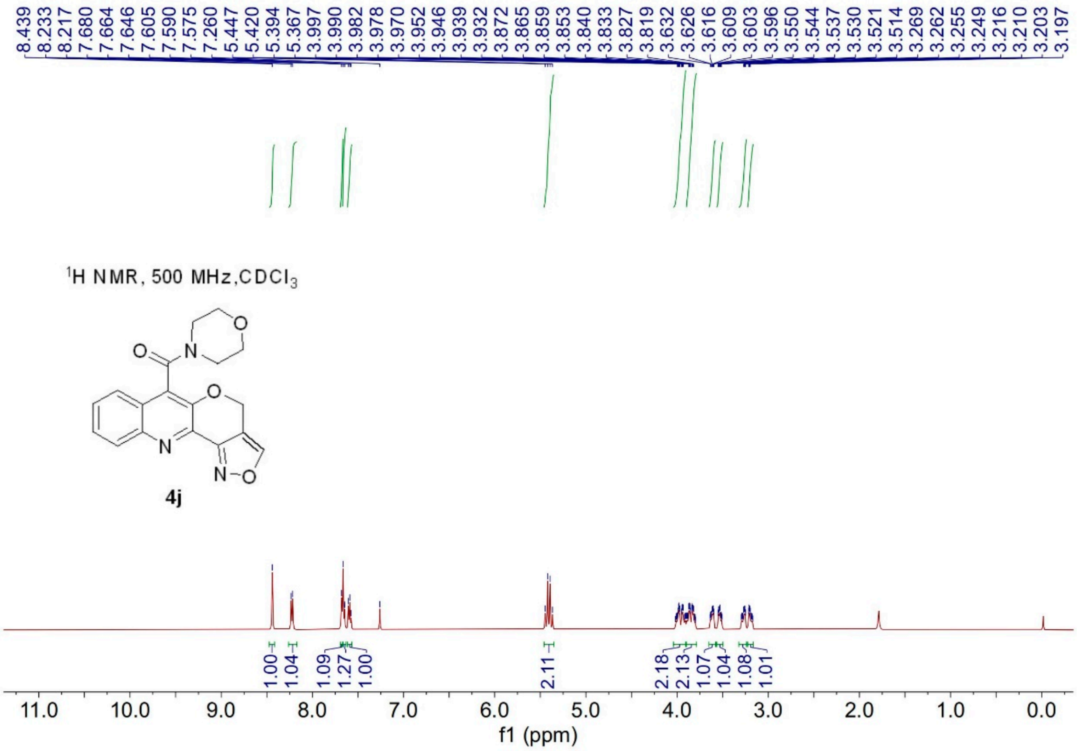
8.439
8.233
8.217
7.680
7.664
7.646
7.605
7.590
7.575
7.260
5.447
5.420
5.394
5.367
3.997
3.990
3.982
3.978
3.970
3.952
3.946
3.939
3.932
3.872
3.865
3.859
3.853
3.840
3.833
3.827
3.819
3.632
3.626
3.616
3.609
3.603
3.596
3.550
3.544
3.537
3.530
3.521
3.514
3.269
3.262
3.255
3.249
3.216
3.210
3.203
3.197
^{1}H NMR, 500 MHz,CDCl$_3$
4j
1.00
1.04
1.09
1.27
1.00
2.11
2.18
2.13
1.07
1.04
1.08
1.01
f1 (ppm)

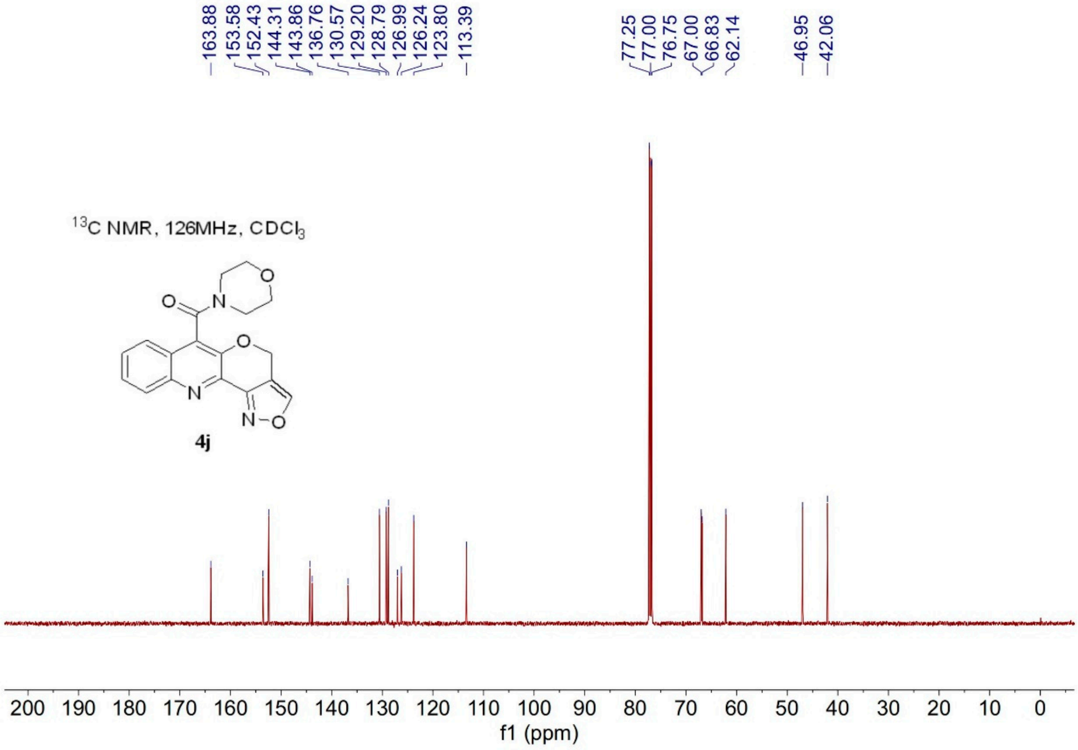
163.88
153.58
152.43
144.31
143.86
136.76
130.57
129.20
128.79
126.99
126.24
123.80
113.39
77.25
77.00
76.75
67.00
66.83
62.14
46.95
42.06
^{13}C NMR, 126MHz, CDCl$_3$
4j
f1 (ppm)

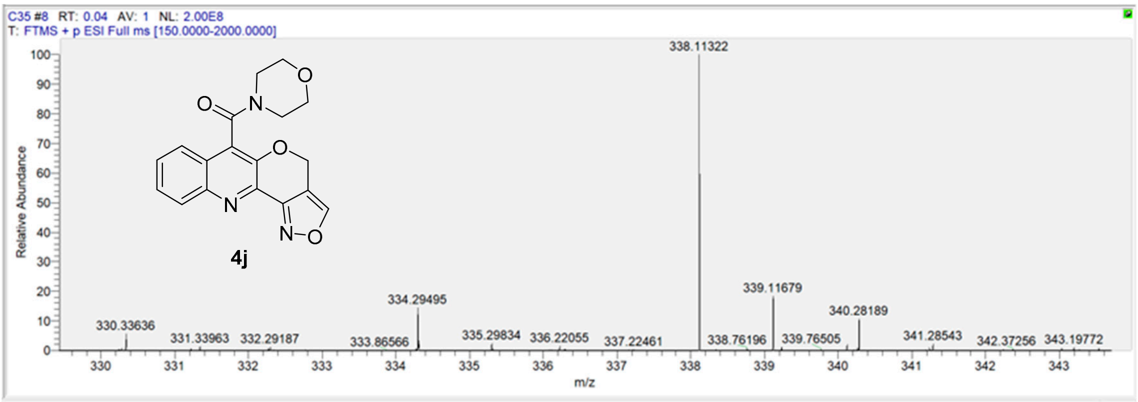
C35 #8 RT: 0.04 AV: 1 NL: 2.00E8
T: FTMS + p ESI Full ms [150.0000-2000.0000]
338.11322
339.11679
340.28189
334.29495
330.33636
331.33963
332.29187
333.86566
335.29834
336.22055
337.22461
338.76196
339.76505
341.28543
342.37256
343.19772
Relative Abundance
4j
m/z

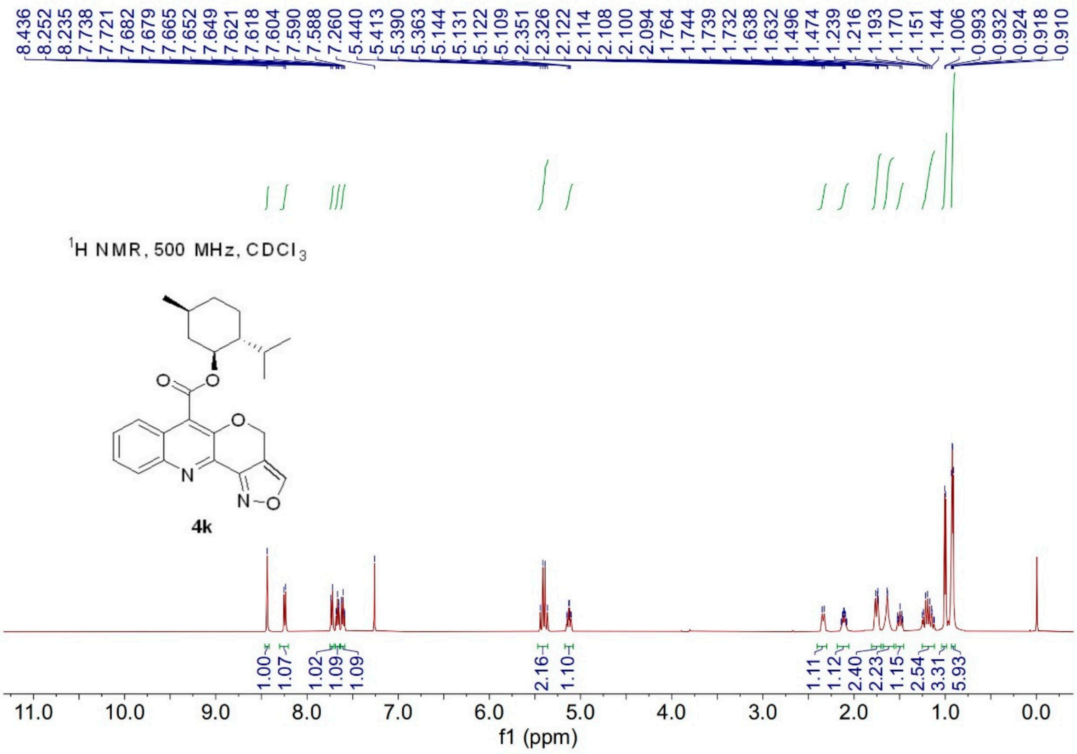
8.436
8.252
8.235
7.738
7.721
7.682
7.679
7.665
7.652
7.649
7.621
7.618
7.604
7.590
7.588
7.260
5.440
5.413
5.390
5.363
5.144
5.131
5.122
5.109
2.351
2.326
2.122
2.114
2.108
2.100
2.094
1.764
1.744
1.739
1.732
1.638
1.632
1.496
1.474
1.239
1.216
1.193
1.170
1.151
1.144
1.006
0.993
0.932
0.924
0.918
0.910
^{1}H NMR, 500 MHz, CDCl$_3$
N
O
O
N-O
4k
1.00
1.07
1.02
1.09
1.09
2.16
1.10
1.11
1.12
2.40
2.23
1.15
2.54
3.31
5.93
11.0
10.0
9.0
8.0
7.0
6.0
5.0
4.0
3.0
2.0
1.0
0.0
f1 (ppm)

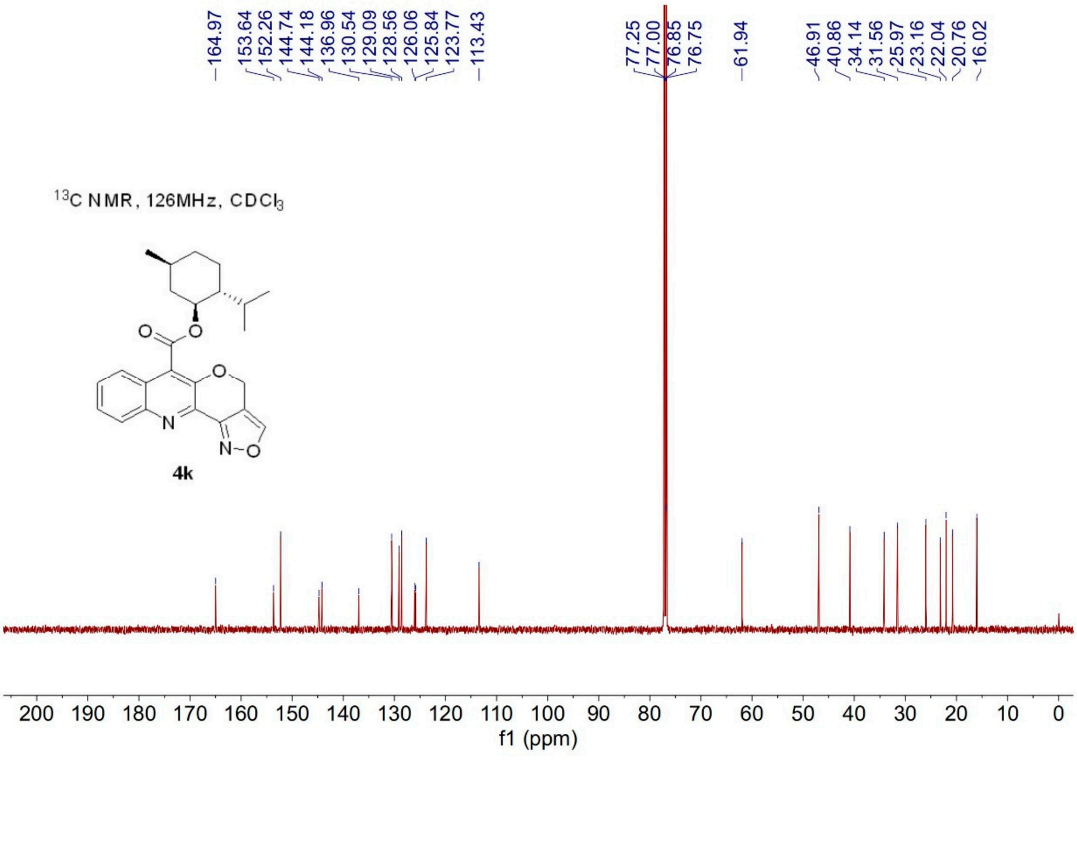
164.97
153.64
152.26
144.74
144.18
136.96
130.54
129.09
128.56
126.06
125.84
123.77
113.43
77.25
77.00
76.85
76.75
61.94
46.91
40.86
34.14
31.56
25.97
23.16
22.04
20.76
16.02
^{13}C NMR, 126MHz, CDCl$_3$
N
O
O
N-O
4k
200
190
180
170
160
150
140
130
120
110
100
90
80
70
60
50
40
30
20
10
0
f1 (ppm)

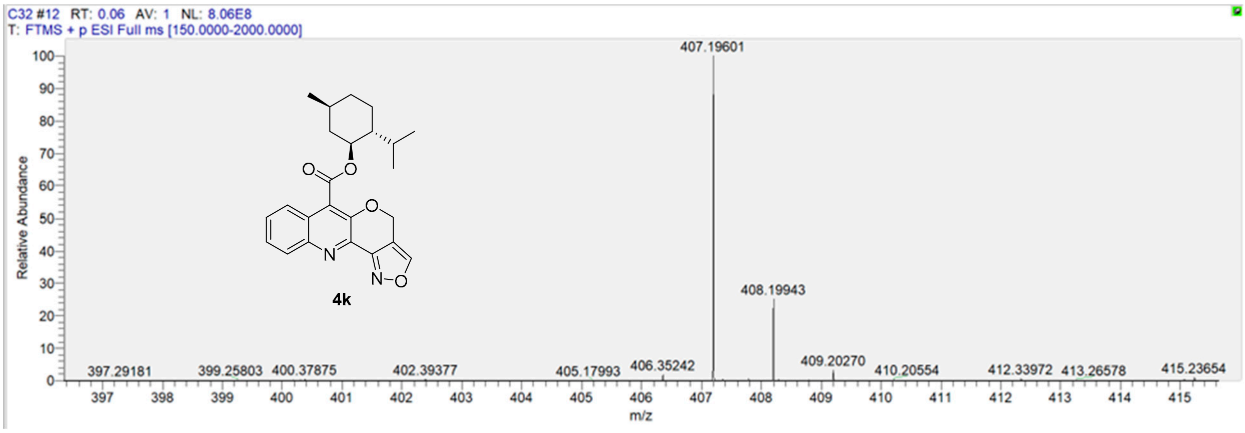

C32 #12 RT: 0.06 AV: 1 NL: 8.06E8
T: FTMS + p ESI Full ms [150.0000-2000.0000]
407.19601
408.19943
409.20270
Relative Abundance
m/z
4k

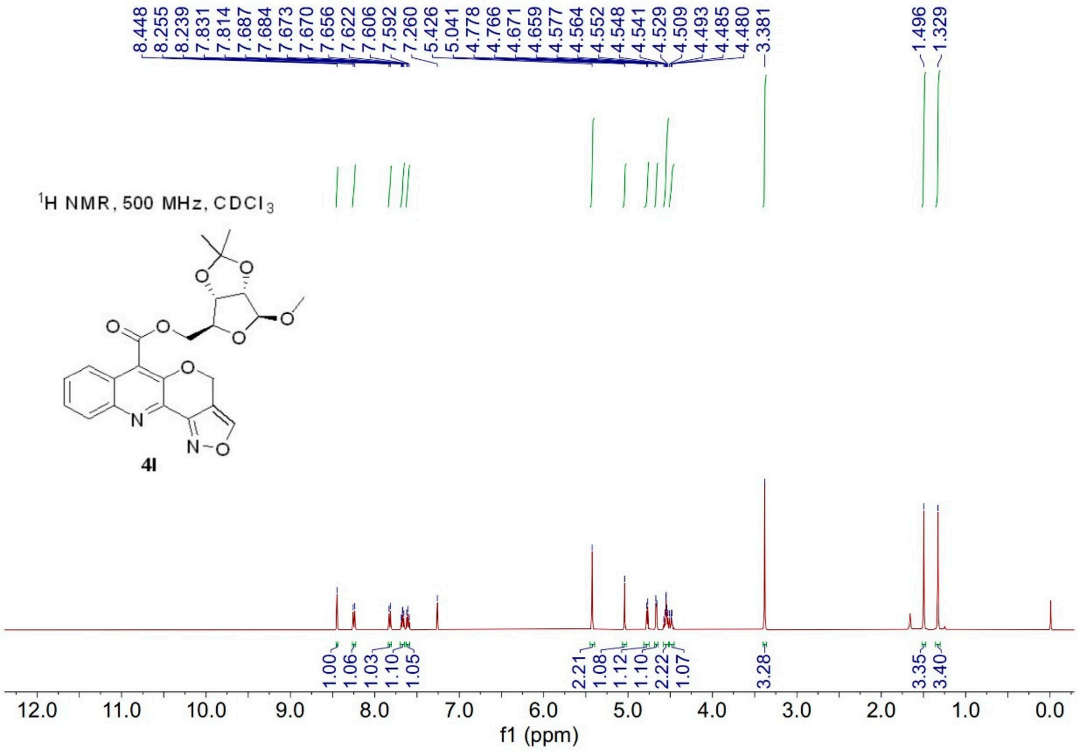

^{1}H NMR, 500 MHz, CDCl$_3$
4l
f1 (ppm)

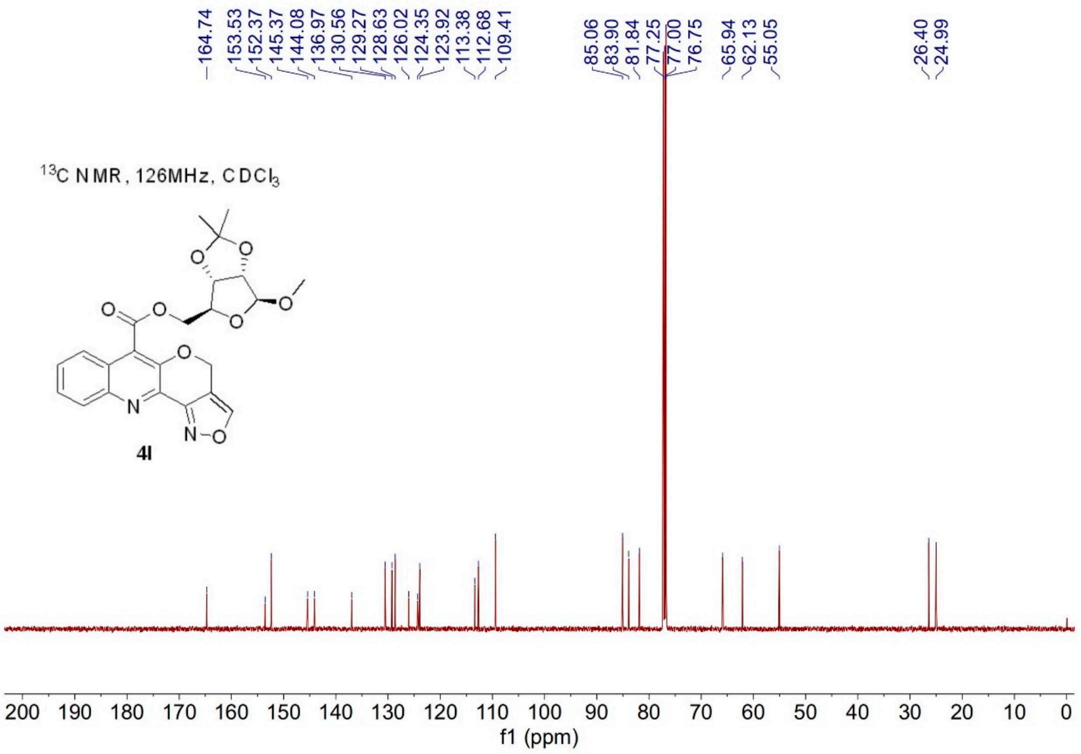
164.74
153.53
152.37
145.37
144.08
136.97
130.56
129.27
128.63
126.02
124.35
123.92
113.38
112.68
109.41
85.06
83.90
81.84
77.25
77.00
76.75
65.94
62.13
55.05
26.40
24.99
^{13}C NMR, 126MHz, CDCl$_3$
4l
200 190 180 170 160 150 140 130 120 110 100 90 80 70 60 50 40 30 20 10 0
f1 (ppm)

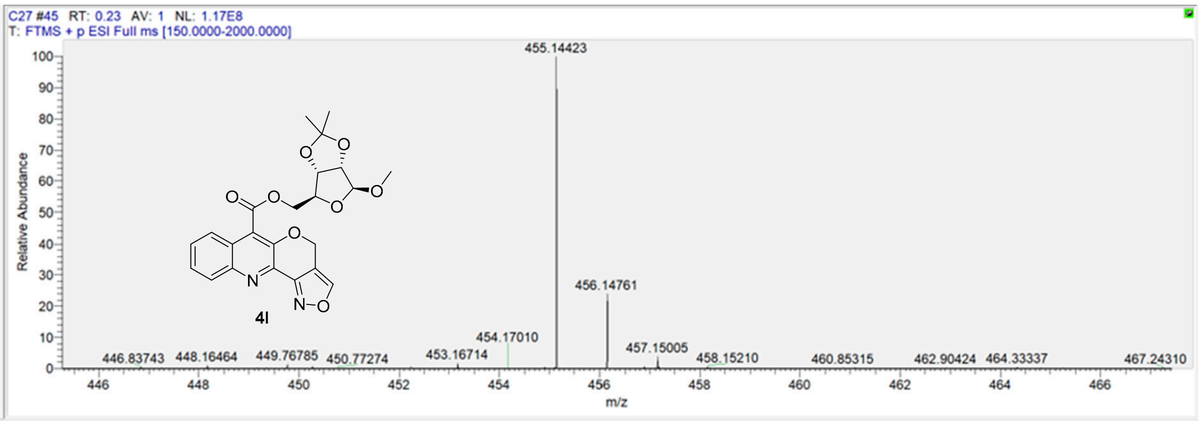
C27 #45 RT: 0.23 AV: 1 NL: 1.17E8
T: FTMS + p ESI Full ms [150.0000-2000.0000]
455.14423
456.14761
454.17010
453.16714
457.15005
446.83743
448.16464
449.76785
450.77274
458.15210
460.85315
462.90424
464.33337
467.24310
Relative Abundance
m/z
4l

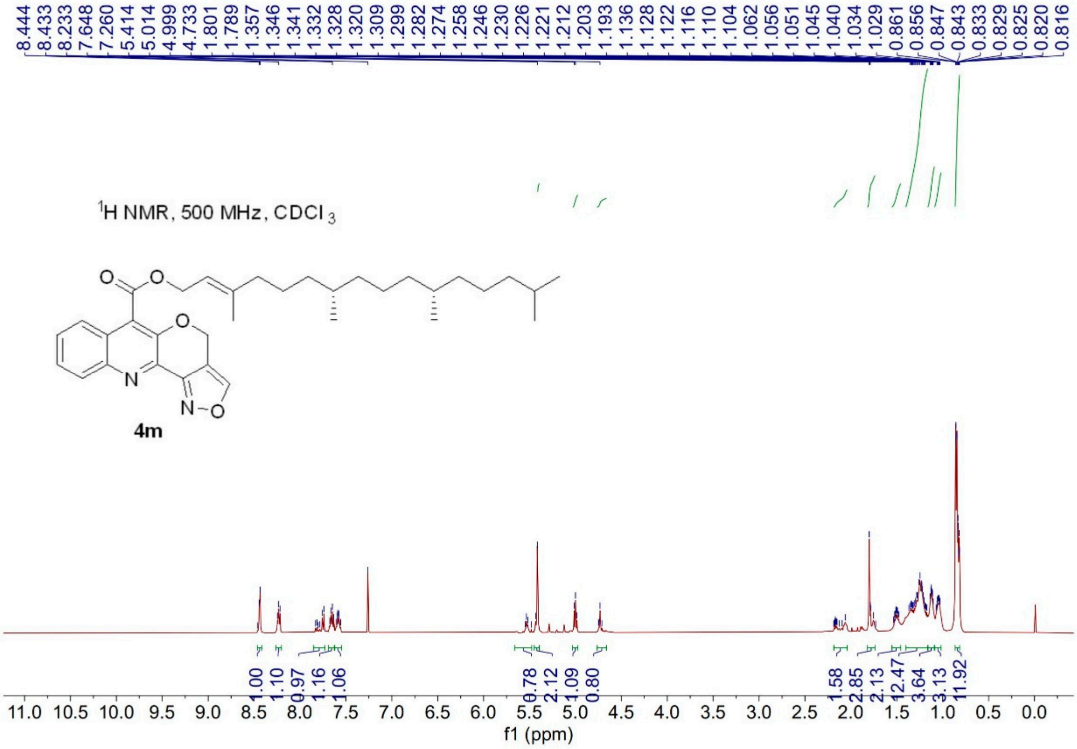

^{1}H NMR, 500 MHz, CDCl $_3$
O
N
N-O
4m
f1 (ppm)

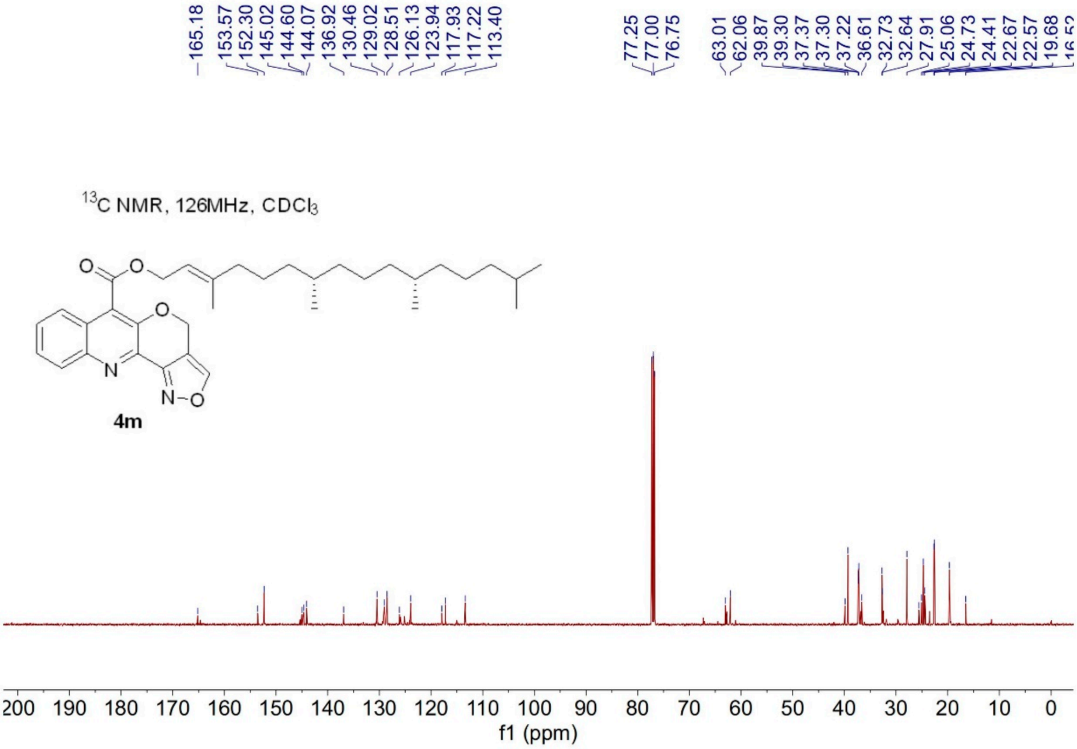

^{13}C NMR, 126MHz, CDCl$_3$
O
N
N-O
4m
f1 (ppm)

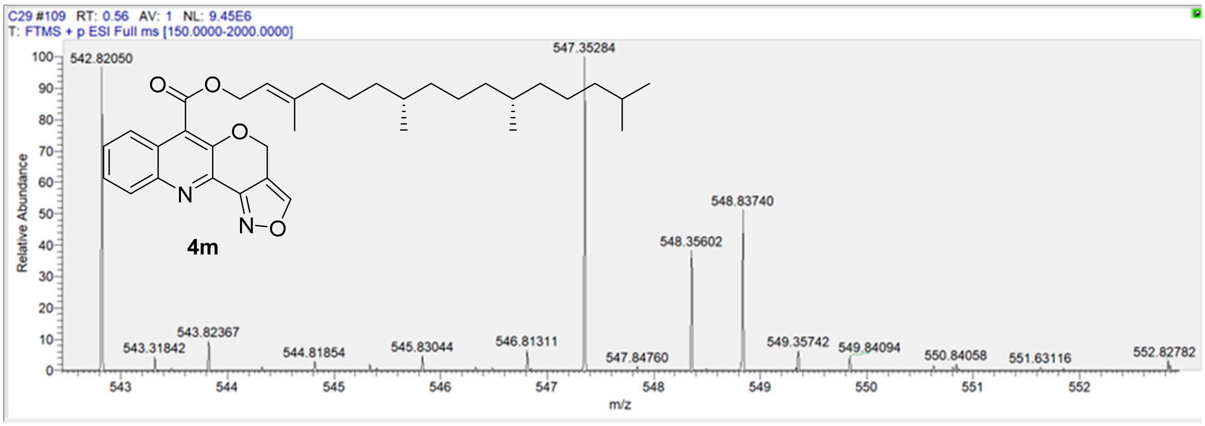
C29 #109 RT: 0.56 AV: 1 NL: 9.45E6
T: FTMS + p ESI Full ms [150.0000-2000.0000]
Relative Abundance
m/z
542.82050
543.31842
543.82367
544.81854
545.83044
546.81311
547.35284
547.84760
548.35602
548.83740
549.35742
549.84094
550.84058
551.63116
552.82782
4m

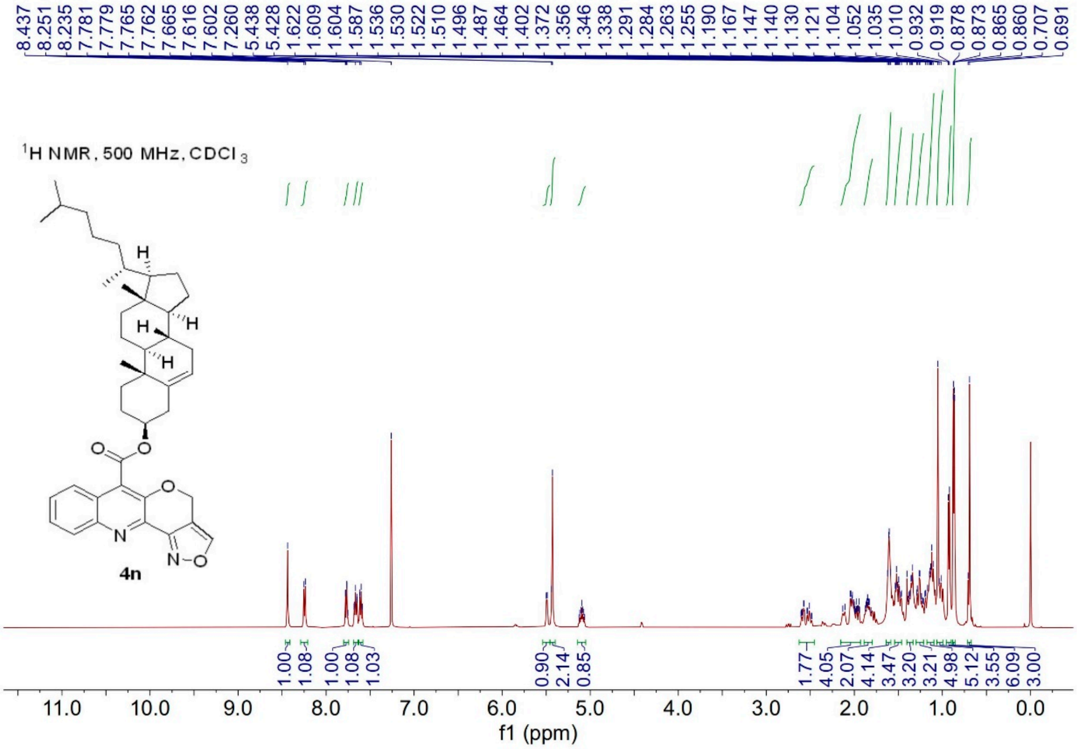
8.437 8.251 8.235 7.781 7.779 7.765 7.762 7.665 7.616 7.602 7.260 5.438 5.428 1.622 1.609 1.604 1.587 1.536 1.530 1.522 1.510 1.496 1.487 1.464 1.402 1.372 1.356 1.346 1.338 1.291 1.284 1.263 1.255 1.190 1.167 1.147 1.140 1.130 1.121 1.104 1.052 1.035 1.010 0.932 0.919 0.878 0.873 0.865 0.860 0.707 0.691
^{1}H NMR, 500 MHz, CDCl$_3$
4n
1.00 1.08 1.00 1.08 1.03 0.90 2.14 0.85 1.77 4.05 2.07 4.14 3.47 3.20 3.21 4.98 5.12 3.55 6.09 3.00
f1 (ppm)

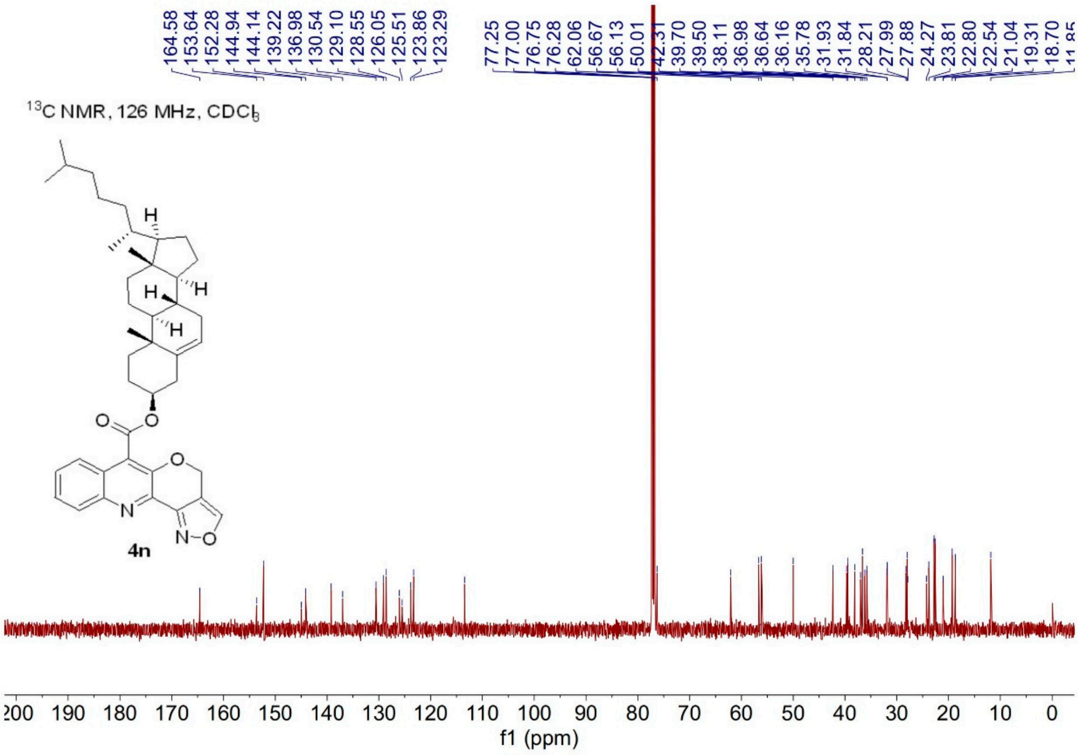
164.58
153.64
152.28
144.94
144.14
139.22
136.98
130.54
129.10
128.55
126.05
125.51
123.86
123.29
77.25
77.00
76.75
76.28
62.06
56.67
56.13
50.01
42.31
39.70
39.50
38.11
36.98
36.64
36.16
35.78
31.93
31.84
28.21
27.99
27.88
24.27
23.81
22.80
22.54
21.04
19.31
18.70
11.85
^{13}C NMR, 126 MHz, CDCl$_3$
4n
200 190 180 170 160 150 140 130 120 110 100 90 80 70 60 50 40 30 20 10 0
f1 (ppm)

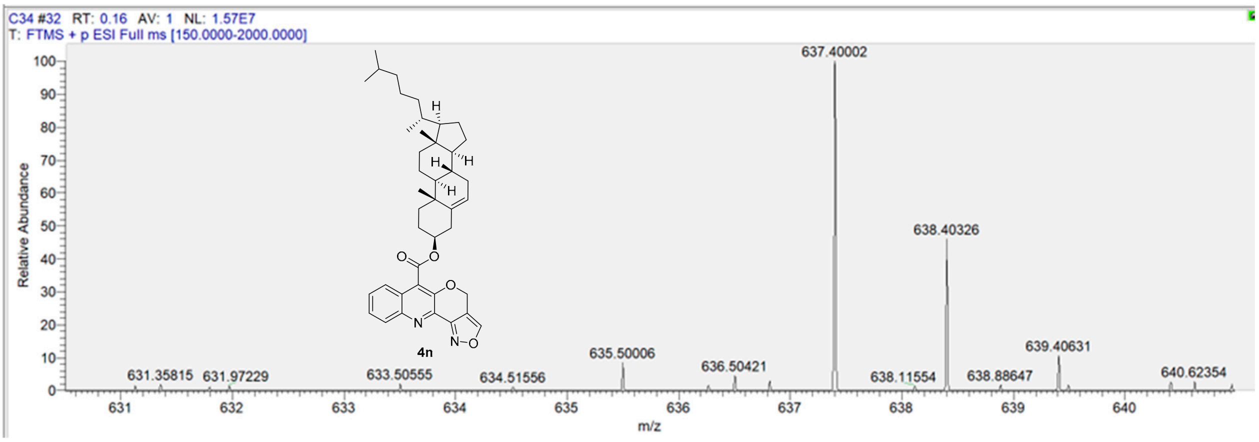
C34 #32 RT: 0.16 AV: 1 NL: 1.57E7
T: FTMS + p ESI Full ms [150.0000-2000.0000]
Relative Abundance
637.40002
638.40326
639.40631
640.62354
635.50006
636.50421
631.35815
631.97229
633.50555
634.51556
638.11554
638.88647
4n
m/z

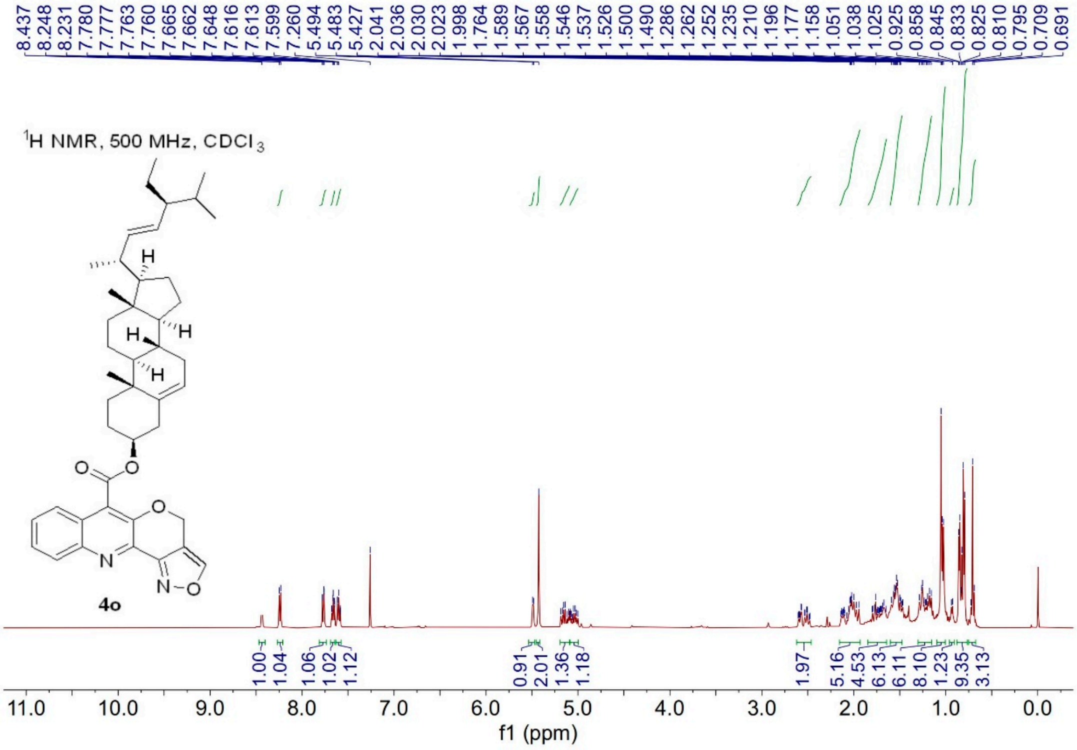
8.437 8.248 8.231 7.780 7.777 7.763 7.760 7.665 7.662 7.648 7.616 7.613 7.599 7.260 5.494 5.483 5.427 2.041 2.036 2.030 2.023 1.998 1.764 1.589 1.567 1.558 1.546 1.537 1.526 1.500 1.490 1.286 1.262 1.252 1.235 1.210 1.196 1.177 1.158 1.051 1.038 1.025 0.925 0.858 0.845 0.833 0.825 0.810 0.795 0.709 0.691
1H NMR, 500 MHz, CDCl3
4o
1.00 1.04 1.06 1.02 1.12 0.91 2.01 1.36 1.18 1.97 5.16 4.53 6.13 6.11 8.10 1.23 9.35 3.13
11.0 10.0 9.0 8.0 7.0 6.0 5.0 4.0 3.0 2.0 1.0 0.0
f1 (ppm)

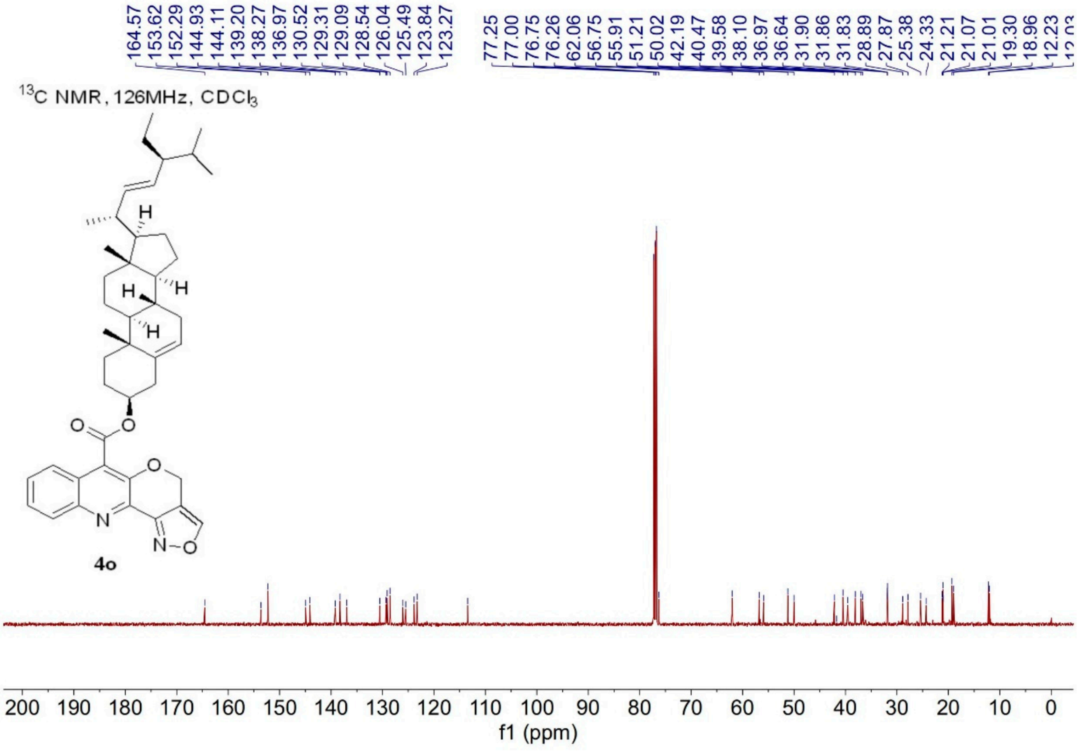
164.57 153.62 152.29 144.93 144.11 139.20 138.27 136.97 130.52 129.31 129.09 128.54 126.04 125.49 123.84 123.27
77.25 77.00 76.75 76.26 62.06 56.75 55.91 51.21 50.02 42.19 40.47 39.58 38.10 36.97 36.64 31.90 31.86 31.83 28.89 27.87 25.38 24.33 21.21 21.07 21.01 19.30 18.96 12.23
13C NMR, 126MHz, CDCl3
4o
200 190 180 170 160 150 140 130 120 110 100 90 80 70 60 50 40 30 20 10 0
f1 (ppm)

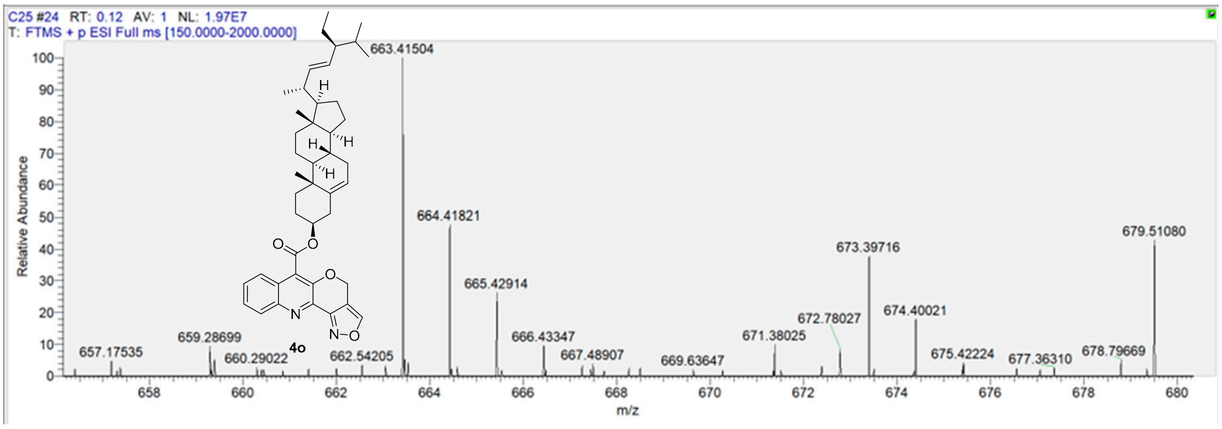
C25 #24 RT: 0.12 AV: 1 NL: 1.97E7
T: FTMS + p ESI Full ms [150.0000-2000.0000]
Relative Abundance
m/z
663.41504
664.41821
665.42914
666.43347
667.48907
669.63647
671.38025
672.78027
673.39716
674.40021
675.42224
677.36310
678.79669
679.51080
657.17535
659.28699
660.29022
662.54205
4o

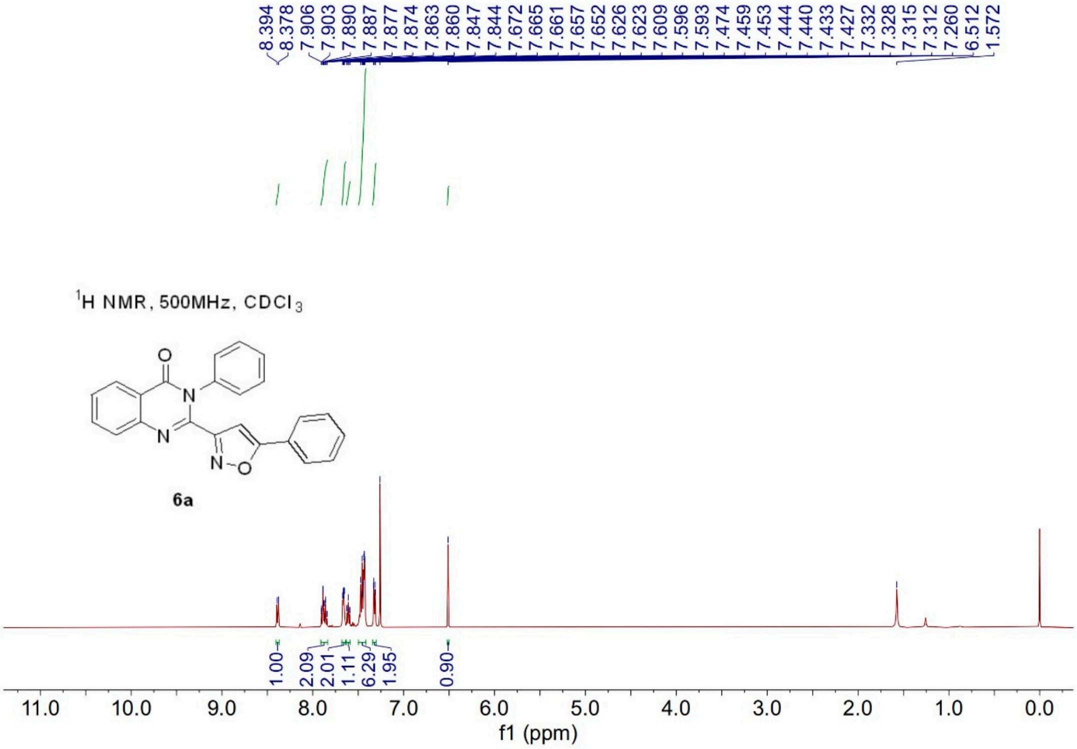
8.394
8.378
7.906
7.903
7.890
7.887
7.877
7.874
7.863
7.860
7.847
7.844
7.672
7.665
7.661
7.657
7.652
7.626
7.623
7.609
7.596
7.593
7.474
7.459
7.453
7.444
7.440
7.433
7.427
7.332
7.328
7.315
7.312
7.260
6.512
1.572
1H NMR, 500MHz, CDCl3
6a
1.00
2.09
2.01
1.11
6.29
1.95
0.90
f1 (ppm)

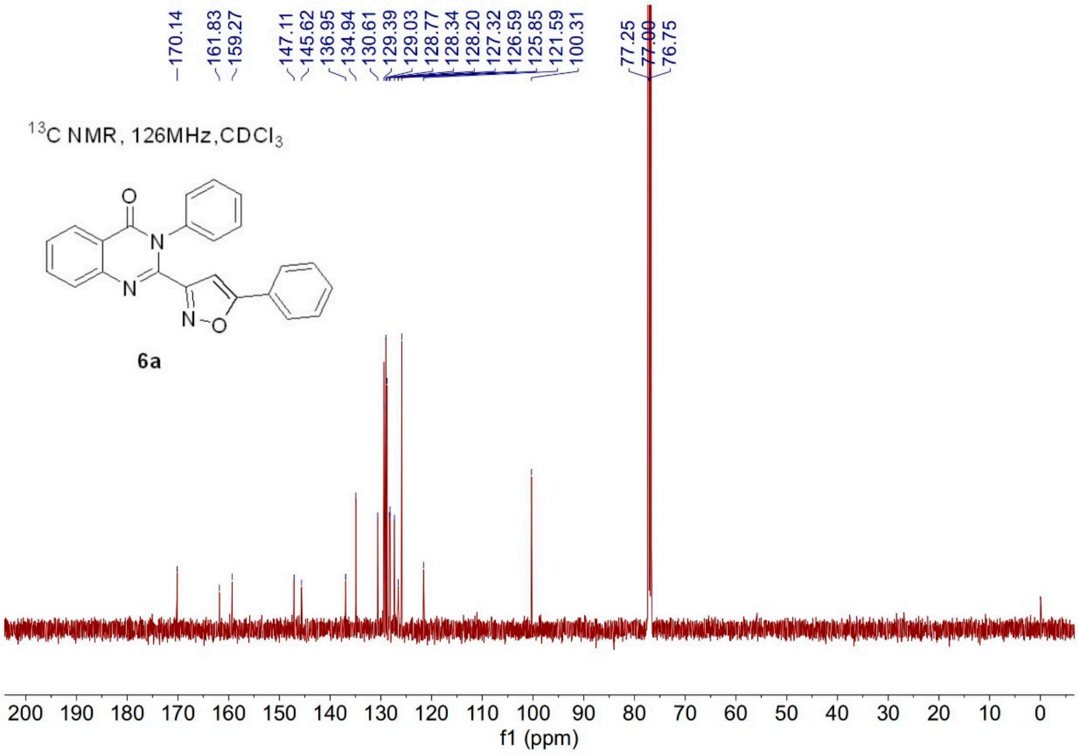

170.14
161.83
159.27
147.11
145.62
136.95
134.94
130.61
129.39
129.03
128.77
128.34
128.20
127.32
126.59
125.85
121.59
100.31
77.25
77.00
76.75
^{13}C NMR, 126MHz, CDCl$_3$
6a
200 190 180 170 160 150 140 130 120 110 100 90 80 70 60 50 40 30 20 10 0
f1 (ppm)

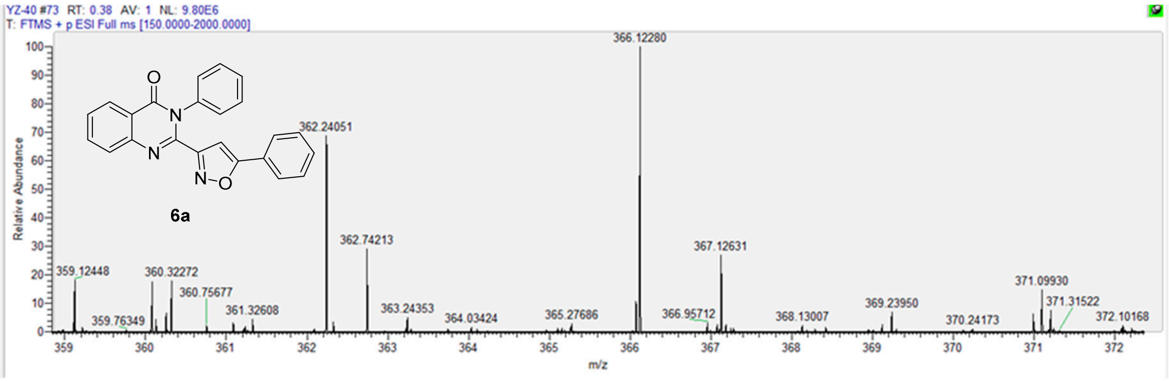

YZ-40 #73 RT: 0.38 AV: 1 NL: 9.80E6
T: FTMS + p ESI Full ms [150.0000-2000.0000]
Relative Abundance
6a
366.12280
362.24051
362.74213
367.12631
359.12448
360.32272
360.75677
361.32608
359.76349
363.24353
364.03424
365.27686
366.95712
368.13007
369.23950
370.24173
371.09930
371.31522
372.10168
m/z
